# Differentiation of *Cyanthillium cinereum*, a smoking cessation herb, from its adulterant *Emilia sonchifolia* using macroscopic and microscopic examination, HPTLC profiles and DNA barcodes

**DOI:** 10.1038/s41598-020-71702-7

**Published:** 2020-09-08

**Authors:** Kannika Thongkhao, Veerachai Pongkittiphan, Thatree Phadungcharoen, Chayapol Tungphatthong, Santhosh Kumar J. Urumarudappa, Thitima Pengsuparp, Narueporn Sutanthavibul, Worakorn Wiwatcharakornkul, Surapong Kengtong, Suchada Sukrong

**Affiliations:** 1 Research Unit of DNA Barcoding of Thai Medicinal Plants, Chulalongkorn University Drug and Health Products Innovation Promotion Center, Department of Pharmacognosy and Pharmaceutical Botany, Faculty of Pharmaceutical Sciences, Chulalongkorn University, Bangkok, 10330 Thailand; 2 College of Pharmacy, Rangsit University, Pathumthani, 12000 Thailand; 3 Department of Pharmacy Practice, Faculty of Pharmaceutical Sciences, Chulalongkorn University, Bangkok, 10330 Thailand; 4 Department of Pharmaceutics and Industrial Pharmacy, Faculty of Pharmaceutical Sciences, Chulalongkorn University, Bangkok, 10330 Thailand; 5 School of Pharmacy, Eastern Asia University, Bangkok, 12110 Thailand

**Keywords:** Biological techniques, Plant sciences

## Abstract

*Cyanthillium cinereum* (L.) H.Rob. is one of the most popular herbal smoking cessation aids currently used in Thailand, and its adulteration with *Emilia sonchifolia* (L.) DC. is often found in the herbal market. Therefore, the quality of the raw material must be considered. This work aimed to integrate macro- and microscopic, chemical and genetic authentication strategies to differentiate *C. cinereum* raw material from its adulterant. Different morphological features between *C. cinereum* and *E. sonchifolia* were simply recognized at the leaf base. For microscopic characteristics, trichome and pappus features were different between the two plants. HPTLC profiles showed a distinct band that could be used to unambiguously differentiate *C. cinereum* from *E. sonchifolia*. Four triterpenoid compounds, *β*-amyrin, taraxasterol, lupeol, and betulin, were identified from the distinct HPTLC band of *C. cinereum*. The use of core DNA barcode regions; *rbc*L, *mat*K, ITS and *psb*A-*trn*H provided species-level resolution to differentiate the two plants. Taken together, the integration of macroscopic and microscopic characterization, phytochemical analysis by HPTLC and DNA barcoding distinguished *C. cinereum* from *E. sonchifolia*. The signatures of *C. cinereum* obtained here can help manufacturers to increase the quality control of *C. cinereum* raw material in commercialized smoking cessation products.

## Introduction

Over 1.1 billion people smoke tobacco worldwide, which causes approximately 8 million deaths each year, as reported by the World Health Organization (WHO)^1^. According to the report, smoking-related deaths will increase if no effective smoking cessation policies are implemented. Currently, nicotine replacement and non-nicotine replacement products are utilized for smoking therapy. Various dosage forms of smoking cessation aids are available, such as teas, gums, inhalers, lozenges and nasal sprays. However, approximately 80% of smokers worldwide are living in low- and middle-income countries^1^, where cessation aid products are expensive, leading to unsuccessful attempts to quit. In addition, side effects such as dry mouth, nausea and sedation are associated with using cessation aids^2^.

**Figure 1:**
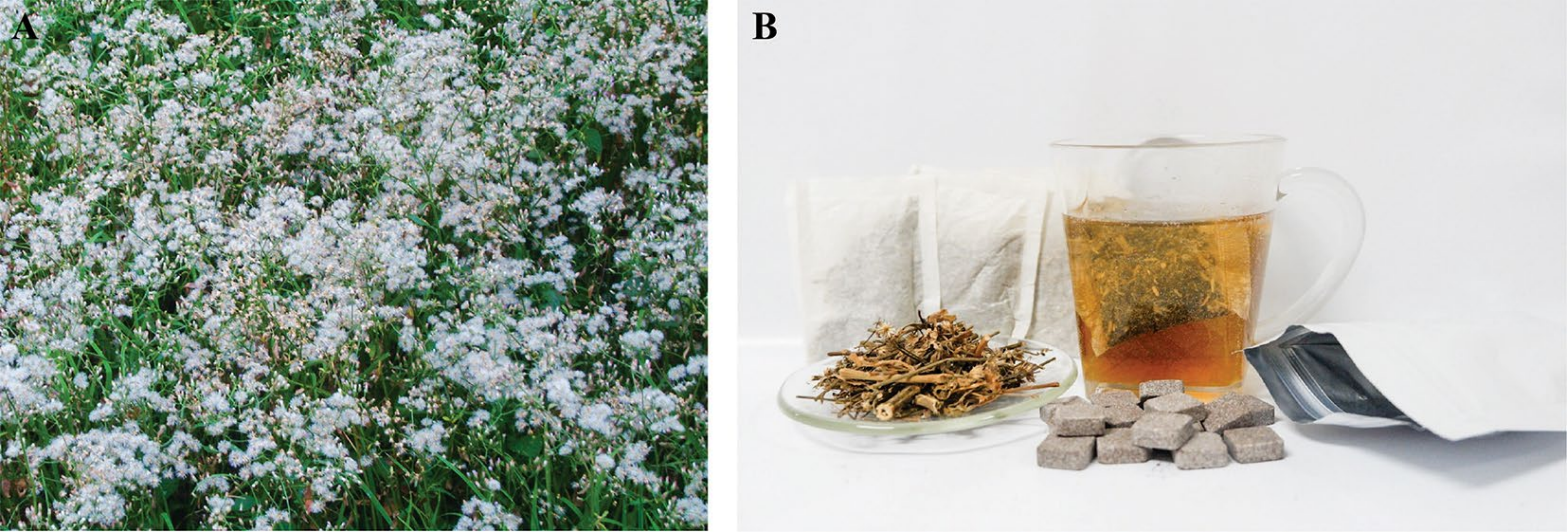
Plants and smoking cessation products. (**A**) *Cyanthillium cinereum* field, (**B**) raw material and smoking cessation products.

In recent years, traditional and complementary medicines have received growing interest worldwide. In Thailand, an herbal species in the Asteraceae family, *Cyanthillium cinereum* (L.) H.Rob. or *Vernonia cinerea* (L.) Less. (Thai: Ya dok khao) has been applied as a complementary medicine for the cessation of smoking (Fig. 1). *C. cinereum* tea has been included in the National List of Essential Medicines, Ministry of Public Health, Thailand, since 2012^3^. According to a previous report, *C. cinereum* may act as nicotine replacement therapy because the leaves and flowers contain tiny amounts of nicotine (0.154% and 0.123%, respectively)^4^. Furthermore, an abundance of nitrate salt is found in stem and leaf extracts of *C. cinereum* (21% and 19%, respectively)^4^. A study on the *C. cinereum* extract revealed that nitrate salt can cause tongue numbness and results in reduced cigarette smell and taste^5^. Moreover, supplementation of exercise with *C. cinereum* juice can reduce oxidative stress and the number of cigarettes smoked per day in volunteer smokers^6^.

*Emilia sonchifolia* (L.) DC. (Thai: Hu pla chon), also in the Asteraceae family, shares similar flower and fruit characteristics with *C. cinereum*. Moreover, the plant is often found in the same habitat as *C. cinereum*. Careless collection practices and lack of standard quality control lead to contamination by *E. sonchifolia* and result in substandard quality of the smoking cessation aid products; therefore, appropriate markers for quality control of herbal raw materials are necessary. In addition, products should be processed with good agricultural practices (GAPs) and good manufacturing processes (GMPs) to ensure sustainable agriculture and ecological safety. Concrete policy or guideline frameworks for herbal products, including agricultural and manufacturing practices, product labeling, product registration, marketing and trade, would be seriously regulated.

Authentication of raw materials can be performed by different methods. The standard methods are micro- and macroscopic characterization and phytochemical analysis. To complement traditional identification methods, innovative technologies such as DNA barcoding are also used. Macroscopic and microscopic methods for botanical identification are recommended as basic tools in the Thai Herbal Pharmacopoeia (THP)^7^. The methods are simple, rapid and inexpensive, but they must be performed by trained experts, and reliable references are required^8^. Chemical detection methods may produce uncertain results due to environmental factors that affect the chemical composition of herbal species and biological activities of the substances. Approaches such as thin-layer chromatography (TLC) or high-performance thin-layer chromatography (HPTLC) are versatile and specific for the identification of phytochemical constituents in herbal products. However, standard substances are required for analytical methods to yield quantitative benefits^9^. Although DNA testing is a high-cost technique, it yields precise and reliable results because herbal genetics do not vary with environmental factors^10^. DNA testing is also applicable for limited sources of samples. As mentioned above, each analysis method has advantages and limitations, and combined methods should be applied to control the quality of herbal raw materials for consumer safety and to meet international standards. Therefore, this study aimed to differentiate *C. cinereum*, a smoking cessation herb, from its adulterant, *E. sonchifolia*, using macroscopic and microscopic examination, HPTLC profiling and DNA barcoding methods.

## Results

### Macroscopic characteristics

**Figure 2:**
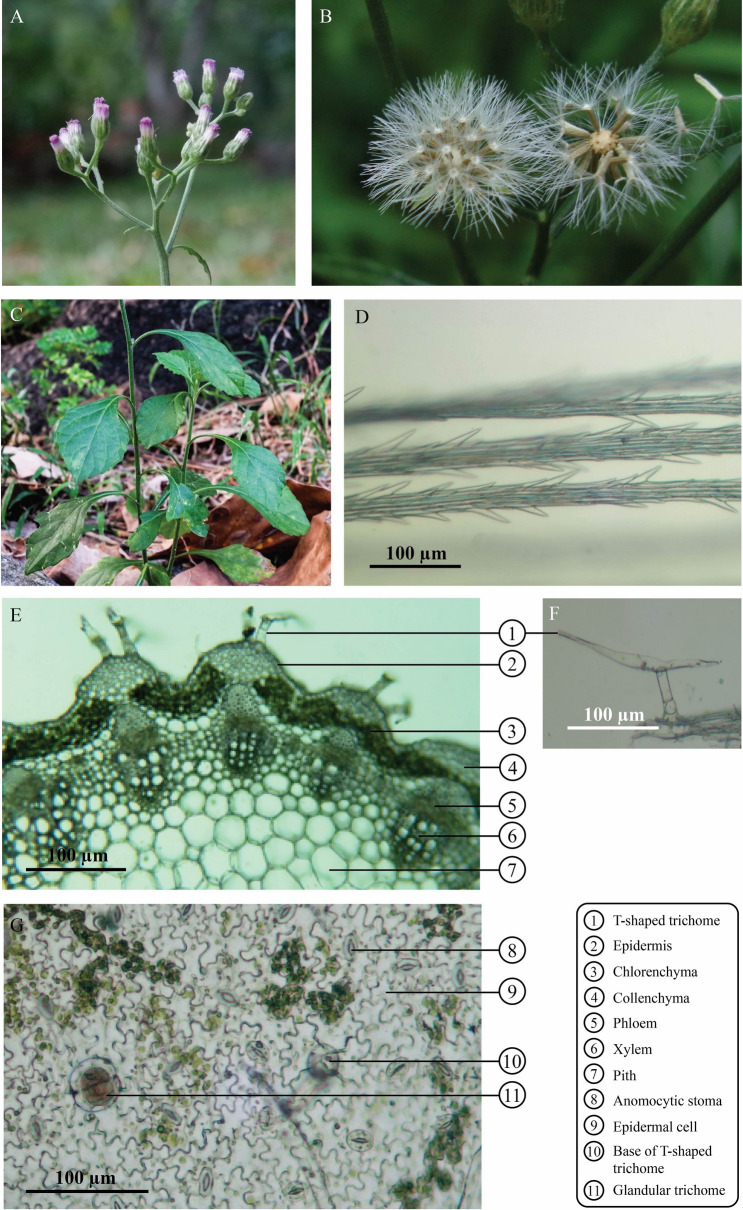
Macroscopic and microscopic characteristics of *C. cinereum*. (**A**) Inflorescence, (**B**) infructescence, (**C**) leaves, (**D**) pappus with trichomes, (**E**) transverse section of stem; 1: T-shaped trichome; 2: epidermis; 3: chlorenchyma; 4: collenchyma; 5: phloem; 6: xylem; 7: pith, (**F**) T-shaped trichome, (**G**) lower epidermis of leaf; 8: anomocytic stoma; 9: epidermal cell; 10: cicatrix of T-shaped trichome; 11: glandular trichome.

**Figure 3:**
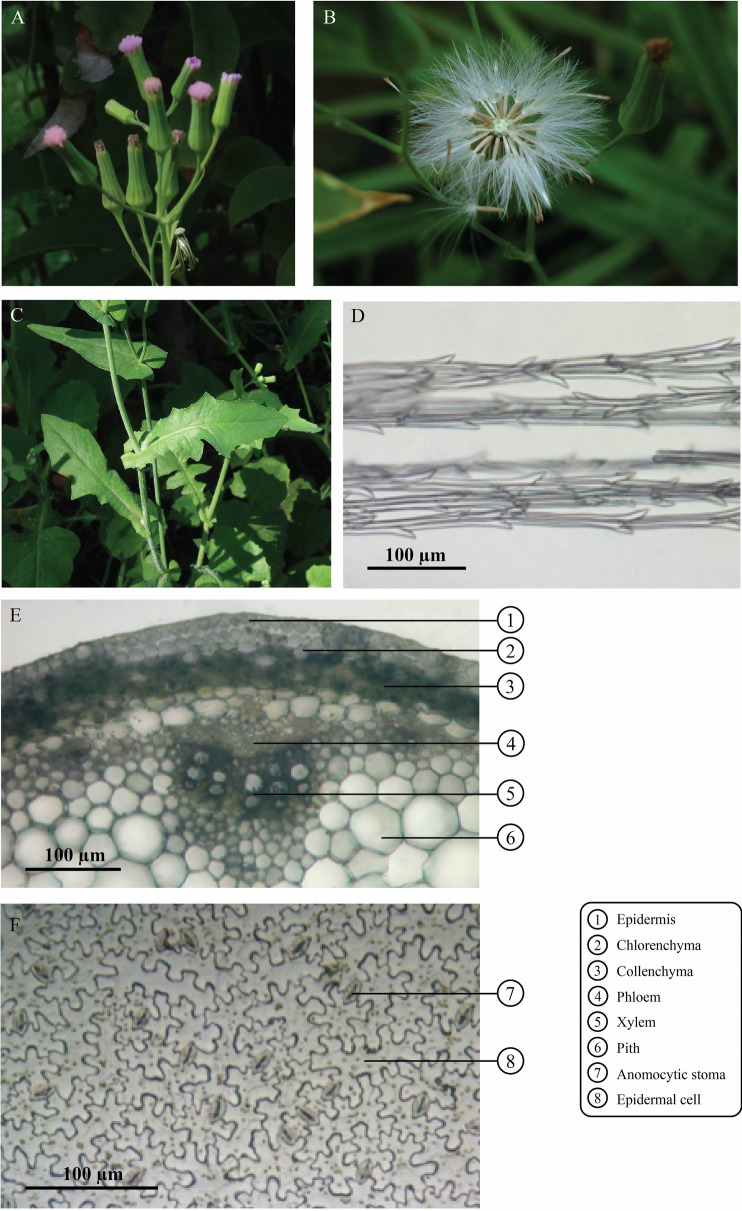
Macroscopic and microscopic characteristics of *E. sonchifolia*. (**A**) Inflorescence, (**B**) infructescence, (**C**) leaves, (**D**) pappus with trichomes, (**E**) transverse section of stem; 1: epidermis; 2: chlorenchyma; 3: collenchyma; 4: phloem; 5: xylem; 6: pith, (**F**) lower epidermis of leaf; 7: anomocytic stoma; 8: epidermal cell.

*C. cinereum* and *E. sonchifolia* plants are slender-stemmed herbs in the Asteraceae family. Flowers of *C. cinereum* look very similar to *E. sonchifolia* flowers. Individual plants produce capitula with purple or white florets. However, distinct characteristics can be observed by macroscopic examination. *C. cinereum* flower-heads are tubulate with pink to purple florets (Fig. 2A), while long vase-shaped flower bracts with petals tinted pink to purple are observed in *E. sonchifolia* (Fig. 3A). The fruit of *C. cinereum* is a subcylindrical linear achene covered with short hairs (Fig. 2B), while that of *E. sonchifolia* is an achene with longitudinal ribs, short hairs and surmounted by white pappus (Fig. 3B). Serrated leaves with attenuated bases are found in *C. cinereum* (Fig. 2C), while auriculate leaf bases and triangulate or pinnate-lobed lower leaves and arrow-shaped upper leaves with bases encircling the stem are present in *E. sonchifolia* (Fig. 3C). Dried herbs of *C. cinereum* are greenish brown, while those of *E. sonchifolia* exhibit a light green color. Grass-like odor is observed in both dried herbs, but *C. cinereum* express a stronger odor than that of *E. sonchifolia*. The taste of both dried *C. cinereum* and *E. sonchifolia* is slightly bitter*.*

### Microscopic features

Microscopic studies showed the anatomical features of *C. cinereum* and *E. sonchifolia*. The fruit pappi of both plants were examined under a microscope. The pappi resembled fine feathery hairs of calyx consisting of one to several rows of thin-walled elongate cells and unicellular trichomes in both plants. Pappi of *C. cinereum* exhibited more than 3 rows of thin-walled elongated cells, while 2 to 3 rows with unicellular trichomes were found in *E. sonchifolia*. The unicellular trichomes of *C. cinereum* pappi were longer than those of *E. sonchifolia* (Fig. 2D and Fig. 3D). Transverse sections of stems showed epidermis with a layer of rectangular cells in both *C. cinereum* and *E. sonchifolia* (Fig. 2E). Two types of trichomes, T-shaped and glandular trichomes, were observed in transverse sections of the stem of *C. cinereum* (Fig. 2E,F), while long, multicellular, uniseriate trichomes were found in *E. sonchifolia.* The cortex contained 2–4 layers of collenchyma cells in *C. cinereum* and 1–3 layers in *E. sonchifolia*. Pith cells of *C. cinereum* were composed of large ordinary parenchyma cells and rosette aggregates of calcium oxalate crystals (Fig. 2E). The stele of *E. sonchifolia* showed phloem and xylem, including pith cells with large ordinary parenchyma cells (Fig. 3E). Investigation of leaves showed that the lower epidermis of *C. cinereum* was composed of wavy-walled cells, including numerous anomocytic stomata (Fig. 2F), similar to *E. sonchifolia* (Fig. 3F). The base of T-shaped trichomes and glandular trichomes were also observed in *C. cinereum* leaves (Fig. 2G). Microscopic study revealed T-shaped trichome, glandular trichomes, pappus and pollen grain in *C. cinereum* tea product (Fig. 6).

### HPTLC fingerprint profiles

**Figure 4:**
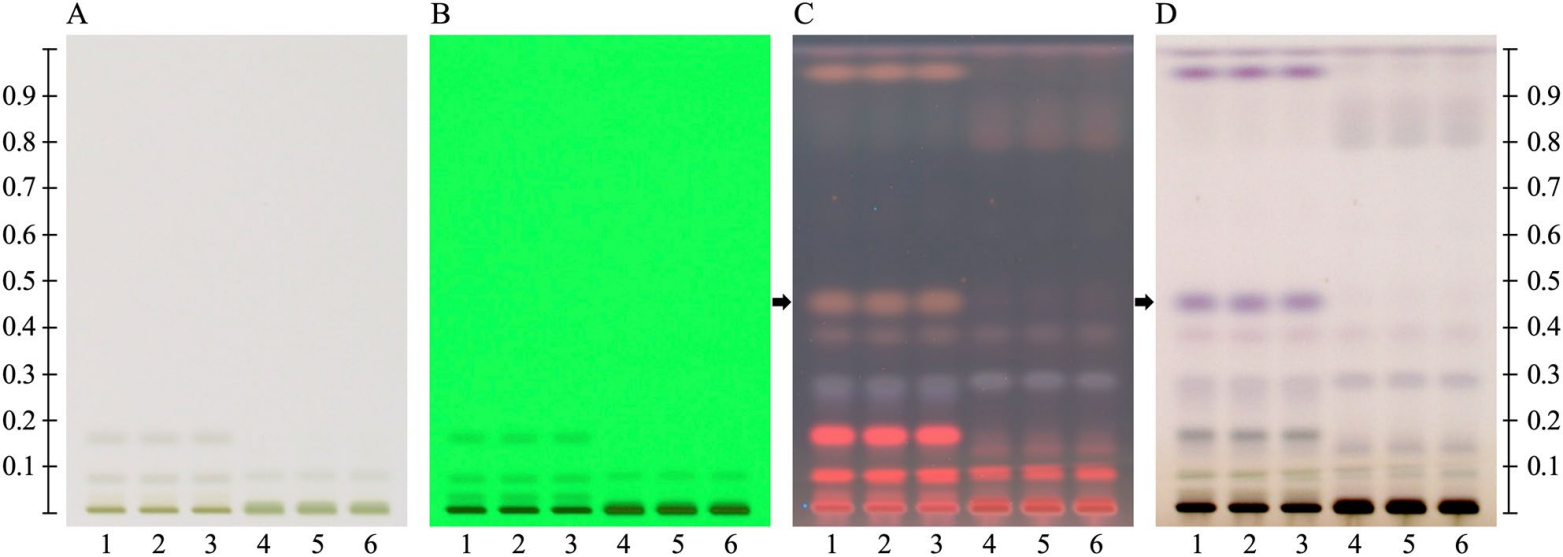
High-performance thin-layer chromatography (HPTLC) chromatograms of representative samples of *C. cinereum* (tracks 1–3) and *E. sonchifolia* (tracks 4–6) extracts. (**A**) Under white light, (**B**) under UV at 254 nm, (**C**) under UV at 366 nm, and (**D**) under white light after postchromatographic derivatization with anisaldehyde-sulfuric acid reagent. Hexane:acetone mixture (1:9, v/v) was used as the mobile phase. The arrows in C and D show the presence of bands at Rf = 0.48 only in *C. cinereum*.

HPTLC profiles of ethanolic extracts of *C. cinereum* and *E. sonchifolia* were obtained. The mobile phase was a mixture of hexane and acetone (9:1, v/v). In general, the HPTLC patterns of *C. cinereum* and *E. sonchifolia* were similar. However, a distinct band at Rf = 0.48 was detected in only *C. cinereum* extract after plates were sprayed with anisaldehyde-sulfuric acid detecting reagent (Fig. 4) and the band was subjected to GC–MS analysis. A combination of the four triterpenoid compounds *β*-amyrin, taraxasterol, lupeol, and betulin was identified in *C. cinereum*.

### DNA barcoding analysis

**Figure 5:**
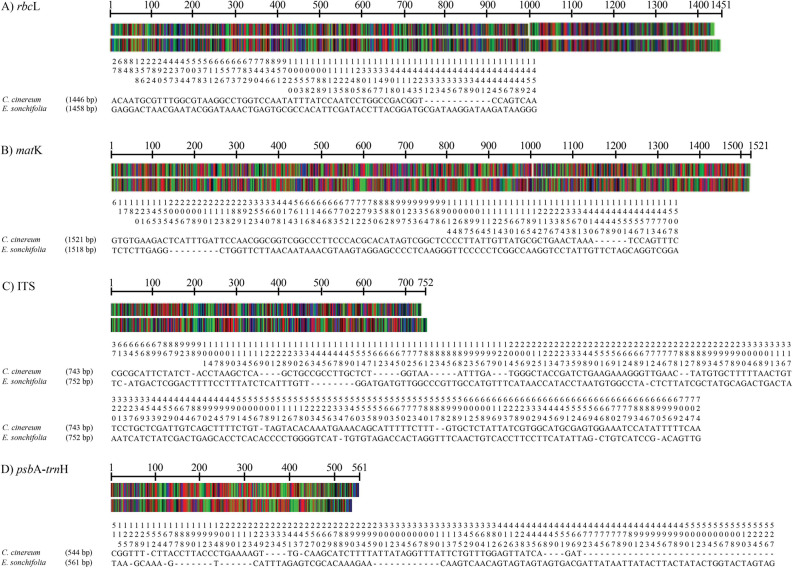
Illustration comparing core DNA barcodes of *C. cinereum* and *E. sonchifolia*. Polymorphic sites and indels in each DNA barcode area are shown; (**A**) *rbc*L, (**B**) *mat*K, (**C**) ITS and (**D**) *psb*A-*trn*H intergenic spacer. The nucleotide positions of the DNA barcode regions are indicated.

To distinguish *C. cinereum* from its adulterant *E. sonchifolia*, the nucleotide sequences of the four core DNA barcode regions, *rbc*L, *mat*K, ITS and *psb*A-*trn*H intergenic spacer, were analyzed. PCR amplicons of each barcode region were successfully amplified and sequenced. All plant specimens in the same species but from different collection places showed identical DNA sequences. There is no intraspecific divergence for all four barcode regions of both species. The length of the amplified product of each barcode region of the two species varied (Table 1). GenBank accession numbers were obtained (Table 2). The sequences of all DNA barcode areas exhibited indels (Fig. 5 and Supplementary Data S1–S4). The percentage of sequence variation among the four barcode regions was ranked as follows: ITS > *psb*A-*trn*H intergenic spacer > *mat*K > *rbc*L. The percent GC content among the same barcode areas of *C. cinereum* and *E. sonchifolia* was similar*.* The highest percent GC content was observed in the ITS region, followed by *rbc*L, *mat*K and *psb*A-*trn*H intergenic spacer. The percentage of variation was calculated, and the highest percent variation was found in the ITS region (29.24%), followed by the *psb*A-*trn*H intergenic spacer (19.90%), *mat*K (6.55%) and *rbc*L (5.13%). The highest interspecific divergence was found in the ITS region (32.38%), while the *psb*A-*trn*H intergenic spacer, *mat*K and *rbc*L exhibited values of 9.51%, 5.86% and 4.21%, respectively.

**Table 1:** Sequence analysis comparing core DNA barcode regions of *C. cinereum* and *E. sonchifolia*.

	*rbc*L	*mat*K	ITS	*psb*A-*trn*H spacer
**Length (bp)**				
*C. cinereum*	1,146	1521	743	544
*E. sonchifolia*	1,458	1518	752	561
**GC content (%)**				
*C. cinereum*	43.70	33.26	44.01	29.23
*E. sonchifolia*	43.48	33.26	44.94	29.23
**No. of variations (bp)**	75	100	224	117
Nucleotide variation (%):	5.13	6.55	29.24	19.90
Interspecific divergence (%)	4.21	5.86	32.38	9.51

**Table 2:** Authentic plant species used in this study and the accession numbers of their DNA barcode loci deposited in GenBank.

Authentic species	Voucher No	Province	Accession number			
			*rbc*L	*mat*K	ITS	*psb*A-*trn*H
*C. cinereum*	SS-642	Nakhon Pathom	LC503541	LC503540	LC503539	LC503542
	SS-643	Nakhon Pathom	LC503549	LC503548	LC503547	LC503550
	SS-644	Nakhon Pathom	LC503557	LC503556	LC503555	LC503558
	SS-645	Bangkok	LC503565	LC503564	LC503563	LC503566
	SS-646	Surat Thani	LC503573	LC503572	LC503571	LC503574
	SS-647	Chiang Rai	LC503581	LC503580	LC503579	LC503582
	SS-648	Prachinburi	LC503589	LC503588	LC503587	LC503590
*E. sonchifolia*	SS-649	Nakhon Pathom	LC503545	LC503544	LC503543	LC503546
	SS-650	Bangkok	LC503553	LC503552	LC503551	LC503554
	SS-651	Bangkok	LC503561	LC503560	LC503559	LC503562
	SS-652	Chiang Rai	LC503569	LC503568	LC503567	LC503570
	SS-653	Prachinburi	LC503577	LC503576	LC503575	LC503578
	SS-654	Surat Thani	LC503585	LC503584	LC503583	LC503586
	SS-655	Prachinburi	LC503593	LC503592	LC503591	LC503594

## Discussion

Several approaches have been applied to help smokers quit smoking. Smoking cessation aids can be medications or non-medicinal treatments. Nicotine replacement therapy (NRT) products, varenicline and bupropion are FDA-approved medications to help users manage withdrawal^3^. However, the cost of medications remains expensive, especially for smokers who live in low- or middle-income countries. Another disadvantage of using medications is their side effects, such as nausea, dry mouth, increased appetite, insomnia and sedation^11^. Therefore, non-medicinal approaches, such as the use of local herbs for smoking cessation, have gained increasing attention because they provide a low cost of treatment with a high percentage of successful quitting of smoking^12^. It has been suggested that regular drinking of herbal teas containing high antioxidant levels may protect smokers from oxidative damage caused by smoking^13^. A report revealed that *Syzygium aromaticum* (L.) Merr. & L.M.Perry and *Astragalus mongholicus* Bunge teas, which have high antioxidant activity, can reduce smoking withdrawal symptoms^13^. High antioxidant activity is observed in many parts of *C. cinereum*, as reported by Ketsuwan et al.^4^. Moreover, a study of *C. cinereum* and lime in Thailand demonstrated that the herb can reduce smoking urges in two weeks and decrease the number of cigarettes smoked^12^. Furthermore, the abstinence rate tends to increase in users who drink *C. cinereum* tea compared with those who take a placebo^14^. In addition to smoking cessation, *C. cinereum* has been used as a traditional medicine to cure skin diseases, including cough, bronchitis, asthma, malaria, cancer, gastrointestinal disorder, diuresis, pain, and diabetes^15,16^. A wide range of biological, pharmacological, antimicrobial, anti-inflammatory and anticancer activities have been reported for *C. cinereum* in numerous studies^17,18^. Letha et al.^19^ showed that *C. cinereum* extract exhibits no significant toxicity in mice and brine shrimp.

It needs to be emphasized that *C. cinereum* is a common weed, it is not cultivated, and the plant is often found in the same habitat as *E. sonchifolia*. Species analysis based on morphological characteristics, including aerial parts, flowers and fruits, revealed several similar features between the two species. Careless herbal collection will lead to mixing the two species. In 2018, a local herbal company successfully developed a smoking cessation lozenge from *C. cinereum*. One large-scale delivery of *C. cinereum* raw material received from local collectors, however, was contaminated with *E. sonchifolia*. This contamination resulted in the rejection of the material (unpublished data). *E. sonchifolia* has been reported to contain pyrrolizidine alkaloids (a class of hepatotoxic and tumorigenic compounds) and has caused food poisoning in several countries^20^. Eleven pyrrolizidine alkaloid compounds have been identified from *E. sonchifolia* aqueous extract, and senkirkine was found as the major pyrrolizidine alkaloid in dry herbs^21^. Moreover, up to 0.2% of doronine and senkirkine from *E. sonchifolia* may cause significant intoxication^22^. Therefore, to prevent the adulteration and contamination of *C. cinereum* materials by *E. sonchifolia*, a reliable differentiation tool is needed. In addition, the tool should facilitate herbal production of *C. cinereum* that complies with GAPs and GMPs.

**Figure 6:**
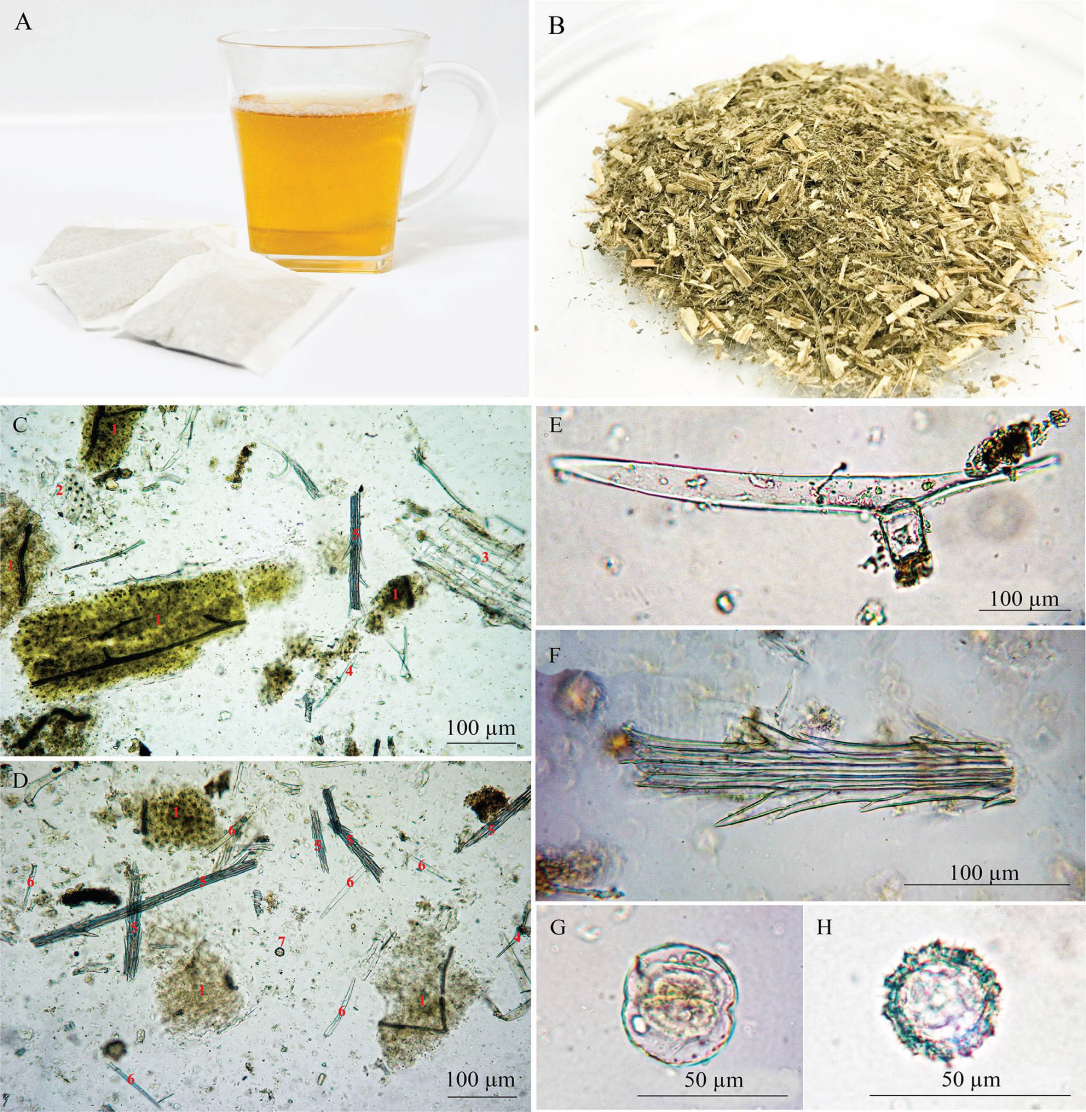
*C. cinereum* smoking cessation tea product and its photomicrographs. (**A**,**B**) *C. cinereum* tea product, (**C**,**D**) overall cell components; 1: fragment of lamina in surface view; 2: lamina in surface view; 3: parenchyma of stem; 4: T-shaped trichome; 5: pappus; 6: fragment of multicellular uniseriate trichome of petal; 7: pollen grain, (**E**) T-shaped trichome, (**F**) pappus, (**G**) glandular trichome and (**H**) pollen grain.

As mentioned above, *C. cinereum* and *E. sonchifolia* exhibit similar morphological features, but there is an obvious differential characteristic at the leaf base. An auriculate leaf base is the main characteristic of *E. sonchifolia*^23^, while *C. cinereum* has a simple, alternating and variable leaf shape. As dry herbs and powder of *C. cinereum* are utilized for smoking cessation products, it is difficult to identify their origin when the adulterant is mixed in. According to the THP, various techniques, including macroscopic, microscopic and TLC identification, are recommended for medicinal plant authentication. Therefore, in this study, several markers of *C. cinereum* were identified by following THP guidelines. Microscopic authentication revealed that the anomocytic type of stomata is common in both species*.* Interestingly, *C. cinereum* contained two types of trichomes: T-shaped and glandular. An abundance of glandular and T-shaped trichomes was observed in *C. cinereum* leaves, while there was no specific type of trichome present in *E. sonchifolia* leaves. This finding suggests that the trichomes may be a promising characteristic for the differentiation of *C. cinereum* from *E. sonchifolia* raw materials by microscopic observation. Furthermore, our developed macro- and microscopic identification methods for the differentiation of the two species was applied to test whether a commercial smoking cessation tea product purchased from a local market contained the authentic plant species or a substitution. Greenish brown herbal materials with grass-like odor and slightly bitter taste from the tea product were observed. It is difficult to identify the plant species of samples by observing only their macroscopic and organoleptic characteristics. However, under microscopic observation, T-shaped and glandular trichomes, which are unique characters of *C. cinereum,* were clearly visible in the commercial product (Fig. 6), confirming the authenticity of the *C. cinereum* tea product. These results are supported by previous studies showing that trichome characteristics can be useful for the differentiation of species with similar ecological or geographical features^24^.

Phytochemical substances such as steroids, saponins, alkaloids, carbohydrates, flavonoids, phenols, tannins, nitrate and proteins have been reported in *C. cinereum* extracts^25^. In this study, a distinct band from *C. cinereum* extract was identified by GC–MS (Supplementary Figure S1), and its mass spectrum was run against reference mass spectra in the NIST2011 library via spectrum matching, resulting in its identification as a mixture of four triterpenoid compounds: *β*-amyrin, taraxasterol, lupeol, and betulin. Our finding agrees with those of Rao and Bose (1962), who found *β*-amyrin, lupeol and their acetate derivatives in whole-plant extracts of *C. cinereum*^26^. In addition to the four triterpenoid compounds obtained in this study, two other triterpenoid compounds, stigmast-5,17(20)-diene-3*β*-ol and 24-hydroxytaraxer-14-ene, are also present in extracts of *C. cinereum* according to a previous report^27^. This is the first study to identify taraxasterol and betulin from *C. cinereum*. Therefore, the four triterpenoids could be used as chemical markers for the differentiation of *C. cinereum* from *E. sonchifolia*.

In addition to the traditional methods recommended in the THP, DNA barcoding with selected gene candidates, such as *mat*K, *rbc*L, ITS, and *psb*A-*trn*H spacer, has been applied for discrimination of plant species^28–30^. In this study, DNA barcoding analysis was performed for comparison of *C. cinereum* and *E. sonchifolia* sequences. Nucleotide polymorphisms and indels were found in all DNA barcode areas. The *rbc*L gene revealed the lowest nucleotide variation (5.13%) among the DNA barcode regions, which correlated with results from previous work^28^. Recently, Veldman et al.^29^ showed that *rbc*L exhibited very low discriminatory power, making it suitable for identification of plants at the family level. In this study, the full-length *mat*K sequence was obtained even though it has been reported that *mat*K has a low sequencing success rate in medicinal plants because of low primer universality^30^. The highest nucleotide variation in the two plant species was observed in the ITS region. The ITS sequence contains highly variable sites with the highest interspecific divergence (32.38%) among the four core barcode areas. This finding is consistent with the fact that the ITS region shows greater discriminatory power than the plastid regions and is useful for the authentication of plants at the species level^31–33^. The *psb*A-*trn*H spacer, a noncoding region, has a high number of nucleotide variations between *C. cinereum* and *E. sonchifolia*. This spacer is found to be the best candidate for discrimination at the species and subspecies levels^34^. In a previous report, a two-tiered approach to DNA barcoding was suggested for species identification^35^. Herbal species, including plant fillers composed of herbal supplements, were successfully identified with a two-tiered approach using both the *rbc*L and ITS2 regions^36^. A two-locus combination of the *psb*A-*trn*H intergenic spacer and ITS2 regions was also a powerful tool for herbal authentication, and this strategy was recommended for the identification of closely related species^37^.

Since adulterant problems frequently occur due to the morphological similarity of raw materials, efficient and effective methods to identify and monitor the origins of plants are needed. In this study, integrative analysis by macroscopic, microscopic, chemical and molecular markers is proposed for the first time to differentiate raw materials of *C. cinereum*, a smoking cessation herb, from the adulterant *E. sonchifolia*. In herbal products or mixed raw materials, however, it is challenging to distinguish *C. cinereum* from *E. sonchifolia*. Mixed chemical profiles and specific characteristics of those two plants will be observed if the adulterant is mixed in. Combination of DNA barcoding with advance technique such as Next-Generation Sequencing (NGS) would be beneficial for examination of the admixture. As herbal authentication is a crucial step, confirmation of plant identity with integrative methods should be exploited as a part of quality control for the safety and efficacy of the herb or its products. To protect consumers, policy or guideline frameworks involving good practices for collecting herbal raw material and manufacturing should be seriously considered by regulatory agencies.

## Methods

### Sample collections

*C. cinereum* and *E. sonchifolia* were collected from different natural places in Thailand. Raw *C. cinereum* materials were kindly provided by an herbal company. Sample details along with their voucher numbers and places of collection were recorded (Table 2). All plant samples were identified by the taxonomist Assoc. Prof. Thatree Phadungcharoen at Rangsit University, Pathumthani, Thailand. Each voucher specimen was assigned a specific number and deposited at the Museum of Natural Medicine, Chulalongkorn University, Thailand.

### Macroscopic and microscopic characterization of *C. cinereum* and *E. sonchifolia*

The macroscopic and microscopic diagnostic features of *C. cinereum* and *E. sonchifolia* were established. Transverse sections of stems and leaves were prepared using sharp razor blades. Sections were mounted with glycerin and subsequently examined under a light microscope (Olympus CH, Japan). Stem and leaf features were observed by 400 × lens magnification under a microscope, and photographs were taken. Slides were prepared in triplicates. Key microscopic features of *C. cinereum* product were diagnostic under the same conditions as described above.

### High-performance thin-layer chromatography of *C. cinereum* and *E. sonchifolia*

Whole plants of *C. cinereum* and *E. sonchifolia* were dried at 50 °C for 3 days before being crushed into powder and subjected to HPTLC analysis. Subsequently, 20 g of fine powder was extracted in 20 ml of ethanol by immersing the powder in ethanol at room temperature for 48 h. Centrifugation was performed at 10,000 rpm for 10 min at 25 °C, and the supernatant was collected. Extraction solution (3 µL) was sprayed on an HPTLC plate (10 × 10 cm, silica gel 60 F_254_, Merck, Germany) using an Automatic TLC Sampler 4 (AST4, CAMAG, Muttenz, Switzerland). Each band was 4 mm in length, while the distance between lanes was 2 mm. The distance from the left side was 15 mm, and the distance from the bottom edge was 15 mm. A hexane:acetone mixture (9:1, v/v) was used as the mobile phase. The HPTLC chamber was saturated with 20 ml of the mobile phase for 20 min before development. HPTLC plates were sprayed with anisaldehyde-sulfuric acid reagent and heated at 105 °C to detect phytochemical constituents. Plates were visualized and imaged under short (254 nm) and long (366 nm) ultraviolet (UV) wavelengths.

### Phytochemical markers to differentiate *C. cinereum* from *E. sonchifolia*

In HPTLC analysis, an unambiguous band unique to *C. cinereum* was subjected to GC–MS experiments to identify the chemical. The analysis was performed on a GC/MS instrument (triple quadrupole GC/MS (GC-QQQ), Agilent Technologies, Agilent 7890B GC system and Agilent 7000C GC/MS Triple Quad) equipped with an HP-5 ms (length 30 m × inner diameter (ID) 0.25 mm, film thickness 0.25 µm) porous-layer open-tubular (PLOT) column. The split ratio was 1:10. The temperature of the injection port was 280 °C. The flow rate of the helium carrier gas was maintained at a constant 1.0 mL/min. The column temperature was held at 40 °C for 2 min to concentrate the hydrocarbon at the head of the column; subsequently, the column temperature was increased to 150 °C, ramped at 25 °C/min to 300 °C, and held at 300 °C for 15 min. The MS analyses were performed in full scan mode in electrospray ionization (EI) mode with a scan range of m/z 33–600. The ion source was maintained at 70 °C, and an ionization energy of 230 eV was used for each measurement. Compounds were identified by query mass spectrum matching with the NIST2011 reference library (National Institute of Standards and Technology, Gaithersburg, MD, USA).

### DNA barcoding of *C. cinereum* and *E. sonchifolia*

#### Genomic DNA extraction

Young leaves of authentic *C. cinereum* and *E. sonchifolia* were collected and ground into fine powder by mortar and pestle. Genomic DNA from both plant species was extracted from 80 to 100 mg powder samples using a DNeasy Plant Mini Kit (Qiagen, Germany) following the manufacturer’s instructions. Subsequently, the genomic DNA was further purified using a GENECLEAN Kit (MP Biomedicals, USA) according to the manufacturer’s protocol. The quantity and quality of the purified genomic DNA were determined using a NanoDrop One UV–Vis spectrophotometer (Thermo Scientific, USA) and agarose gel electrophoresis, respectively. Nucleic acids were visualized under UV light (Bio-Rad Gel Doc XR Imaging system, USA) and imaged by using Analysis Quantity One version 4.6.8. Purified genomic DNA was kept at −20 °C for further analysis.

#### DNA barcoding of authentic *C. cinereum* and *E. sonchifolia*

Four core DNA barcode regions, ribulose-1,5-bisphosphate carboxylase/oxygenase large subunit (*rbc*L), maturase K (*mat*K), internal transcribed spacer (ITS) and *trn*H-*psb*A intergenic spacer, were assessed by PCR. The DNA barcode regions were amplified by universal primers (see Supplementary Table S1 online). PCR amplifications were performed in 25 µL of reaction mixture consisting of 1X PCR buffer with 1.5 mM MgCl_2_, 0.2 mM dNTP mix, 0.5 µM each forward and reverse primer, 0.5 U of Platinum *Taq* DNA polymerase (Invitrogen, USA) and 50 ng of genomic DNA. PCR was carried out in a T100 Thermal Cycler (Bio-Rad, USA) using 94 °C for 2 min; followed by 30 cycles of 94 °C for 30 s, 57 °C for 30 s, and 72 °C for 1 min (for *mat*K and *rbc*L) or 30 s (for ITS and *psb*A-*trn*H spacer); and a final extension at 72 °C for 10 min. Successful PCR amplifications were observed by 1.2% agarose gel electrophoresis in 1X TAE buffer and imaged using a Gel Doc XR system (Bio-Rad, USA). PCR amplicons were purified and subsequently bidirectionally sequenced by Sanger sequencing on an ABI 3730XL DNA analyzer. The same primer sets used for DNA barcode generation were used for sequencing steps. Nucleotide sequence results were edited by BioEdit software version 7.2.6. DNA sequences were aligned by MUSCLE. The alignment parameters were set as follows: gap open = -400, gap extend = 0, clustering methods = UPGMB and min diag. length = 24. The DNA barcode sequences were deposited in GenBank at the National Center for Biotechnology Information (NCBI). Polymorphisms, nucleotide variation and interspecific divergence of all DNA barcode regions between *C. cinereum* and *E. sonchifolia* were analyzed.

## Supplementary information

Supplementary information
