# Supplementary material for: Differentiation of Cyanthillium cinereum, a smoking cessation herb, from its adulterant Emilia sonchifolia using macroscopic and microscopic examination, HPTLC profiles and DNA barcodes
Source: Sci Rep. 2020 Sep 8;10:14753. doi: 10.1038/s41598-020-71702-7 (PMC7479599; doi:10.1038/s41598-020-71702-7)
Supplement: Supplementary file 1 — Supplementary information [file 41598_2020_71702_MOESM1_ESM.pdf]

Supplementary information

Differentiation of *Cyanthillium cinereum*, a smoking cessation herb, from its adulterant *Emilia sonchifolia* using macroscopic and microscopic examination, HPTLC profiles and DNA barcodes

Kannika Thongkhao[1], Veerachai Pongkittiphan[1], Thatree Phadungcharoen[2], Chayapol Tungphatthong[1], Santhosh Kumar J. Urumarudappa[1], Thitima Pengsuparp[3], Narueporn Sutanthavibul[4], Worakorn Wiwatcharakornkul[1], Surapong Kengtong[5] and Suchada Sukrong[1,*]

[1]Research Unit of DNA barcoding of Thai Medicinal Plants, Chulalongkorn University Drug and Health Product Innovation Promotion Center, Faculty of Pharmaceutical Sciences, Chulalongkorn University, Bangkok, 10330, Thailand

[2]College of Pharmacy, Rangsit University, Pathumthani, 12000, Thailand

[3]Department of Pharmacy Practice, Faculty of Pharmaceutical Sciences, Chulalongkorn University, Bangkok, 10330, Thailand

[4]Department of Pharmaceutics and Industrial Pharmacy, Faculty of Pharmaceutical Sciences, Chulalongkorn University, Bangkok, 10330, Thailand

[5]School of Pharmacy, Eastern Asia University, Bangkok, 12110, Thailand

*Corresponding Author:
Suchada Sukrong, Ph.D.
Department of Pharmacognosy and Pharmaceutical Botany
Faculty of Pharmaceutical Sciences
Chulalongkorn University
Bangkok, 10330, Thailand
Tel: +6622188281
FAX: +6622545195
Email: suchada.su@chula.ac.th

Supplementary information

Supplementary Data S1. Alignment of *rbc*L barcode region of *C. cinereum* and *E. sonchifolia*

Supplementary Data S2. Alignment of *mat*K gene from *C. cinereum* and *E. sonchifolia*.

Supplementary Data S3. Alignment of ITS barcode region of *C. cinereum* and *E. sonchifolia*.

Supplementary Data S4. Alignment of *psb*A-*trn*H barcode region of *C. cinereum* and *E. sonchifolia*

Supplementary Figure S1. Structures of four triterpenoid compounds from a distinct band of *C. cinereum* ethanolic extract. (A) *β*-amyrin, (B) taraxasterol, (C) lupeol, and (D) betulin.

Supplementary Table S1. Primers used for DNA barcode generation and sequencing.

Supplementary Data S1. Alignment of *rbc*L barcode region of *C. cinereum* and *E. sonchifolia*

```
                ....|....| ....|....| ....|....| ....|....| ....|....|
                     5         15         25         35         45
LC503541_C      ATGTCACCAC AAACAGAGAC TAAAGCAAGT GTTGGATTCA AAGCTGGTGT
LC503549_C      ATGTCACCAC AAACAGAGAC TAAAGCAAGT GTTGGATTCA AAGCTGGTGT
LC503557_C      ATGTCACCAC AAACAGAGAC TAAAGCAAGT GTTGGATTCA AAGCTGGTGT
LC503565_C      ATGTCACCAC AAACAGAGAC TAAAGCAAGT GTTGGATTCA AAGCTGGTGT
LC503573_C      ATGTCACCAC AAACAGAGAC TAAAGCAAGT GTTGGATTCA AAGCTGGTGT
LC503581_C      ATGTCACCAC AAACAGAGAC TAAAGCAAGT GTTGGATTCA AAGCTGGTGT
LC503589_C      ATGTCACCAC AAACAGAGAC TAAAGCAAGT GTTGGATTCA AAGCTGGTGT
LC503545_E      ATGTCACCAC AAACAGAGAC TAAAGCGAGT GTTGGATTCA AAGCTGGTGT
LC503553_E      ATGTCACCAC AAACAGAGAC TAAAGCGAGT GTTGGATTCA AAGCTGGTGT
LC503561_E      ATGTCACCAC AAACAGAGAC TAAAGCGAGT GTTGGATTCA AAGCTGGTGT
LC503569_E      ATGTCACCAC AAACAGAGAC TAAAGCGAGT GTTGGATTCA AAGCTGGTGT
LC503577_E      ATGTCACCAC AAACAGAGAC TAAAGCGAGT GTTGGATTCA AAGCTGGTGT
LC503585_E      ATGTCACCAC AAACAGAGAC TAAAGCGAGT GTTGGATTCA AAGCTGGTGT
LC503593_E      ATGTCACCAC AAACAGAGAC TAAAGCGAGT GTTGGATTCA AAGCTGGTGT

                ....|....| ....|....| ....|....| ....|....| ....|....|
                     55         65         75         85         95
LC503541_C      TAAAGATTAT AAATTGACTT ATTATACTCC TGAATATAAA ACCAAGGATA
LC503549_C      TAAAGATTAT AAATTGACTT ATTATACTCC TGAATATAAA ACCAAGGATA
LC503557_C      TAAAGATTAT AAATTGACTT ATTATACTCC TGAATATAAA ACCAAGGATA
LC503565_C      TAAAGATTAT AAATTGACTT ATTATACTCC TGAATATAAA ACCAAGGATA
LC503573_C      TAAAGATTAT AAATTGACTT ATTATACTCC TGAATATAAA ACCAAGGATA
LC503581_C      TAAAGATTAT AAATTGACTT ATTATACTCC TGAATATAAA ACCAAGGATA
LC503589_C      TAAAGATTAT AAATTGACTT ATTATACTCC TGAATATAAA ACCAAGGATA
LC503545_E      TAAAGATTAT AAATTGAATT ATTATACTCC TGAGTATGAA ACCAAGGATA
LC503553_E      TAAAGATTAT AAATTGAATT ATTATACTCC TGAGTATGAA ACCAAGGATA
LC503561_E      TAAAGATTAT AAATTGAATT ATTATACTCC TGAGTATGAA ACCAAGGATA
LC503569_E      TAAAGATTAT AAATTGAATT ATTATACTCC TGAGTATGAA ACCAAGGATA
LC503577_E      TAAAGATTAT AAATTGAATT ATTATACTCC TGAGTATGAA ACCAAGGATA
LC503585_E      TAAAGATTAT AAATTGAATT ATTATACTCC TGAGTATGAA ACCAAGGATA
LC503593_E      TAAAGATTAT AAATTGAATT ATTATACTCC TGAGTATGAA ACCAAGGATA

                ....|....| ....|....| ....|....| ....|....| ....|....|
                    105        115        125        135        145
LC503541_C      CTGATATCTT GGCAGCATTT CGAGTAACTC CTCAACCTGG AGTTCCGCCT
LC503549_C      CTGATATCTT GGCAGCATTT CGAGTAACTC CTCAACCTGG AGTTCCGCCT
LC503557_C      CTGATATCTT GGCAGCATTT CGAGTAACTC CTCAACCTGG AGTTCCGCCT
LC503565_C      CTGATATCTT GGCAGCATTT CGAGTAACTC CTCAACCTGG AGTTCCGCCT
LC503573_C      CTGATATCTT GGCAGCATTT CGAGTAACTC CTCAACCTGG AGTTCCGCCT
LC503581_C      CTGATATCTT GGCAGCATTT CGAGTAACTC CTCAACCTGG AGTTCCGCCT
LC503589_C      CTGATATCTT GGCAGCATTT CGAGTAACTC CTCAACCTGG AGTTCCGCCT
LC503545_E      CTGATATCTT GGCAGCATTT CGAGTAACTC CTCAACCAGG AGTTCCGCCT
LC503553_E      CTGATATCTT GGCAGCATTT CGAGTAACTC CTCAACCAGG AGTTCCGCCT
LC503561_E      CTGATATCTT GGCAGCATTT CGAGTAACTC CTCAACCAGG AGTTCCGCCT
LC503569_E      CTGATATCTT GGCAGCATTT CGAGTAACTC CTCAACCAGG AGTTCCGCCT
LC503577_E      CTGATATCTT GGCAGCATTT CGAGTAACTC CTCAACCAGG AGTTCCGCCT
LC503585_E      CTGATATCTT GGCAGCATTT CGAGTAACTC CTCAACCAGG AGTTCCGCCT
LC503593_E      CTGATATCTT GGCAGCATTT CGAGTAACTC CTCAACCAGG AGTTCCGCCT

                ....|....| ....|....| ....|....| ....|....| ....|....|
                    155        165        175        185        195
LC503541_C      GAAGAAGCAG GGGCCGCAGT AGCTGCCGAA TCTTCTACTG GTACATGGAC
LC503549_C      GAAGAAGCAG GGGCCGCAGT AGCTGCCGAA TCTTCTACTG GTACATGGAC
LC503557_C      GAAGAAGCAG GGGCCGCAGT AGCTGCCGAA TCTTCTACTG GTACATGGAC
LC503565_C      GAAGAAGCAG GGGCCGCAGT AGCTGCCGAA TCTTCTACTG GTACATGGAC
LC503573_C      GAAGAAGCAG GGGCCGCAGT AGCTGCCGAA TCTTCTACTG GTACATGGAC
```

```
LC503581_C   GAAGAAGCAG GGGCCGCAGT AGCTGCCGAA TCTTCTACTG GTACATGGAC
LC503589_C   GAAGAAGCAG GGGCCGCAGT AGCTGCCGAA TCTTCTACTG GTACATGGAC
LC503545_E   GAAGAAGCAG GGGCCGCAGT AGCTGCCGAA TCTTCTACTG GTACATGGAC
LC503553_E   GAAGAAGCAG GGGCCGCAGT AGCTGCCGAA TCTTCTACTG GTACATGGAC
LC503561_E   GAAGAAGCAG GGGCCGCAGT AGCTGCCGAA TCTTCTACTG GTACATGGAC
LC503569_E   GAAGAAGCAG GGGCCGCAGT AGCTGCCGAA TCTTCTACTG GTACATGGAC
LC503577_E   GAAGAAGCAG GGGCCGCAGT AGCTGCCGAA TCTTCTACTG GTACATGGAC
LC503585_E   GAAGAAGCAG GGGCCGCAGT AGCTGCCGAA TCTTCTACTG GTACATGGAC
LC503593_E   GAAGAAGCAG GGGCCGCAGT AGCTGCCGAA TCTTCTACTG GTACATGGAC

             ....|....| ....|....| ....|....| ....|....| ....|....|
                  205        215        225        235        245
LC503541_C   AACTGTGTGG ACCGATGGAC TTACGAGCCT TGATCGTTAC AAAGGGCGAT
LC503549_C   AACTGTGTGG ACCGATGGAC TTACGAGCCT TGATCGTTAC AAAGGGCGAT
LC503557_C   AACTGTGTGG ACCGATGGAC TTACGAGCCT TGATCGTTAC AAAGGGCGAT
LC503565_C   AACTGTGTGG ACCGATGGAC TTACGAGCCT TGATCGTTAC AAAGGGCGAT
LC503573_C   AACTGTGTGG ACCGATGGAC TTACGAGCCT TGATCGTTAC AAAGGGCGAT
LC503581_C   AACTGTGTGG ACCGATGGAC TTACGAGCCT TGATCGTTAC AAAGGGCGAT
LC503589_C   AACTGTGTGG ACCGATGGAC TTACGAGCCT TGATCGTTAC AAAGGGCGAT
LC503545_E   AACTGTGTGG ACCGATGGAC TTACGAGCCT TGATCGTTAC AAAGGGCGAT
LC503553_E   AACTGTGTGG ACCGATGGAC TTACGAGCCT TGATCGTTAC AAAGGGCGAT
LC503561_E   AACTGTGTGG ACCGATGGAC TTACGAGCCT TGATCGTTAC AAAGGGCGAT
LC503569_E   AACTGTGTGG ACCGATGGAC TTACGAGCCT TGATCGTTAC AAAGGGCGAT
LC503577_E   AACTGTGTGG ACCGATGGAC TTACGAGCCT TGATCGTTAC AAAGGGCGAT
LC503585_E   AACTGTGTGG ACCGATGGAC TTACGAGCCT TGATCGTTAC AAAGGGCGAT
LC503593_E   AACTGTGTGG ACCGATGGAC TTACGAGCCT TGATCGTTAC AAAGGGCGAT

             ....|....| ....|....| ....|....| ....|....| ....|....|
                  255        265        275        285        295
LC503541_C   GCTATGGAAT CGAGCCTGTT CCTGGAGAAG AAAGTCAATT TATTGCTTAT
LC503549_C   GCTATGGAAT CGAGCCTGTT CCTGGAGAAG AAAGTCAATT TATTGCTTAT
LC503557_C   GCTATGGAAT CGAGCCTGTT CCTGGAGAAG AAAGTCAATT TATTGCTTAT
LC503565_C   GCTATGGAAT CGAGCCTGTT CCTGGAGAAG AAAGTCAATT TATTGCTTAT
LC503573_C   GCTATGGAAT CGAGCCTGTT CCTGGAGAAG AAAGTCAATT TATTGCTTAT
LC503581_C   GCTATGGAAT CGAGCCTGTT CCTGGAGAAG AAAGTCAATT TATTGCTTAT
LC503589_C   GCTATGGAAT CGAGCCTGTT CCTGGAGAAG AAAGTCAATT TATTGCTTAT
LC503545_E   GCTATCGAAT CGAGCCTGTT CTTGGAGAAG AAAATCAATA TATTGCTTAT
LC503553_E   GCTATCGAAT CGAGCCTGTT CTTGGAGAAG AAAATCAATA TATTGCTTAT
LC503561_E   GCTATCGAAT CGAGCCTGTT CTTGGAGAAG AAAATCAATA TATTGCTTAT
LC503569_E   GCTATCGAAT CGAGCCTGTT CTTGGAGAAG AAAATCAATA TATTGCTTAT
LC503577_E   GCTATCGAAT CGAGCCTGTT CTTGGAGAAG AAAATCAATA TATTGCTTAT
LC503585_E   GCTATCGAAT CGAGCCTGTT CTTGGAGAAG AAAATCAATA TATTGCTTAT
LC503593_E   GCTATCGAAT CGAGCCTGTT CTTGGAGAAG AAAATCAATA TATTGCTTAT

             ....|....| ....|....| ....|....| ....|....| ....|....|
                  305        315        325        335        345
LC503541_C   GTAGCTTACC CATTAGACCT TTTTGAAGAA GGTTCTGTTA CTAACATGTT
LC503549_C   GTAGCTTACC CATTAGACCT TTTTGAAGAA GGTTCTGTTA CTAACATGTT
LC503557_C   GTAGCTTACC CATTAGACCT TTTTGAAGAA GGTTCTGTTA CTAACATGTT
LC503565_C   GTAGCTTACC CATTAGACCT TTTTGAAGAA GGTTCTGTTA CTAACATGTT
LC503573_C   GTAGCTTACC CATTAGACCT TTTTGAAGAA GGTTCTGTTA CTAACATGTT
LC503581_C   GTAGCTTACC CATTAGACCT TTTTGAAGAA GGTTCTGTTA CTAACATGTT
LC503589_C   GTAGCTTACC CATTAGACCT TTTTGAAGAA GGTTCTGTTA CTAACATGTT
LC503545_E   GTAGCTTACC CATTAGACCT TTTTGAAGAA GGTTCTGTTA CTAACATGTT
LC503553_E   GTAGCTTACC CATTAGACCT TTTTGAAGAA GGTTCTGTTA CTAACATGTT
LC503561_E   GTAGCTTACC CATTAGACCT TTTTGAAGAA GGTTCTGTTA CTAACATGTT
LC503569_E   GTAGCTTACC CATTAGACCT TTTTGAAGAA GGTTCTGTTA CTAACATGTT
LC503577_E   GTAGCTTACC CATTAGACCT TTTTGAAGAA GGTTCTGTTA CTAACATGTT
LC503585_E   GTAGCTTACC CATTAGACCT TTTTGAAGAA GGTTCTGTTA CTAACATGTT
LC503593_E   GTAGCTTACC CATTAGACCT TTTTGAAGAA GGTTCTGTTA CTAACATGTT
```

```
               ....|....| ....|....| ....|....| ....|....| ....|....|
                   355        365        375        385        395
LC503541_C     TACTTCCATT GTAGGTAATG TATTTGGGTT CAAAGCCCTG CGTGCTCTAC
LC503549_C     TACTTCCATT GTAGGTAATG TATTTGGGTT CAAAGCCCTG CGTGCTCTAC
LC503557_C     TACTTCCATT GTAGGTAATG TATTTGGGTT CAAAGCCCTG CGTGCTCTAC
LC503565_C     TACTTCCATT GTAGGTAATG TATTTGGGTT CAAAGCCCTG CGTGCTCTAC
LC503573_C     TACTTCCATT GTAGGTAATG TATTTGGGTT CAAAGCCCTG CGTGCTCTAC
LC503581_C     TACTTCCATT GTAGGTAATG TATTTGGGTT CAAAGCCCTG CGTGCTCTAC
LC503589_C     TACTTCCATT GTAGGTAATG TATTTGGGTT CAAAGCCCTG CGTGCTCTAC
LC503545_E     TACTTCCATT GTAGGTAATG TATTTGGGTT CAAAGCCCTG CGTGCTCTAC
LC503553_E     TACTTCCATT GTAGGTAATG TATTTGGGTT CAAAGCCCTG CGTGCTCTAC
LC503561_E     TACTTCCATT GTAGGTAATG TATTTGGGTT CAAAGCCCTG CGTGCTCTAC
LC503569_E     TACTTCCATT GTAGGTAATG TATTTGGGTT CAAAGCCCTG CGTGCTCTAC
LC503577_E     TACTTCCATT GTAGGTAATG TATTTGGGTT CAAAGCCCTG CGTGCTCTAC
LC503585_E     TACTTCCATT GTAGGTAATG TATTTGGGTT CAAAGCCCTG CGTGCTCTAC
LC503593_E     TACTTCCATT GTAGGTAATG TATTTGGGTT CAAAGCCCTG CGTGCTCTAC

               ....|....| ....|....| ....|....| ....|....| ....|....|
                   405        415        425        435        445
LC503541_C     GTCTGGAAGA TTTGCGAATC CCTATTTCGT ATGTTAAAAC TTTCCAAGGT
LC503549_C     GTCTGGAAGA TTTGCGAATC CCTATTTCGT ATGTTAAAAC TTTCCAAGGT
LC503557_C     GTCTGGAAGA TTTGCGAATC CCTATTTCGT ATGTTAAAAC TTTCCAAGGT
LC503565_C     GTCTGGAAGA TTTGCGAATC CCTATTTCGT ATGTTAAAAC TTTCCAAGGT
LC503573_C     GTCTGGAAGA TTTGCGAATC CCTATTTCGT ATGTTAAAAC TTTCCAAGGT
LC503581_C     GTCTGGAAGA TTTGCGAATC CCTATTTCGT ATGTTAAAAC TTTCCAAGGT
LC503589_C     GTCTGGAAGA TTTGCGAATC CCTATTTCGT ATGTTAAAAC TTTCCAAGGT
LC503545_E     GTCTGGAAGA TTTGCGAATC CCTACTGCGT ATATTAAAAC TTTCCAAGGT
LC503553_E     GTCTGGAAGA TTTGCGAATC CCTACTGCGT ATATTAAAAC TTTCCAAGGT
LC503561_E     GTCTGGAAGA TTTGCGAATC CCTACTGCGT ATATTAAAAC TTTCCAAGGT
LC503569_E     GTCTGGAAGA TTTGCGAATC CCTACTGCGT ATATTAAAAC TTTCCAAGGT
LC503577_E     GTCTGGAAGA TTTGCGAATC CCTACTGCGT ATATTAAAAC TTTCCAAGGT
LC503585_E     GTCTGGAAGA TTTGCGAATC CCTACTGCGT ATATTAAAAC TTTCCAAGGT
LC503593_E     GTCTGGAAGA TTTGCGAATC CCTACTGCGT ATATTAAAAC TTTCCAAGGT

               ....|....| ....|....| ....|....| ....|....| ....|....|
                   455        465        475        485        495
LC503541_C     CCGCCTCACG GCATCCAAGT TGAGAGAGAT AAATTGAACA AGTATGGTCG
LC503549_C     CCGCCTCACG GCATCCAAGT TGAGAGAGAT AAATTGAACA AGTATGGTCG
LC503557_C     CCGCCTCACG GCATCCAAGT TGAGAGAGAT AAATTGAACA AGTATGGTCG
LC503565_C     CCGCCTCACG GCATCCAAGT TGAGAGAGAT AAATTGAACA AGTATGGTCG
LC503573_C     CCGCCTCACG GCATCCAAGT TGAGAGAGAT AAATTGAACA AGTATGGTCG
LC503581_C     CCGCCTCACG GCATCCAAGT TGAGAGAGAT AAATTGAACA AGTATGGTCG
LC503589_C     CCGCCTCACG GCATCCAAGT TGAGAGAGAT AAATTGAACA AGTATGGTCG
LC503545_E     CCGCCTCACG GCATCCAAGT TGAAAGAGAT AAATTGAACA AGTATGGTCG
LC503553_E     CCGCCTCACG GCATCCAAGT TGAAAGAGAT AAATTGAACA AGTATGGTCG
LC503561_E     CCGCCTCACG GCATCCAAGT TGAAAGAGAT AAATTGAACA AGTATGGTCG
LC503569_E     CCGCCTCACG GCATCCAAGT TGAAAGAGAT AAATTGAACA AGTATGGTCG
LC503577_E     CCGCCTCACG GCATCCAAGT TGAAAGAGAT AAATTGAACA AGTATGGTCG
LC503585_E     CCGCCTCACG GCATCCAAGT TGAAAGAGAT AAATTGAACA AGTATGGTCG
LC503593_E     CCGCCTCACG GCATCCAAGT TGAAAGAGAT AAATTGAACA AGTATGGTCG

               ....|....| ....|....| ....|....| ....|....| ....|....|
                   505        515        525        535        545
LC503541_C     TCCCCTGTTG GGATGTACTA TTAAACCTAA ATTGGGGTTA TCCGCTAAAA
LC503549_C     TCCCCTGTTG GGATGTACTA TTAAACCTAA ATTGGGGTTA TCCGCTAAAA
LC503557_C     TCCCCTGTTG GGATGTACTA TTAAACCTAA ATTGGGGTTA TCCGCTAAAA
LC503565_C     TCCCCTGTTG GGATGTACTA TTAAACCTAA ATTGGGGTTA TCCGCTAAAA
LC503573_C     TCCCCTGTTG GGATGTACTA TTAAACCTAA ATTGGGGTTA TCCGCTAAAA
LC503581_C     TCCCCTGTTG GGATGTACTA TTAAACCTAA ATTGGGGTTA TCCGCTAAAA
LC503589_C     TCCCCTGTTG GGATGTACTA TTAAACCTAA ATTGGGGTTA TCCGCTAAAA
LC503545_E     TCCTCTATTG GGATGTACTA TTAAACCTAA ATTGGGGCTA TCCGCTAAAA
```

```
LC503553_E   TCCTCTATTG GGATGTACTA TTAAACCTAA ATTGGGGCTA TCCGCTAAAA
LC503561_E   TCCTCTATTG GGATGTACTA TTAAACCTAA ATTGGGGCTA TCCGCTAAAA
LC503569_E   TCCTCTATTG GGATGTACTA TTAAACCTAA ATTGGGGCTA TCCGCTAAAA
LC503577_E   TCCTCTATTG GGATGTACTA TTAAACCTAA ATTGGGGCTA TCCGCTAAAA
LC503585_E   TCCTCTATTG GGATGTACTA TTAAACCTAA ATTGGGGCTA TCCGCTAAAA
LC503593_E   TCCTCTATTG GGATGTACTA TTAAACCTAA ATTGGGGCTA TCCGCTAAAA

             ....|....| ....|....| ....|....| ....|....| ....|....|
                   555        565        575        585        595
LC503541_C   ACTACGGTAG AGCTGTTTAT GAATGTCTTC GTGGTGGCCT TGATTTTACT
LC503549_C   ACTACGGTAG AGCTGTTTAT GAATGTCTTC GTGGTGGCCT TGATTTTACT
LC503557_C   ACTACGGTAG AGCTGTTTAT GAATGTCTTC GTGGTGGCCT TGATTTTACT
LC503565_C   ACTACGGTAG AGCTGTTTAT GAATGTCTTC GTGGTGGCCT TGATTTTACT
LC503573_C   ACTACGGTAG AGCTGTTTAT GAATGTCTTC GTGGTGGCCT TGATTTTACT
LC503581_C   ACTACGGTAG AGCTGTTTAT GAATGTCTTC GTGGTGGCCT TGATTTTACT
LC503589_C   ACTACGGTAG AGCTGTTTAT GAATGTCTTC GTGGTGGCCT TGATTTTACT
LC503545_E   ACTACGGTAG AGCTGTTTAT GAGTGTCTTC GTGGTGGCCT TGATTTTACT
LC503553_E   ACTACGGTAG AGCTGTTTAT GAGTGTCTTC GTGGTGGCCT TGATTTTACT
LC503561_E   ACTACGGTAG AGCTGTTTAT GAGTGTCTTC GTGGTGGCCT TGATTTTACT
LC503569_E   ACTACGGTAG AGCTGTTTAT GAGTGTCTTC GTGGTGGCCT TGATTTTACT
LC503577_E   ACTACGGTAG AGCTGTTTAT GAGTGTCTTC GTGGTGGCCT TGATTTTACT
LC503585_E   ACTACGGTAG AGCTGTTTAT GAGTGTCTTC GTGGTGGCCT TGATTTTACT
LC503593_E   ACTACGGTAG AGCTGTTTAT GAGTGTCTTC GTGGTGGCCT TGATTTTACT

             ....|....| ....|....| ....|....| ....|....| ....|....|
                   605        615        625        635        645
LC503541_C   AAAGATGATG AGAACGTGAA CTCCCAACCA TTTATGCGTT GGAGAGACCG
LC503549_C   AAAGATGATG AGAACGTGAA CTCCCAACCA TTTATGCGTT GGAGAGACCG
LC503557_C   AAAGATGATG AGAACGTGAA CTCCCAACCA TTTATGCGTT GGAGAGACCG
LC503565_C   AAAGATGATG AGAACGTGAA CTCCCAACCA TTTATGCGTT GGAGAGACCG
LC503573_C   AAAGATGATG AGAACGTGAA CTCCCAACCA TTTATGCGTT GGAGAGACCG
LC503581_C   AAAGATGATG AGAACGTGAA CTCCCAACCA TTTATGCGTT GGAGAGACCG
LC503589_C   AAAGATGATG AGAACGTGAA CTCCCAACCA TTTATGCGTT GGAGAGACCG
LC503545_E   AAAGATGATG GAAACGTGAA CTCCCAACCA TTTATGCGTT GGAGAGACCG
LC503553_E   AAAGATGATG GAAACGTGAA CTCCCAACCA TTTATGCGTT GGAGAGACCG
LC503561_E   AAAGATGATG GAAACGTGAA CTCCCAACCA TTTATGCGTT GGAGAGACCG
LC503569_E   AAAGATGATG GAAACGTGAA CTCCCAACCA TTTATGCGTT GGAGAGACCG
LC503577_E   AAAGATGATG GAAACGTGAA CTCCCAACCA TTTATGCGTT GGAGAGACCG
LC503585_E   AAAGATGATG GAAACGTGAA CTCCCAACCA TTTATGCGTT GGAGAGACCG
LC503593_E   AAAGATGATG GAAACGTGAA CTCCCAACCA TTTATGCGTT GGAGAGACCG

             ....|....| ....|....| ....|....| ....|....| ....|....|
                   655        665        675        685        695
LC503541_C   TTTCTGCTTT TGTGCCGAAG CTCTTTTTAA AGCACAAGCT GAAACAGGTG
LC503549_C   TTTCTGCTTT TGTGCCGAAG CTCTTTTTAA AGCACAAGCT GAAACAGGTG
LC503557_C   TTTCTGCTTT TGTGCCGAAG CTCTTTTTAA AGCACAAGCT GAAACAGGTG
LC503565_C   TTTCTGCTTT TGTGCCGAAG CTCTTTTTAA AGCACAAGCT GAAACAGGTG
LC503573_C   TTTCTGCTTT TGTGCCGAAG CTCTTTTTAA AGCACAAGCT GAAACAGGTG
LC503581_C   TTTCTGCTTT TGTGCCGAAG CTCTTTTTAA AGCACAAGCT GAAACAGGTG
LC503589_C   TTTCTGCTTT TGTGCCGAAG CTCTTTTTAA AGCACAAGCT GAAACAGGTG
LC503545_E   TTTCTTATTT TGTGCCGAAG CTATTTATAA ACCACAAGCT GAAACAGGTG
LC503553_E   TTTCTTATTT TGTGCCGAAG CTATTTATAA ACCACAAGCT GAAACAGGTG
LC503561_E   TTTCTTATTT TGTGCCGAAG CTATTTATAA ACCACAAGCT GAAACAGGTG
LC503569_E   TTTCTTATTT TGTGCCGAAG CTATTTATAA ACCACAAGCT GAAACAGGTG
LC503577_E   TTTCTTATTT TGTGCCGAAG CTATTTATAA ACCACAAGCT GAAACAGGTG
LC503585_E   TTTCTTATTT TGTGCCGAAG CTATTTATAA ACCACAAGCT GAAACAGGTG
LC503593_E   TTTCTTATTT TGTGCCGAAG CTATTTATAA ACCACAAGCT GAAACAGGTG

             ....|....| ....|....| ....|....| ....|....| ....|....|
                   705        715        725        735        745
LC503541_C   AAATCAAAGG GCATTACTTG AATGCTACTG CGGGTACAGT CGACGAAATG
```

```
LC503549_C   AAATCAAAGG GCATTACTTG AATGCTACTG CGGGTACAGT CGACGAAATG
LC503557_C   AAATCAAAGG GCATTACTTG AATGCTACTG CGGGTACAGT CGACGAAATG
LC503565_C   AAATCAAAGG GCATTACTTG AATGCTACTG CGGGTACAGT CGACGAAATG
LC503573_C   AAATCAAAGG GCATTACTTG AATGCTACTG CGGGTACAGT CGACGAAATG
LC503581_C   AAATCAAAGG GCATTACTTG AATGCTACTG CGGGTACAGT CGACGAAATG
LC503589_C   AAATCAAAGG GCATTACTTG AATGCTACTG CGGGTACAGT CGACGAAATG
LC503545_E   AAATCAAAGG GCATTACTTG AATGCTACTG CGGGTACATG CGAAGAAATG
LC503553_E   AAATCAAAGG GCATTACTTG AATGCTACTG CGGGTACATG CGAAGAAATG
LC503561_E   AAATCAAAGG GCATTACTTG AATGCTACTG CGGGTACATG CGAAGAAATG
LC503569_E   AAATCAAAGG GCATTACTTG AATGCTACTG CGGGTACATG CGAAGAAATG
LC503577_E   AAATCAAAGG GCATTACTTG AATGCTACTG CGGGTACATG CGAAGAAATG
LC503585_E   AAATCAAAGG GCATTACTTG AATGCTACTG CGGGTACATG CGAAGAAATG
LC503593_E   AAATCAAAGG GCATTACTTG AATGCTACTG CGGGTACATG CGAAGAAATG

             ....|....| ....|....| ....|....| ....|....| ....|....|
                   755        765        775        785        795
LC503541_C   ATGAAAAGGG CTATATTTGC CAGAGAATTG GGAGTTCCTA TCGTAATGCA
LC503549_C   ATGAAAAGGG CTATATTTGC CAGAGAATTG GGAGTTCCTA TCGTAATGCA
LC503557_C   ATGAAAAGGG CTATATTTGC CAGAGAATTG GGAGTTCCTA TCGTAATGCA
LC503565_C   ATGAAAAGGG CTATATTTGC CAGAGAATTG GGAGTTCCTA TCGTAATGCA
LC503573_C   ATGAAAAGGG CTATATTTGC CAGAGAATTG GGAGTTCCTA TCGTAATGCA
LC503581_C   ATGAAAAGGG CTATATTTGC CAGAGAATTG GGAGTTCCTA TCGTAATGCA
LC503589_C   ATGAAAAGGG CTATATTTGC CAGAGAATTG GGAGTTCCTA TCGTAATGCA
LC503545_E   ATGAAAAGGG CTATATTTGC CAGAGAATTG GGAGTTCCTA TCGTAATGCA
LC503553_E   ATGAAAAGGG CTATATTTGC CAGAGAATTG GGAGTTCCTA TCGTAATGCA
LC503561_E   ATGAAAAGGG CTATATTTGC CAGAGAATTG GGAGTTCCTA TCGTAATGCA
LC503569_E   ATGAAAAGGG CTATATTTGC CAGAGAATTG GGAGTTCCTA TCGTAATGCA
LC503577_E   ATGAAAAGGG CTATATTTGC CAGAGAATTG GGAGTTCCTA TCGTAATGCA
LC503585_E   ATGAAAAGGG CTATATTTGC CAGAGAATTG GGAGTTCCTA TCGTAATGCA
LC503593_E   ATGAAAAGGG CTATATTTGC CAGAGAATTG GGAGTTCCTA TCGTAATGCA

             ....|....| ....|....| ....|....| ....|....| ....|....|
                   805        815        825        835        845
LC503541_C   TGACTACCTA ACAGGGGGAT TCACTGCAAA TACTACCTTG GCTCAATATT
LC503549_C   TGACTACCTA ACAGGGGGAT TCACTGCAAA TACTACCTTG GCTCAATATT
LC503557_C   TGACTACCTA ACAGGGGGAT TCACTGCAAA TACTACCTTG GCTCAATATT
LC503565_C   TGACTACCTA ACAGGGGGAT TCACTGCAAA TACTACCTTG GCTCAATATT
LC503573_C   TGACTACCTA ACAGGGGGAT TCACTGCAAA TACTACCTTG GCTCAATATT
LC503581_C   TGACTACCTA ACAGGGGGAT TCACTGCAAA TACTACCTTG GCTCAATATT
LC503589_C   TGACTACCTA ACAGGGGGAT TCACTGCAAA TACTACCTTG GCTCAATATT
LC503545_E   TGACTACCTA ACAGGGGGAT TCACTGCAAA TACTAGCTTG GCTCATTATT
LC503553_E   TGACTACCTA ACAGGGGGAT TCACTGCAAA TACTAGCTTG GCTCATTATT
LC503561_E   TGACTACCTA ACAGGGGGAT TCACTGCAAA TACTAGCTTG GCTCATTATT
LC503569_E   TGACTACCTA ACAGGGGGAT TCACTGCAAA TACTAGCTTG GCTCATTATT
LC503577_E   TGACTACCTA ACAGGGGGAT TCACTGCAAA TACTAGCTTG GCTCATTATT
LC503585_E   TGACTACCTA ACAGGGGGAT TCACTGCAAA TACTAGCTTG GCTCATTATT
LC503593_E   TGACTACCTA ACAGGGGGAT TCACTGCAAA TACTAGCTTG GCTCATTATT

             ....|....| ....|....| ....|....| ....|....| ....|....|
                   855        865        875        885        895
LC503541_C   GCCGAGATAA TGGCCTACTT CTTCACATCC ACCGCGCAAT GCATGCAGTT
LC503549_C   GCCGAGATAA TGGCCTACTT CTTCACATCC ACCGCGCAAT GCATGCAGTT
LC503557_C   GCCGAGATAA TGGCCTACTT CTTCACATCC ACCGCGCAAT GCATGCAGTT
LC503565_C   GCCGAGATAA TGGCCTACTT CTTCACATCC ACCGCGCAAT GCATGCAGTT
LC503573_C   GCCGAGATAA TGGCCTACTT CTTCACATCC ACCGCGCAAT GCATGCAGTT
LC503581_C   GCCGAGATAA TGGCCTACTT CTTCACATCC ACCGCGCAAT GCATGCAGTT
LC503589_C   GCCGAGATAA TGGCCTACTT CTTCACATCC ACCGCGCAAT GCATGCAGTT
LC503545_E   GCCGAGATAA TGGCCTACTT CTTCACATCC ACCGCGCAAT GCATGCAGTT
LC503553_E   GCCGAGATAA TGGCCTACTT CTTCACATCC ACCGCGCAAT GCATGCAGTT
LC503561_E   GCCGAGATAA TGGCCTACTT CTTCACATCC ACCGCGCAAT GCATGCAGTT
LC503569_E   GCCGAGATAA TGGCCTACTT CTTCACATCC ACCGCGCAAT GCATGCAGTT
```

```
LC503577_E   GCCGAGATAA TGGCCTACTT CTTCACATCC ACCGCGCAAT GCATGCAGTT
LC503585_E   GCCGAGATAA TGGCCTACTT CTTCACATCC ACCGCGCAAT GCATGCAGTT
LC503593_E   GCCGAGATAA TGGCCTACTT CTTCACATCC ACCGCGCAAT GCATGCAGTT

             ....|....| ....|....| ....|....| ....|....| ....|....|
                   905        915        925        935        945
LC503541_C   ATTGATAGAC AGAAGAATCA TGGTATGCAC TTTCGTGTAC TAGCTAAAGC
LC503549_C   ATTGATAGAC AGAAGAATCA TGGTATGCAC TTTCGTGTAC TAGCTAAAGC
LC503557_C   ATTGATAGAC AGAAGAATCA TGGTATGCAC TTTCGTGTAC TAGCTAAAGC
LC503565_C   ATTGATAGAC AGAAGAATCA TGGTATGCAC TTTCGTGTAC TAGCTAAAGC
LC503573_C   ATTGATAGAC AGAAGAATCA TGGTATGCAC TTTCGTGTAC TAGCTAAAGC
LC503581_C   ATTGATAGAC AGAAGAATCA TGGTATGCAC TTTCGTGTAC TAGCTAAAGC
LC503589_C   ATTGATAGAC AGAAGAATCA TGGTATGCAC TTTCGTGTAC TAGCTAAAGC
LC503545_E   ATTGATAGAC AGAAGAATCA TGGTATGCAC TTTCGTGTAC TAGCTAAAGC
LC503553_E   ATTGATAGAC AGAAGAATCA TGGTATGCAC TTTCGTGTAC TAGCTAAAGC
LC503561_E   ATTGATAGAC AGAAGAATCA TGGTATGCAC TTTCGTGTAC TAGCTAAAGC
LC503569_E   ATTGATAGAC AGAAGAATCA TGGTATGCAC TTTCGTGTAC TAGCTAAAGC
LC503577_E   ATTGATAGAC AGAAGAATCA TGGTATGCAC TTTCGTGTAC TAGCTAAAGC
LC503585_E   ATTGATAGAC AGAAGAATCA TGGTATGCAC TTTCGTGTAC TAGCTAAAGC
LC503593_E   ATTGATAGAC AGAAGAATCA TGGTATGCAC TTTCGTGTAC TAGCTAAAGC

             ....|....| ....|....| ....|....| ....|....| ....|....|
                   955        965        975        985        995
LC503541_C   ATTACGTATG TCTGGTGGAG ATCATATTCA TGCCGGTACC GTAGTAGGTA
LC503549_C   ATTACGTATG TCTGGTGGAG ATCATATTCA TGCCGGTACC GTAGTAGGTA
LC503557_C   ATTACGTATG TCTGGTGGAG ATCATATTCA TGCCGGTACC GTAGTAGGTA
LC503565_C   ATTACGTATG TCTGGTGGAG ATCATATTCA TGCCGGTACC GTAGTAGGTA
LC503573_C   ATTACGTATG TCTGGTGGAG ATCATATTCA TGCCGGTACC GTAGTAGGTA
LC503581_C   ATTACGTATG TCTGGTGGAG ATCATATTCA TGCCGGTACC GTAGTAGGTA
LC503589_C   ATTACGTATG TCTGGTGGAG ATCATATTCA TGCCGGTACC GTAGTAGGTA
LC503545_E   GTTACGTATG TCTGGTGGAG ACCATATTCA TGCCGGTACC GTAGTAGGTA
LC503553_E   GTTACGTATG TCTGGTGGAG ACCATATTCA TGCCGGTACC GTAGTAGGTA
LC503561_E   GTTACGTATG TCTGGTGGAG ACCATATTCA TGCCGGTACC GTAGTAGGTA
LC503569_E   GTTACGTATG TCTGGTGGAG ACCATATTCA TGCCGGTACC GTAGTAGGTA
LC503577_E   GTTACGTATG TCTGGTGGAG ACCATATTCA TGCCGGTACC GTAGTAGGTA
LC503585_E   GTTACGTATG TCTGGTGGAG ACCATATTCA TGCCGGTACC GTAGTAGGTA
LC503593_E   GTTACGTATG TCTGGTGGAG ACCATATTCA TGCCGGTACC GTAGTAGGTA

             ....|....| ....|....| ....|....| ....|....| ....|....|
                  1005       1015       1025       1035       1045
LC503541_C   AACTTGAAGG GGAAAGAGAA ATCACTTTGG GCTTTGTTGA TTTACTGCGT
LC503549_C   AACTTGAAGG GGAAAGAGAA ATCACTTTGG GCTTTGTTGA TTTACTGCGT
LC503557_C   AACTTGAAGG GGAAAGAGAA ATCACTTTGG GCTTTGTTGA TTTACTGCGT
LC503565_C   AACTTGAAGG GGAAAGAGAA ATCACTTTGG GCTTTGTTGA TTTACTGCGT
LC503573_C   AACTTGAAGG GGAAAGAGAA ATCACTTTGG GCTTTGTTGA TTTACTGCGT
LC503581_C   AACTTGAAGG GGAAAGAGAA ATCACTTTGG GCTTTGTTGA TTTACTGCGT
LC503589_C   AACTTGAAGG GGAAAGAGAA ATCACTTTGG GCTTTGTTGA TTTACTGCGT
LC503545_E   AACTTGAAGG GGAAAGAGAG ATCACTTTGG GCTTTGTTGA TTTACTGCGC
LC503553_E   AACTTGAAGG GGAAAGAGAG ATCACTTTGG GCTTTGTTGA TTTACTGCGC
LC503561_E   AACTTGAAGG GGAAAGAGAG ATCACTTTGG GCTTTGTTGA TTTACTGCGC
LC503569_E   AACTTGAAGG GGAAAGAGAG ATCACTTTGG GCTTTGTTGA TTTACTGCGC
LC503577_E   AACTTGAAGG GGAAAGAGAG ATCACTTTGG GCTTTGTTGA TTTACTGCGC
LC503585_E   AACTTGAAGG GGAAAGAGAG ATCACTTTGG GCTTTGTTGA TTTACTGCGC
LC503593_E   AACTTGAAGG GGAAAGAGAG ATCACTTTGG GCTTTGTTGA TTTACTGCGC

             ....|....| ....|....| ....|....| ....|....| ....|....|
                  1055       1065       1075       1085       1095
LC503541_C   GATGATTTTA TTGAAAAAGA TAGAAGTCGC GGTATTTTCT TCACCCAAGA
LC503549_C   GATGATTTTA TTGAAAAAGA TAGAAGTCGC GGTATTTTCT TCACCCAAGA
LC503557_C   GATGATTTTA TTGAAAAAGA TAGAAGTCGC GGTATTTTCT TCACCCAAGA
LC503565_C   GATGATTTTA TTGAAAAAGA TAGAAGTCGC GGTATTTTCT TCACCCAAGA
```

```
LC503573_C   GATGATTTTA TTGAAAAAGA TAGAAGTCGC GGTATTTTCT TCACCCAAGA
LC503581_C   GATGATTTTA TTGAAAAAGA TAGAAGTCGC GGTATTTTCT TCACCCAAGA
LC503589_C   GATGATTTTA TTGAAAAAGA TAGAAGTCGC GGTATTTTCT TCACCCAAGA
LC503545_E   GACGATTATA TTGAAAAAGA TCGAAGTCGC GGTATTTATT TCACCCAAGA
LC503553_E   GACGATTATA TTGAAAAAGA TCGAAGTCGC GGTATTTATT TCACCCAAGA
LC503561_E   GACGATTATA TTGAAAAAGA TCGAAGTCGC GGTATTTATT TCACCCAAGA
LC503569_E   GACGATTATA TTGAAAAAGA TCGAAGTCGC GGTATTTATT TCACCCAAGA
LC503577_E   GACGATTATA TTGAAAAAGA TCGAAGTCGC GGTATTTATT TCACCCAAGA
LC503585_E   GACGATTATA TTGAAAAAGA TCGAAGTCGC GGTATTTATT TCACCCAAGA
LC503593_E   GACGATTATA TTGAAAAAGA TCGAAGTCGC GGTATTTATT TCACCCAAGA

             ....|....| ....|....| ....|....| ....|....| ....|....|
                 1105       1115       1125       1135       1145
LC503541_C   TTGGGTCTCT CTACCAGGTG TTATACCTGT AGCTTCGGGC GGTATTCACG
LC503549_C   TTGGGTCTCT CTACCAGGTG TTATACCTGT AGCTTCGGGC GGTATTCACG
LC503557_C   TTGGGTCTCT CTACCAGGTG TTATACCTGT AGCTTCGGGC GGTATTCACG
LC503565_C   TTGGGTCTCT CTACCAGGTG TTATACCTGT AGCTTCGGGC GGTATTCACG
LC503573_C   TTGGGTCTCT CTACCAGGTG TTATACCTGT AGCTTCGGGC GGTATTCACG
LC503581_C   TTGGGTCTCT CTACCAGGTG TTATACCTGT AGCTTCGGGC GGTATTCACG
LC503589_C   TTGGGTCTCT CTACCAGGTG TTATACCTGT AGCTTCGGGC GGTATTCACG
LC503545_E   TTGGGTCTCT TTACCAGGTG TTCTGCCAGT AGCTTCGGGT GGTATTCACG
LC503553_E   TTGGGTCTCT TTACCAGGTG TTCTGCCAGT AGCTTCGGGT GGTATTCACG
LC503561_E   TTGGGTCTCT TTACCAGGTG TTCTGCCAGT AGCTTCGGGT GGTATTCACG
LC503569_E   TTGGGTCTCT TTACCAGGTG TTCTGCCAGT AGCTTCGGGT GGTATTCACG
LC503577_E   TTGGGTCTCT TTACCAGGTG TTCTGCCAGT AGCTTCGGGT GGTATTCACG
LC503585_E   TTGGGTCTCT TTACCAGGTG TTCTGCCAGT AGCTTCGGGT GGTATTCACG
LC503593_E   TTGGGTCTCT TTACCAGGTG TTCTGCCAGT AGCTTCGGGT GGTATTCACG

             ....|....| ....|....| ....|....| ....|....| ....|....|
                 1155       1165       1175       1185       1195
LC503541_C   TTTGGCATAT GCCTGCTCTG ACCGAGATCT TTGGGGATGA TTCCGTACTA
LC503549_C   TTTGGCATAT GCCTGCTCTG ACCGAGATCT TTGGGGATGA TTCCGTACTA
LC503557_C   TTTGGCATAT GCCTGCTCTG ACCGAGATCT TTGGGGATGA TTCCGTACTA
LC503565_C   TTTGGCATAT GCCTGCTCTG ACCGAGATCT TTGGGGATGA TTCCGTACTA
LC503573_C   TTTGGCATAT GCCTGCTCTG ACCGAGATCT TTGGGGATGA TTCCGTACTA
LC503581_C   TTTGGCATAT GCCTGCTCTG ACCGAGATCT TTGGGGATGA TTCCGTACTA
LC503589_C   TTTGGCATAT GCCTGCTCTG ACCGAGATCT TTGGGGATGA TTCCGTACTA
LC503545_E   TTTGGCATAT GCCTGCTCTG ACCGAGATCT TTGGGGATGA TTCCGTACTA
LC503553_E   TTTGGCATAT GCCTGCTCTG ACCGAGATCT TTGGGGATGA TTCCGTACTA
LC503561_E   TTTGGCATAT GCCTGCTCTG ACCGAGATCT TTGGGGATGA TTCCGTACTA
LC503569_E   TTTGGCATAT GCCTGCTCTG ACCGAGATCT TTGGGGATGA TTCCGTACTA
LC503577_E   TTTGGCATAT GCCTGCTCTG ACCGAGATCT TTGGGGATGA TTCCGTACTA
LC503585_E   TTTGGCATAT GCCTGCTCTG ACCGAGATCT TTGGGGATGA TTCCGTACTA
LC503593_E   TTTGGCATAT GCCTGCTCTG ACCGAGATCT TTGGGGATGA TTCCGTACTA

             ....|....| ....|....| ....|....| ....|....| ....|....|
                 1205       1215       1225       1235       1245
LC503541_C   CAGTTCGGTG GAGGAACTTT AGGGCACCCT TGGGGAAATG CACCCGGTGC
LC503549_C   CAGTTCGGTG GAGGAACTTT AGGGCACCCT TGGGGAAATG CACCCGGTGC
LC503557_C   CAGTTCGGTG GAGGAACTTT AGGGCACCCT TGGGGAAATG CACCCGGTGC
LC503565_C   CAGTTCGGTG GAGGAACTTT AGGGCACCCT TGGGGAAATG CACCCGGTGC
LC503573_C   CAGTTCGGTG GAGGAACTTT AGGGCACCCT TGGGGAAATG CACCCGGTGC
LC503581_C   CAGTTCGGTG GAGGAACTTT AGGGCACCCT TGGGGAAATG CACCCGGTGC
LC503589_C   CAGTTCGGTG GAGGAACTTT AGGGCACCCT TGGGGAAATG CACCCGGTGC
LC503545_E   CAGTTCGGTG GAGGAACTTT AGGGCACCCT TGGGGAAATG CACCCGGTGC
LC503553_E   CAGTTCGGTG GAGGAACTTT AGGGCACCCT TGGGGAAATG CACCCGGTGC
LC503561_E   CAGTTCGGTG GAGGAACTTT AGGGCACCCT TGGGGAAATG CACCCGGTGC
LC503569_E   CAGTTCGGTG GAGGAACTTT AGGGCACCCT TGGGGAAATG CACCCGGTGC
LC503577_E   CAGTTCGGTG GAGGAACTTT AGGGCACCCT TGGGGAAATG CACCCGGTGC
LC503585_E   CAGTTCGGTG GAGGAACTTT AGGGCACCCT TGGGGAAATG CACCCGGTGC
LC503593_E   CAGTTCGGTG GAGGAACTTT AGGGCACCCT TGGGGAAATG CACCCGGTGC
```

```
                ....|....| ....|....| ....|....| ....|....| ....|....|
                    1255       1265       1275       1285       1295
LC503541_C      CGTAGCTAAT CGAGTAGCTC TAGAAGCATG TGTACAAGCT CGTAATGAGG
LC503549_C      CGTAGCTAAT CGAGTAGCTC TAGAAGCATG TGTACAAGCT CGTAATGAGG
LC503557_C      CGTAGCTAAT CGAGTAGCTC TAGAAGCATG TGTACAAGCT CGTAATGAGG
LC503565_C      CGTAGCTAAT CGAGTAGCTC TAGAAGCATG TGTACAAGCT CGTAATGAGG
LC503573_C      CGTAGCTAAT CGAGTAGCTC TAGAAGCATG TGTACAAGCT CGTAATGAGG
LC503581_C      CGTAGCTAAT CGAGTAGCTC TAGAAGCATG TGTACAAGCT CGTAATGAGG
LC503589_C      CGTAGCTAAT CGAGTAGCTC TAGAAGCATG TGTACAAGCT CGTAATGAGG
LC503545_E      CGTAGCTAAT CGAGTAGCTC TAGAAGCATG TGTAAAAGCT CGTAATGAGG
LC503553_E      CGTAGCTAAT CGAGTAGCTC TAGAAGCATG TGTAAAAGCT CGTAATGAGG
LC503561_E      CGTAGCTAAT CGAGTAGCTC TAGAAGCATG TGTAAAAGCT CGTAATGAGG
LC503569_E      CGTAGCTAAT CGAGTAGCTC TAGAAGCATG TGTAAAAGCT CGTAATGAGG
LC503577_E      CGTAGCTAAT CGAGTAGCTC TAGAAGCATG TGTAAAAGCT CGTAATGAGG
LC503585_E      CGTAGCTAAT CGAGTAGCTC TAGAAGCATG TGTAAAAGCT CGTAATGAGG
LC503593_E      CGTAGCTAAT CGAGTAGCTC TAGAAGCATG TGTAAAAGCT CGTAATGAGG
```

```
                ....|....| ....|....| ....|....| ....|....| ....|....|
                    1305       1315       1325       1335       1345
LC503541_C      GACGCGATCT TGCTAGGGAG GGTAATGAAA TTATCCGTGA GGCTGCCAAA
LC503549_C      GACGCGATCT TGCTAGGGAG GGTAATGAAA TTATCCGTGA GGCTGCCAAA
LC503557_C      GACGCGATCT TGCTAGGGAG GGTAATGAAA TTATCCGTGA GGCTGCCAAA
LC503565_C      GACGCGATCT TGCTAGGGAG GGTAATGAAA TTATCCGTGA GGCTGCCAAA
LC503573_C      GACGCGATCT TGCTAGGGAG GGTAATGAAA TTATCCGTGA GGCTGCCAAA
LC503581_C      GACGCGATCT TGCTAGGGAG GGTAATGAAA TTATCCGTGA GGCTGCCAAA
LC503589_C      GACGCGATCT TGCTAGGGAG GGTAATGAAA TTATCCGTGA GGCTGCCAAA
LC503545_E      GACGCGACCT TGCTACTGAG GGTAATGAAA TTATCCGTGA GGCTGCTAAA
LC503553_E      GACGCGACCT TGCTACTGAG GGTAATGAAA TTATCCGTGA GGCTGCTAAA
LC503561_E      GACGCGACCT TGCTACTGAG GGTAATGAAA TTATCCGTGA GGCTGCTAAA
LC503569_E      GACGCGACCT TGCTACTGAG GGTAATGAAA TTATCCGTGA GGCTGCTAAA
LC503577_E      GACGCGACCT TGCTACTGAG GGTAATGAAA TTATCCGTGA GGCTGCTAAA
LC503585_E      GACGCGACCT TGCTACTGAG GGTAATGAAA TTATCCGTGA GGCTGCTAAA
LC503593_E      GACGCGACCT TGCTACTGAG GGTAATGAAA TTATCCGTGA GGCTGCTAAA
```

```
                ....|....| ....|....| ....|....| ....|....| ....|....|
                    1355       1365       1375       1385       1395
LC503541_C      TGGAGTCCTG AACTAGCTGC TGCTTGTGAA GTATGGAAGG CGATCAAATT
LC503549_C      TGGAGTCCTG AACTAGCTGC TGCTTGTGAA GTATGGAAGG CGATCAAATT
LC503557_C      TGGAGTCCTG AACTAGCTGC TGCTTGTGAA GTATGGAAGG CGATCAAATT
LC503565_C      TGGAGTCCTG AACTAGCTGC TGCTTGTGAA GTATGGAAGG CGATCAAATT
LC503573_C      TGGAGTCCTG AACTAGCTGC TGCTTGTGAA GTATGGAAGG CGATCAAATT
LC503581_C      TGGAGTCCTG AACTAGCTGC TGCTTGTGAA GTATGGAAGG CGATCAAATT
LC503589_C      TGGAGTCCTG AACTAGCTGC TGCTTGTGAA GTATGGAAGG CGATCAAATT
LC503545_E      TGGAGTCCTG AACTAGCTGC TGCTTGTGAA GTATGGAAGG AGATCAAATT
LC503553_E      TGGAGTCCTG AACTAGCTGC TGCTTGTGAA GTATGGAAGG AGATCAAATT
LC503561_E      TGGAGTCCTG AACTAGCTGC TGCTTGTGAA GTATGGAAGG AGATCAAATT
LC503569_E      TGGAGTCCTG AACTAGCTGC TGCTTGTGAA GTATGGAAGG AGATCAAATT
LC503577_E      TGGAGTCCTG AACTAGCTGC TGCTTGTGAA GTATGGAAGG AGATCAAATT
LC503585_E      TGGAGTCCTG AACTAGCTGC TGCTTGTGAA GTATGGAAGG AGATCAAATT
LC503593_E      TGGAGTCCTG AACTAGCTGC TGCTTGTGAA GTATGGAAGG AGATCAAATT
```

```
                ....|....| ....|....| ....|....| ....|....| ....|....|
                    1405       1415       1425       1435       1445
LC503541_C      TGAGTTTGAA CCAGTGGATA CTGTTGATGA ---------- ----ACCAGT
LC503549_C      TGAGTTTGAA CCAGTGGATA CTGTTGATGA ---------- ----ACCAGT
LC503557_C      TGAGTTTGAA CCAGTGGATA CTGTTGATGA ---------- ----ACCAGT
LC503565_C      TGAGTTTGAA CCAGTGGATA CTGTTGATGA ---------- ----ACCAGT
LC503573_C      TGAGTTTGAA CCAGTGGATA CTGTTGATGA ---------- ----ACCAGT
LC503581_C      TGAGTTTGAA CCAGTGGATA CTGTTGATGA ---------- ----ACCAGT
LC503589_C      TGAGTTTGAA CCAGTGGATA CTGTTGATGA ---------- ----ACCAGT
```

LC503545_E   TGAGTTTCAG GCAATGGATA CTTTGGATGA **CGATAAGGAT AAAG**ATAAG-
LC503553_E   TGAGTTTCAG GCAATGGATA CTTTGGATGA **CGATAAGGAT AAAG**ATAAG-
LC503561_E   TGAGTTTCAG GCAATGGATA CTTTGGATGA **CGATAAGGAT AAAG**ATAAG-
LC503569_E   TGAGTTTCAG GCAATGGATA CTTTGGATGA **CGATAAGGAT AAAG**ATAAG-
LC503577_E   TGAGTTTCAG GCAATGGATA CTTTGGATGA **CGATAAGGAT AAAG**ATAAG-
LC503585_E   TGAGTTTCAG GCAATGGATA CTTTGGATGA **CGATAAGGAT AAAG**ATAAG-
LC503593_E   TGAGTTTCAG GCAATGGATA CTTTGGATGA **CGATAAGGAT AAAG**ATAAG-

....|....|
1455
LC503541_C   CAAAAAATAA
LC503549_C   CAAAAAATAA
LC503557_C   CAAAAAATAA
LC503565_C   CAAAAAATAA
LC503573_C   CAAAAAATAA
LC503581_C   CAAAAAATAA
LC503589_C   CAAAAAATAA
LC503545_E   -AAGAGATAA
LC503553_E   -AAGAGATAA
LC503561_E   -AAGAGATAA
LC503569_E   -AAGAGATAA
LC503577_E   -AAGAGATAA
LC503585_E   -AAGAGATAA
LC503593_E   -AAGAGATAA

Supplementary Data S2. Alignment of *mat*K gene from *C. cinereum* and *E. sonchifolia*.

```
              ....|....| ....|....| ....|....| ....|....| ....|....|
                     5         15         25         35         45
LC503540_C    ATGGAGAAAT TCCAAAGTTA TTTAGGGCTA GATAGATCTC AACAACACTA
LC503548_C    ATGGAGAAAT TCCAAAGTTA TTTAGGGCTA GATAGATCTC AACAACACTA
LC503556_C    ATGGAGAAAT TCCAAAGTTA TTTAGGGCTA GATAGATCTC AACAACACTA
LC503564_C    ATGGAGAAAT TCCAAAGTTA TTTAGGGCTA GATAGATCTC AACAACACTA
LC503572_C    ATGGAGAAAT TCCAAAGTTA TTTAGGGCTA GATAGATCTC AACAACACTA
LC503580_C    ATGGAGAAAT TCCAAAGTTA TTTAGGGCTA GATAGATCTC AACAACACTA
LC503588_C    ATGGAGAAAT TCCAAAGTTA TTTAGGGCTA GATAGATCTC AACAACACTA
LC503544_E    ATGGATAAAT CCCAAATCTA TTTAGGGCTA GATAGATCTC AACAACACTA
LC503552_E    ATGGATAAAT CCCAAATCTA TTTAGGGCTA GATAGATCTC AACAACACTA
LC503560_E    ATGGATAAAT CCCAAATCTA TTTAGGGCTA GATAGATCTC AACAACACTA
LC503568_E    ATGGATAAAT CCCAAATCTA TTTAGGGCTA GATAGATCTC AACAACACTA
LC503576_E    ATGGATAAAT CCCAAATCTA TTTAGGGCTA GATAGATCTC AACAACACTA
LC503584_E    ATGGATAAAT CCCAAATCTA TTTAGGGCTA GATAGATCTC AACAACACTA
LC503592 E    ATGGATAAAT CCCAAATCTA TTTAGGGCTA GATAGATCTC AACAACACTA

              ....|....| ....|....| ....|....| ....|....| ....|....|
                    55         65         75         85         95
LC503540_C    CTTCTTATAT CCACTTATCT TTCAGGAGTA TATTTATGTA CTTGCTCATG
LC503548_C    CTTCTTATAT CCACTTATCT TTCAGGAGTA TATTTATGTA CTTGCTCATG
LC503556_C    CTTCTTATAT CCACTTATCT TTCAGGAGTA TATTTATGTA CTTGCTCATG
LC503564_C    CTTCTTATAT CCACTTATCT TTCAGGAGTA TATTTATGTA CTTGCTCATG
LC503572_C    CTTCTTATAT CCACTTATCT TTCAGGAGTA TATTTATGTA CTTGCTCATG
LC503580_C    CTTCTTATAT CCACTTATCT TTCAGGAGTA TATTTATGTA CTTGCTCATG
LC503588_C    CTTCTTATAT CCACTTATCT TTCAGGAGTA TATTTATGTA CTTGCTCATG
LC503544_E    CTTCTTATAT CCACTTATCT TTCAGGAGTA TATTTATGTA CTTGCTCATG
LC503552_E    CTTCTTATAT CCACTTATCT TTCAGGAGTA TATTTATGTA CTTGCTCATG
LC503560_E    CTTCTTATAT CCACTTATCT TTCAGGAGTA TATTTATGTA CTTGCTCATG
LC503568_E    CTTCTTATAT CCACTTATCT TTCAGGAGTA TATTTATGTA CTTGCTCATG
LC503576_E    CTTCTTATAT CCACTTATCT TTCAGGAGTA TATTTATGTA CTTGCTCATG
LC503584_E    CTTCTTATAT CCACTTATCT TTCAGGAGTA TATTTATGTA CTTGCTCATG
LC503592 E    CTTCTTATAT CCACTTATCT TTCAGGAGTA TATTTATGTA CTTGCTCATG

              ....|....| ....|....| ....|....| ....|....| ....|....|
                   105        115        125        135        145
LC503540_C    ATCATGGTTT AAATAGATCG ATTTTGTTGG AAAATACAGG TTATGACAAT
LC503548_C    ATCATGGTTT AAATAGATCG ATTTTGTTGG AAAATACAGG TTATGACAAT
LC503556_C    ATCATGGTTT AAATAGATCG ATTTTGTTGG AAAATACAGG TTATGACAAT
LC503564_C    ATCATGGTTT AAATAGATCG ATTTTGTTGG AAAATACAGG TTATGACAAT
LC503572_C    ATCATGGTTT AAATAGATCG ATTTTGTTGG AAAATACAGG TTATGACAAT
LC503580_C    ATCATGGTTT AAATAGATCG ATTTTGTTGG AAAATACAGG TTATGACAAT
LC503588_C    ATCATGGTTT AAATAGATCG ATTTTGTTGG AAAATACAGG TTATGACAAT
LC503544_E    ATCATGGTTT AAATAGATCT TTTTTGTTGG AAAATGCAGG TTATAACAAT
LC503552_E    ATCATGGTTT AAATAGATCT TTTTTGTTGG AAAATGCAGG TTATAACAAT
LC503560_E    ATCATGGTTT AAATAGATCT TTTTTGTTGG AAAATGCAGG TTATAACAAT
LC503568_E    ATCATGGTTT AAATAGATCT TTTTTGTTGG AAAATGCAGG TTATAACAAT
LC503576_E    ATCATGGTTT AAATAGATCT TTTTTGTTGG AAAATGCAGG TTATAACAAT
LC503584_E    ATCATGGTTT AAATAGATCT TTTTTGTTGG AAAATGCAGG TTATAACAAT
LC503592 E    ATCATGGTTT AAATAGATCT TTTTTGTTGG AAAATGCAGG TTATAACAAT

              ....|....| ....|....| ....|....| ....|....| ....|....|
                   155        165        175        185        195
LC503540_C    AAATCCAGCT TACTAATTGT GAAACGTTTA ATCATTCGAA TGTATCAACA
```

```
LC503548_C   AAATCCAGCT TACTAATTGT GAAACGTTTA ATCATTCGAA TGTATCAACA
LC503556_C   AAATCCAGCT TACTAATTGT GAAACGTTTA ATCATTCGAA TGTATCAACA
LC503564_C   AAATCCAGCT TACTAATTGT GAAACGTTTA ATCATTCGAA TGTATCAACA
LC503572_C   AAATCCAGCT TACTAATTGT GAAACGTTTA ATCATTCGAA TGTATCAACA
LC503580_C   AAATCCAGCT TACTAATTGT GAAACGTTTA ATCATTCGAA TGTATCAACA
LC503588_C   AAATCCAGCT TACTAATTGT GAAACGTTTA ATCATTCGAA TGTATCAACA
LC503544_E   AAGTGCAGCT TACTAATTGT GAAACGTTTA ATCATTCGAA TGTATCAACA
LC503552_E   AAGTGCAGCT TACTAATTGT GAAACGTTTA ATCATTCGAA TGTATCAACA
LC503560_E   AAGTGCAGCT TACTAATTGT GAAACGTTTA ATCATTCGAA TGTATCAACA
LC503568_E   AAGTGCAGCT TACTAATTGT GAAACGTTTA ATCATTCGAA TGTATCAACA
LC503576_E   AAGTGCAGCT TACTAATTGT GAAACGTTTA ATCATTCGAA TGTATCAACA
LC503584_E   AAGTGCAGCT TACTAATTGT GAAACGTTTA ATCATTCGAA TGTATCAACA
LC503592 E   AAGTGCAGCT TACTAATTGT GAAACGTTTA ATCATTCGAA TGTATCAACA

             ....|....| ....|....| ....|....| ....|....| ....|....|
                 205        215        225        235        245
LC503540_C   GAATCATTTG ATTCTTTCTG TTAATGATTC TAAACAGACT CCATTTTTGG
LC503548_C   GAATCATTTG ATTCTTTCTG TTAATGATTC TAAACAGACT CCATTTTTGG
LC503556_C   GAATCATTTG ATTCTTTCTG TTAATGATTC TAAACAGACT CCATTTTTGG
LC503564_C   GAATCATTTG ATTCTTTCTG TTAATGATTC TAAACAGACT CCATTTTTGG
LC503572_C   GAATCATTTG ATTCTTTCTG TTAATGATTC TAAACAGACT CCATTTTTGG
LC503580_C   GAATCATTTG ATTCTTTCTG TTAATGATTC TAAACAGACT CCATTTTTGG
LC503588_C   GAATCATTTG ATTCTTTCTG TTAATGATTC TAAACAGACT CCATTTTTGG
LC503544_E   GAA------- --CCTTTTTG TTAATGATTC TAAACAGACT CCATTTTTGG
LC503552_E   GAA------- --CCTTTTTG TTAATGATTC TAAACAGACT CCATTTTTGG
LC503560_E   GAA------- --CCTTTTTG TTAATGATTC TAAACAGACT CCATTTTTGG
LC503568_E   GAA------- --CCTTTTTG TTAATGATTC TAAACAGACT CCATTTTTGG
LC503576_E   GAA------- --CCTTTTTG TTAATGATTC TAAACAGACT CCATTTTTGG
LC503584_E   GAA------- --CCTTTTTG TTAATGATTC TAAACAGACT CCATTTTTGG
LC503592 E   GAA------- --CCTTTTTG TTAATGATTC TAAACAGACT CCATTTTTGG

             ....|....| ....|....| ....|....| ....|....| ....|....|
                 255        265        275        285        295
LC503540_C   GGCACAACAA TAATTTTTAT TCGCAAGTAA TCTCAGAGAT ATCTTCAATC
LC503548_C   GGCACAACAA TAATTTTTAT TCGCAAGTAA TCTCAGAGAT ATCTTCAATC
LC503556_C   GGCACAACAA TAATTTTTAT TCGCAAGTAA TCTCAGAGAT ATCTTCAATC
LC503564_C   GGCACAACAA TAATTTTTAT TCGCAAGTAA TCTCAGAGAT ATCTTCAATC
LC503572_C   GGCACAACAA TAATTTTTAT TCGCAAGTAA TCTCAGAGAT ATCTTCAATC
LC503580_C   GGCACAACAA TAATTTTTAT TCGCAAGTAA TCTCAGAGAT ATCTTCAATC
LC503588_C   GGCACAACAA TAATTTTTAT TCGCAAGTAA TCTCAGAGAT ATCTTCAATC
LC503544_E   GGCACAACAA TAATTTTTAT TCGCAAGTAA TGTCAGAGGT TTCTTCAATC
LC503552_E   GGCACAACAA TAATTTTTAT TCGCAAGTAA TGTCAGAGGT TTCTTCAATC
LC503560_E   GGCACAACAA TAATTTTTAT TCGCAAGTAA TGTCAGAGGT TTCTTCAATC
LC503568_E   GGCACAACAA TAATTTTTAT TCGCAAGTAA TGTCAGAGGT TTCTTCAATC
LC503576_E   GGCACAACAA TAATTTTTAT TCGCAAGTAA TGTCAGAGGT TTCTTCAATC
LC503584_E   GGCACAACAA TAATTTTTAT TCGCAAGTAA TGTCAGAGGT TTCTTCAATC
LC503592 E   GGCACAACAA TAATTTTTAT TCGCAAGTAA TGTCAGAGGT TTCTTCAATC

             ....|....| ....|....| ....|....| ....|....| ....|....|
                 305        315        325        335        345
LC503540_C   ATTATGGAAA TTCCATTGTC TCTGCGATTA ATATCTTCCC TAGAAAGGAA
LC503548_C   ATTATGGAAA TTCCATTGTC TCTGCGATTA ATATCTTCCC TAGAAAGGAA
LC503556_C   ATTATGGAAA TTCCATTGTC TCTGCGATTA ATATCTTCCC TAGAAAGGAA
LC503564_C   ATTATGGAAA TTCCATTGTC TCTGCGATTA ATATCTTCCC TAGAAAGGAA
LC503572_C   ATTATGGAAA TTCCATTGTC TCTGCGATTA ATATCTTCCC TAGAAAGGAA
LC503580_C   ATTATGGAAA TTCCATTGTC TCTGCGATTA ATATCTTCCC TAGAAAGGAA
LC503588_C   ATTATGGAAA TTCCATTGTC TCTGCGATTA ATATCTTCCC TAGAAAGGAA
```

```
LC503544_E   ATTATGGAAA TTCCATTGTC TTTGCGATTA ATATCTTCCC TAGAAAGGAA
LC503552_E   ATTATGGAAA TTCCATTGTC TTTGCGATTA ATATCTTCCC TAGAAAGGAA
LC503560_E   ATTATGGAAA TTCCATTGTC TTTGCGATTA ATATCTTCCC TAGAAAGGAA
LC503568_E   ATTATGGAAA TTCCATTGTC TTTGCGATTA ATATCTTCCC TAGAAAGGAA
LC503576_E   ATTATGGAAA TTCCATTGTC TTTGCGATTA ATATCTTCCC TAGAAAGGAA
LC503584_E   ATTATGGAAA TTCCATTGTC TTTGCGATTA ATATCTTCCC TAGAAAGGAA
LC503592 E   ATTATGGAAA TTCCATTGTC TTTGCGATTA ATATCTTCCC TAGAAAGGAA

             ....|....| ....|....| ....|....| ....|....| ....|....|
                  355        365        375        385        395
LC503540_C   AGGGGTAGTC AAATCCGATA ATTTACGATC AATTCATTCA ATATTTTCTT
LC503548_C   AGGGGTAGTC AAATCCGATA ATTTACGATC AATTCATTCA ATATTTTCTT
LC503556_C   AGGGGTAGTC AAATCCGATA ATTTACGATC AATTCATTCA ATATTTTCTT
LC503564_C   AGGGGTAGTC AAATCCGATA ATTTACGATC AATTCATTCA ATATTTTCTT
LC503572_C   AGGGGTAGTC AAATCCGATA ATTTACGATC AATTCATTCA ATATTTTCTT
LC503580_C   AGGGGTAGTC AAATCCGATA ATTTACGATC AATTCATTCA ATATTTTCTT
LC503588_C   AGGGGTAGTC AAATCCGATA ATTTACGATC AATTCATTCA ATATTTTCTT
LC503544_E   AGCTGTAGTT AAATCCAATA ATTTACGATC AATTCATTCA ATATTTTCTT
LC503552_E   AGCTGTAGTT AAATCCAATA ATTTACGATC AATTCATTCA ATATTTTCTT
LC503560_E   AGCTGTAGTT AAATCCAATA ATTTACGATC AATTCATTCA ATATTTTCTT
LC503568_E   AGCTGTAGTT AAATCCAATA ATTTACGATC AATTCATTCA ATATTTTCTT
LC503576_E   AGCTGTAGTT AAATCCAATA ATTTACGATC AATTCATTCA ATATTTTCTT
LC503584_E   AGCTGTAGTT AAATCCAATA ATTTACGATC AATTCATTCA ATATTTTCTT
LC503592 E   AGCTGTAGTT AAATCCAATA ATTTACGATC AATTCATTCA ATATTTTCTT

             ....|....| ....|....| ....|....| ....|....| ....|....|
                  405        415        425        435        445
LC503540_C   TTTTAGAGGA TAACTTTTCA CATTTAAATT ATGTATTAGA TATACTAATA
LC503548_C   TTTTAGAGGA TAACTTTTCA CATTTAAATT ATGTATTAGA TATACTAATA
LC503556_C   TTTTAGAGGA TAACTTTTCA CATTTAAATT ATGTATTAGA TATACTAATA
LC503564_C   TTTTAGAGGA TAACTTTTCA CATTTAAATT ATGTATTAGA TATACTAATA
LC503572_C   TTTTAGAGGA TAACTTTTCA CATTTAAATT ATGTATTAGA TATACTAATA
LC503580_C   TTTTAGAGGA TAACTTTTCA CATTTAAATT ATGTATTAGA TATACTAATA
LC503588_C   TTTTAGAGGA TAACTTTTCA CATTTAAATT ATGTATTAGA TATACTAATA
LC503544_E   TTTTAGAAGA CAACTTTTCA CATTTAAATT ATGTATTAGA TATACTAATA
LC503552_E   TTTTAGAAGA CAACTTTTCA CATTTAAATT ATGTATTAGA TATACTAATA
LC503560_E   TTTTAGAAGA CAACTTTTCA CATTTAAATT ATGTATTAGA TATACTAATA
LC503568_E   TTTTAGAAGA CAACTTTTCA CATTTAAATT ATGTATTAGA TATACTAATA
LC503576_E   TTTTAGAAGA CAACTTTTCA CATTTAAATT ATGTATTAGA TATACTAATA
LC503584_E   TTTTAGAAGA CAACTTTTCA CATTTAAATT ATGTATTAGA TATACTAATA
LC503592 E   TTTTAGAAGA CAACTTTTCA CATTTAAATT ATGTATTAGA TATACTAATA

             ....|....| ....|....| ....|....| ....|....| ....|....|
                  455        465        475        485        495
LC503540_C   CCTTACCCAG CCCATCTGGA AATCTTGGTT CAGGCTCTTC GCTATTGGAT
LC503548_C   CCTTACCCAG CCCATCTGGA AATCTTGGTT CAGGCTCTTC GCTATTGGAT
LC503556_C   CCTTACCCAG CCCATCTGGA AATCTTGGTT CAGGCTCTTC GCTATTGGAT
LC503564_C   CCTTACCCAG CCCATCTGGA AATCTTGGTT CAGGCTCTTC GCTATTGGAT
LC503572_C   CCTTACCCAG CCCATCTGGA AATCTTGGTT CAGGCTCTTC GCTATTGGAT
LC503580_C   CCTTACCCAG CCCATCTGGA AATCTTGGTT CAGGCTCTTC GCTATTGGAT
LC503588_C   CCTTACCCAG CCCATCTGGA AATCTTGGTT CAGGCTCTTC GCTATTGGAT
LC503544_E   CCTTACCCAG CCCATCTGGA AATATTGGTT CAGGCTCTTC GCTATTGGAT
LC503552_E   CCTTACCCAG CCCATCTGGA AATATTGGTT CAGGCTCTTC GCTATTGGAT
LC503560_E   CCTTACCCAG CCCATCTGGA AATATTGGTT CAGGCTCTTC GCTATTGGAT
LC503568_E   CCTTACCCAG CCCATCTGGA AATATTGGTT CAGGCTCTTC GCTATTGGAT
LC503576_E   CCTTACCCAG CCCATCTGGA AATATTGGTT CAGGCTCTTC GCTATTGGAT
LC503584_E   CCTTACCCAG CCCATCTGGA AATATTGGTT CAGGCTCTTC GCTATTGGAT
```

```
LC503592 E   CCTTACCCAG CCCATCTGGA AATATTGGTT CAGGCTCTTC GCTATTGGAT

             ....|....| ....|....| ....|....| ....|....| ....|....|
                505        515        525        535        545
LC503540_C   AAAAGATGCT TCCTCTTTGC ATTTATTAAG ATTCTTTCTC CATGAGTGTC
LC503548_C   AAAAGATGCT TCCTCTTTGC ATTTATTAAG ATTCTTTCTC CATGAGTGTC
LC503556_C   AAAAGATGCT TCCTCTTTGC ATTTATTAAG ATTCTTTCTC CATGAGTGTC
LC503564_C   AAAAGATGCT TCCTCTTTGC ATTTATTAAG ATTCTTTCTC CATGAGTGTC
LC503572_C   AAAAGATGCT TCCTCTTTGC ATTTATTAAG ATTCTTTCTC CATGAGTGTC
LC503580_C   AAAAGATGCT TCCTCTTTGC ATTTATTAAG ATTCTTTCTC CATGAGTGTC
LC503588_C   AAAAGATGCT TCCTCTTTGC ATTTATTAAG ATTCTTTCTC CATGAGTGTC
LC503544_E   AAAAGATGCT TCCTCTTTGC ATTTATTAAG ATTCTTTCTC CATGAGTGTC
LC503552_E   AAAAGATGCT TCCTCTTTGC ATTTATTAAG ATTCTTTCTC CATGAGTGTC
LC503560_E   AAAAGATGCT TCCTCTTTGC ATTTATTAAG ATTCTTTCTC CATGAGTGTC
LC503568_E   AAAAGATGCT TCCTCTTTGC ATTTATTAAG ATTCTTTCTC CATGAGTGTC
LC503576_E   AAAAGATGCT TCCTCTTTGC ATTTATTAAG ATTCTTTCTC CATGAGTGTC
LC503584_E   AAAAGATGCT TCCTCTTTGC ATTTATTAAG ATTCTTTCTC CATGAGTGTC
LC503592 E   AAAAGATGCT TCCTCTTTGC ATTTATTAAG ATTCTTTCTC CATGAGTGTC

             ....|....| ....|....| ....|....| ....|....| ....|....|
                555        565        575        585        595
LC503540_C   ATAATTGGGA TAGTCTTATT ACTTCAAATT CAAAGAAAGC CAGTTCTTCT
LC503548_C   ATAATTGGGA TAGTCTTATT ACTTCAAATT CAAAGAAAGC CAGTTCTTCT
LC503556_C   ATAATTGGGA TAGTCTTATT ACTTCAAATT CAAAGAAAGC CAGTTCTTCT
LC503564_C   ATAATTGGGA TAGTCTTATT ACTTCAAATT CAAAGAAAGC CAGTTCTTCT
LC503572_C   ATAATTGGGA TAGTCTTATT ACTTCAAATT CAAAGAAAGC CAGTTCTTCT
LC503580_C   ATAATTGGGA TAGTCTTATT ACTTCAAATT CAAAGAAAGC CAGTTCTTCT
LC503588_C   ATAATTGGGA TAGTCTTATT ACTTCAAATT CAAAGAAAGC CAGTTCTTCT
LC503544_E   ATAATTGGGA TAATCTTATT ACTTCAAATT CAAAGAAAGC CAGTTCTTCT
LC503552_E   ATAATTGGGA TAATCTTATT ACTTCAAATT CAAAGAAAGC CAGTTCTTCT
LC503560_E   ATAATTGGGA TAATCTTATT ACTTCAAATT CAAAGAAAGC CAGTTCTTCT
LC503568_E   ATAATTGGGA TAATCTTATT ACTTCAAATT CAAAGAAAGC CAGTTCTTCT
LC503576_E   ATAATTGGGA TAATCTTATT ACTTCAAATT CAAAGAAAGC CAGTTCTTCT
LC503584_E   ATAATTGGGA TAATCTTATT ACTTCAAATT CAAAGAAAGC CAGTTCTTCT
LC503592 E   ATAATTGGGA TAATCTTATT ACTTCAAATT CAAAGAAAGC CAGTTCTTCT

             ....|....| ....|....| ....|....| ....|....| ....|....|
                605        615        625        635        645
LC503540_C   TTTTCAAAAA GAAATCACAG ACTATTCTTC TTCCTATATA CTTCTCATGT
LC503548_C   TTTTCAAAAA GAAATCACAG ACTATTCTTC TTCCTATATA CTTCTCATGT
LC503556_C   TTTTCAAAAA GAAATCACAG ACTATTCTTC TTCCTATATA CTTCTCATGT
LC503564_C   TTTTCAAAAA GAAATCACAG ACTATTCTTC TTCCTATATA CTTCTCATGT
LC503572_C   TTTTCAAAAA GAAATCACAG ACTATTCTTC TTCCTATATA CTTCTCATGT
LC503580_C   TTTTCAAAAA GAAATCACAG ACTATTCTTC TTCCTATATA CTTCTCATGT
LC503588_C   TTTTCAAAAA GAAATCACAG ACTATTCTTC TTCCTATATA CTTCTCATGT
LC503544_E   TTTTCAAAAA TAAATAAAAG ACTATTCTTC TTCCTATATA CTTATCATGT
LC503552_E   TTTTCAAAAA TAAATAAAAG ACTATTCTTC TTCCTATATA CTTATCATGT
LC503560_E   TTTTCAAAAA TAAATAAAAG ACTATTCTTC TTCCTATATA CTTATCATGT
LC503568_E   TTTTCAAAAA TAAATAAAAG ACTATTCTTC TTCCTATATA CTTATCATGT
LC503576_E   TTTTCAAAAA TAAATAAAAG ACTATTCTTC TTCCTATATA CTTATCATGT
LC503584_E   TTTTCAAAAA TAAATAAAAG ACTATTCTTC TTCCTATATA CTTATCATGT
LC503592 E   TTTTCAAAAA TAAATAAAAG ACTATTCTTC TTCCTATATA CTTATCATGT

             ....|....| ....|....| ....|....| ....|....| ....|....|
                655        665        675        685        695
LC503540_C   ATGTGAATAT GAATCTGTCT TCCTCTTTCT CCGTAACCAA TCTTCTCACT
LC503548_C   ATGTGAATAT GAATCTGTCT TCCTCTTTCT CCGTAACCAA TCTTCTCACT
```

```
LC503556_C   ATGTGAATAT GAATCTGTCT TCCTCTTTCT CCGTAACCAA TCTTCTCACT
LC503564_C   ATGTGAATAT GAATCTGTCT TCCTCTTTCT CCGTAACCAA TCTTCTCACT
LC503572_C   ATGTGAATAT GAATCTGTCT TCCTCTTTCT CCGTAACCAA TCTTCTCACT
LC503580_C   ATGTGAATAT GAATCTGTCT TCCTCTTTCT CCGTAACCAA TCTTCTCACT
LC503588_C   ATGTGAATAT GAATCTGTCT TCCTCTTTCT CCGTAACCAA TCTTCTCACT
LC503544_E   ATGTGAATAT GAATCCGGCT TCTTATTTCT CCGTAACCAA TCTTCTCACT
LC503552_E   ATGTGAATAT GAATCCGGCT TCTTATTTCT CCGTAACCAA TCTTCTCACT
LC503560_E   ATGTGAATAT GAATCCGGCT TCTTATTTCT CCGTAACCAA TCTTCTCACT
LC503568_E   ATGTGAATAT GAATCCGGCT TCTTATTTCT CCGTAACCAA TCTTCTCACT
LC503576_E   ATGTGAATAT GAATCCGGCT TCTTATTTCT CCGTAACCAA TCTTCTCACT
LC503584_E   ATGTGAATAT GAATCCGGCT TCTTATTTCT CCGTAACCAA TCTTCTCACT
LC503592 E   ATGTGAATAT GAATCCGGCT TCTTATTTCT CCGTAACCAA TCTTCTCACT

             ....|....| ....|....| ....|....| ....|....| ....|....|
                  705        715        725        735        745
LC503540_C   TCCGATCAAC ATCTTCTGGA ACCCTTATTG AACGAATATA TTTCTATGGA
LC503548_C   TCCGATCAAC ATCTTCTGGA ACCCTTATTG AACGAATATA TTTCTATGGA
LC503556_C   TCCGATCAAC ATCTTCTGGA ACCCTTATTG AACGAATATA TTTCTATGGA
LC503564_C   TCCGATCAAC ATCTTCTGGA ACCCTTATTG AACGAATATA TTTCTATGGA
LC503572_C   TCCGATCAAC ATCTTCTGGA ACCCTTATTG AACGAATATA TTTCTATGGA
LC503580_C   TCCGATCAAC ATCTTCTGGA ACCCTTATTG AACGAATATA TTTCTATGGA
LC503588_C   TCCGATCAAC ATCTTCTGGA ACCCTTATTG AACGAATATA TTTCTATGGA
LC503544_E   TACGATCAAC ATCTTCTGGA GTCCTTATTG AACGAATATA TTTCTATGGA
LC503552_E   TACGATCAAC ATCTTCTGGA GTCCTTATTG AACGAATATA TTTCTATGGA
LC503560_E   TACGATCAAC ATCTTCTGGA GTCCTTATTG AACGAATATA TTTCTATGGA
LC503568_E   TACGATCAAC ATCTTCTGGA GTCCTTATTG AACGAATATA TTTCTATGGA
LC503576_E   TACGATCAAC ATCTTCTGGA GTCCTTATTG AACGAATATA TTTCTATGGA
LC503584_E   TACGATCAAC ATCTTCTGGA GTCCTTATTG AACGAATATA TTTCTATGGA
LC503592 E   TACGATCAAC ATCTTCTGGA GTCCTTATTG AACGAATATA TTTCTATGGA

             ....|....| ....|....| ....|....| ....|....| ....|....|
                  755        765        775        785        795
LC503540_C   AAAATAGAGC ATCTTGCAGA AGTCTTTGCC AGGGCTTTTC AAGCCAATTT
LC503548_C   AAAATAGAGC ATCTTGCAGA AGTCTTTGCC AGGGCTTTTC AAGCCAATTT
LC503556_C   AAAATAGAGC ATCTTGCAGA AGTCTTTGCC AGGGCTTTTC AAGCCAATTT
LC503564_C   AAAATAGAGC ATCTTGCAGA AGTCTTTGCC AGGGCTTTTC AAGCCAATTT
LC503572_C   AAAATAGAGC ATCTTGCAGA AGTCTTTGCC AGGGCTTTTC AAGCCAATTT
LC503580_C   AAAATAGAGC ATCTTGCAGA AGTCTTTGCC AGGGCTTTTC AAGCCAATTT
LC503588_C   AAAATAGAGC ATCTTGCAGA AGTCTTTGCC AGGGCTTTTC AAGCCAATTT
LC503544_E   AAAATAGAGC ATCTTGCAGA AATCTTTGCC AGGGCTTTTC AAGCGAATTT
LC503552_E   AAAATAGAGC ATCTTGCAGA AATCTTTGCC AGGGCTTTTC AAGCGAATTT
LC503560_E   AAAATAGAGC ATCTTGCAGA AATCTTTGCC AGGGCTTTTC AAGCGAATTT
LC503568_E   AAAATAGAGC ATCTTGCAGA AATCTTTGCC AGGGCTTTTC AAGCGAATTT
LC503576_E   AAAATAGAGC ATCTTGCAGA AATCTTTGCC AGGGCTTTTC AAGCGAATTT
LC503584_E   AAAATAGAGC ATCTTGCAGA AATCTTTGCC AGGGCTTTTC AAGCGAATTT
LC503592 E   AAAATAGAGC ATCTTGCAGA AATCTTTGCC AGGGCTTTTC AAGCGAATTT

             ....|....| ....|....| ....|....| ....|....| ....|....|
                  805        815        825        835        845
LC503540_C   ATGGTTGTTC AAAGATCCTT TCATGCATTA TGTTAGGTAT CAAGGAAAAT
LC503548_C   ATGGTTGTTC AAAGATCCTT TCATGCATTA TGTTAGGTAT CAAGGAAAAT
LC503556_C   ATGGTTGTTC AAAGATCCTT TCATGCATTA TGTTAGGTAT CAAGGAAAAT
LC503564_C   ATGGTTGTTC AAAGATCCTT TCATGCATTA TGTTAGGTAT CAAGGAAAAT
LC503572_C   ATGGTTGTTC AAAGATCCTT TCATGCATTA TGTTAGGTAT CAAGGAAAAT
LC503580_C   ATGGTTGTTC AAAGATCCTT TCATGCATTA TGTTAGGTAT CAAGGAAAAT
LC503588_C   ATGGTTGTTC AAAGATCCTT TCATGCATTA TGTTAGGTAT CAAGGAAAAT
LC503544_E   ATGGTTGTTC AAAGATCCTT TCATGCATTG TGTTAGGTAT CAAGGAAAAT
```

```
LC503552_E   ATGGTTGTTC AAAGATCCTT TCATGCATTG TGTTAGGTAT CAAGGAAAAT
LC503560_E   ATGGTTGTTC AAAGATCCTT TCATGCATTG TGTTAGGTAT CAAGGAAAAT
LC503568_E   ATGGTTGTTC AAAGATCCTT TCATGCATTG TGTTAGGTAT CAAGGAAAAT
LC503576_E   ATGGTTGTTC AAAGATCCTT TCATGCATTG TGTTAGGTAT CAAGGAAAAT
LC503584_E   ATGGTTGTTC AAAGATCCTT TCATGCATTG TGTTAGGTAT CAAGGAAAAT
LC503592_E   ATGGTTGTTC AAAGATCCTT TCATGCATTG TGTTAGGTAT CAAGGAAAAT

             ....|....| ....|....| ....|....| ....|....| ....|....|
                  855        865        875        885        895
LC503540_C   CAATTCTTGC TTCAAAAGGG ACGTTTCTTT TAATGAATAA ATGGAAATAT
LC503548_C   CAATTCTTGC TTCAAAAGGG ACGTTTCTTT TAATGAATAA ATGGAAATAT
LC503556_C   CAATTCTTGC TTCAAAAGGG ACGTTTCTTT TAATGAATAA ATGGAAATAT
LC503564_C   CAATTCTTGC TTCAAAAGGG ACGTTTCTTT TAATGAATAA ATGGAAATAT
LC503572_C   CAATTCTTGC TTCAAAAGGG ACGTTTCTTT TAATGAATAA ATGGAAATAT
LC503580_C   CAATTCTTGC TTCAAAAGGG ACGTTTCTTT TAATGAATAA ATGGAAATAT
LC503588_C   CAATTCTTGC TTCAAAAGGG ACGTTTCTTT TAATGAATAA ATGGAAATAT
LC503544_E   CAATTATTGC TTCAAAAGGG ACGTTTCTTT TGATGAACAA ATGGAAATAT
LC503552_E   CAATTATTGC TTCAAAAGGG ACGTTTCTTT TGATGAACAA ATGGAAATAT
LC503560_E   CAATTATTGC TTCAAAAGGG ACGTTTCTTT TGATGAACAA ATGGAAATAT
LC503568_E   CAATTATTGC TTCAAAAGGG ACGTTTCTTT TGATGAACAA ATGGAAATAT
LC503576_E   CAATTATTGC TTCAAAAGGG ACGTTTCTTT TGATGAACAA ATGGAAATAT
LC503584_E   CAATTATTGC TTCAAAAGGG ACGTTTCTTT TGATGAACAA ATGGAAATAT
LC503592_E   CAATTATTGC TTCAAAAGGG ACGTTTCTTT TGATGAACAA ATGGAAATAT

             ....|....| ....|....| ....|....| ....|....| ....|....|
                  905        915        925        935        945
LC503540_C   TACTTTGTAA ATTTCTGGAA ATCCTATTTT TACCTGTGGT CTCAACCAGG
LC503548_C   TACTTTGTAA ATTTCTGGAA ATCCTATTTT TACCTGTGGT CTCAACCAGG
LC503556_C   TACTTTGTAA ATTTCTGGAA ATCCTATTTT TACCTGTGGT CTCAACCAGG
LC503564_C   TACTTTGTAA ATTTCTGGAA ATCCTATTTT TACCTGTGGT CTCAACCAGG
LC503572_C   TACTTTGTAA ATTTCTGGAA ATCCTATTTT TACCTGTGGT CTCAACCAGG
LC503580_C   TACTTTGTAA ATTTCTGGAA ATCCTATTTT TACCTGTGGT CTCAACCAGG
LC503588_C   TACTTTGTAA ATTTCTGGAA ATCCTATTTT TACCTGTGGT CTCAACCAGG
LC503544_E   TACTTTGTCA ATTTCTCGAA ATCCCATTTT TATCTCTGGT CTCAACCAGG
LC503552_E   TACTTTGTCA ATTTCTCGAA ATCCCATTTT TATCTCTGGT CTCAACCAGG
LC503560_E   TACTTTGTCA ATTTCTCGAA ATCCCATTTT TATCTCTGGT CTCAACCAGG
LC503568_E   TACTTTGTCA ATTTCTCGAA ATCCCATTTT TATCTCTGGT CTCAACCAGG
LC503576_E   TACTTTGTCA ATTTCTCGAA ATCCCATTTT TATCTCTGGT CTCAACCAGG
LC503584_E   TACTTTGTCA ATTTCTCGAA ATCCCATTTT TATCTCTGGT CTCAACCAGG
LC503592_E   TACTTTGTCA ATTTCTCGAA ATCCCATTTT TATCTCTGGT CTCAACCAGG

             ....|....| ....|....| ....|....| ....|....| ....|....|
                  955        965        975        985        995
LC503540_C   AAGGATTTAT ATAAACCAAT TATCCAATCA TTCCCTTTAC TTTCTCGGTT
LC503548_C   AAGGATTTAT ATAAACCAAT TATCCAATCA TTCCCTTTAC TTTCTCGGTT
LC503556_C   AAGGATTTAT ATAAACCAAT TATCCAATCA TTCCCTTTAC TTTCTCGGTT
LC503564_C   AAGGATTTAT ATAAACCAAT TATCCAATCA TTCCCTTTAC TTTCTCGGTT
LC503572_C   AAGGATTTAT ATAAACCAAT TATCCAATCA TTCCCTTTAC TTTCTCGGTT
LC503580_C   AAGGATTTAT ATAAACCAAT TATCCAATCA TTCCCTTTAC TTTCTCGGTT
LC503588_C   AAGGATTTAT ATAAACCAAT TATCCAATCA TTCCCTTTAC TTTCTCGGTT
LC503544_E   AAGAATTTAT ATAAACAAAT TATCCAATCA TTCCCTTGAC TTTCTGGGTT
LC503552_E   AAGAATTTAT ATAAACAAAT TATCCAATCA TTCCCTTGAC TTTCTGGGTT
LC503560_E   AAGAATTTAT ATAAACAAAT TATCCAATCA TTCCCTTGAC TTTCTGGGTT
LC503568_E   AAGAATTTAT ATAAACAAAT TATCCAATCA TTCCCTTGAC TTTCTGGGTT
LC503576_E   AAGAATTTAT ATAAACAAAT TATCCAATCA TTCCCTTGAC TTTCTGGGTT
LC503584_E   AAGAATTTAT ATAAACAAAT TATCCAATCA TTCCCTTGAC TTTCTGGGTT
LC503592_E   AAGAATTTAT ATAAACAAAT TATCCAATCA TTCCCTTGAC TTTCTGGGTT
```

```
                ....|....| ....|....| ....|....| ....|....| ....|....|
                     1005       1015       1025       1035       1045
LC503540_C      ATCGTTCAAG TGTCCGGCTA AAGCCTTCAA TGGTACGCAG TCAAATGCTA
LC503548_C      ATCGTTCAAG TGTCCGGCTA AAGCCTTCAA TGGTACGCAG TCAAATGCTA
LC503556_C      ATCGTTCAAG TGTCCGGCTA AAGCCTTCAA TGGTACGCAG TCAAATGCTA
LC503564_C      ATCGTTCAAG TGTCCGGCTA AAGCCTTCAA TGGTACGCAG TCAAATGCTA
LC503572_C      ATCGTTCAAG TGTCCGGCTA AAGCCTTCAA TGGTACGCAG TCAAATGCTA
LC503580_C      ATCGTTCAAG TGTCCGGCTA AAGCCTTCAA TGGTACGCAG TCAAATGCTA
LC503588_C      ATCGTTCAAG TGTCCGGCTA AAGCCTTCAA TGGTACGCAG TCAAATGCTA
LC503544_E      ATCGTTCAAG TGTGCGGCTA AAGTCTTCAA TGGTACGCAG TCAAATGCTA
LC503552_E      ATCGTTCAAG TGTGCGGCTA AAGTCTTCAA TGGTACGCAG TCAAATGCTA
LC503560_E      ATCGTTCAAG TGTGCGGCTA AAGTCTTCAA TGGTACGCAG TCAAATGCTA
LC503568_E      ATCGTTCAAG TGTGCGGCTA AAGTCTTCAA TGGTACGCAG TCAAATGCTA
LC503576_E      ATCGTTCAAG TGTGCGGCTA AAGTCTTCAA TGGTACGCAG TCAAATGCTA
LC503584_E      ATCGTTCAAG TGTGCGGCTA AAGTCTTCAA TGGTACGCAG TCAAATGCTA
LC503592 E      ATCGTTCAAG TGTGCGGCTA AAGTCTTCAA TGGTACGCAG TCAAATGCTA

                ....|....| ....|....| ....|....| ....|....| ....|....|
                     1055       1065       1075       1085       1095
LC503540_C      GAAAATGCAT TTCTAATCGA TAATGCTATT AAGAAGTTTG ATACTATTGT
LC503548_C      GAAAATGCAT TTCTAATCGA TAATGCTATT AAGAAGTTTG ATACTATTGT
LC503556_C      GAAAATGCAT TTCTAATCGA TAATGCTATT AAGAAGTTTG ATACTATTGT
LC503564_C      GAAAATGCAT TTCTAATCGA TAATGCTATT AAGAAGTTTG ATACTATTGT
LC503572_C      GAAAATGCAT TTCTAATCGA TAATGCTATT AAGAAGTTTG ATACTATTGT
LC503580_C      GAAAATGCAT TTCTAATCGA TAATGCTATT AAGAAGTTTG ATACTATTGT
LC503588_C      GAAAATGCAT TTCTAATCGA TAATGCTATT AAGAAGTTTG ATACTATTGT
LC503544_E      GAAAATGCAT TTCTAATTGA TAATGCTATT AAGAAGCTTG ATACCCTTGT
LC503552_E      GAAAATGCAT TTCTAATTGA TAATGCTATT AAGAAGCTTG ATACCCTTGT
LC503560_E      GAAAATGCAT TTCTAATTGA TAATGCTATT AAGAAGCTTG ATACCCTTGT
LC503568_E      GAAAATGCAT TTCTAATTGA TAATGCTATT AAGAAGCTTG ATACCCTTGT
LC503576_E      GAAAATGCAT TTCTAATTGA TAATGCTATT AAGAAGCTTG ATACCCTTGT
LC503584_E      GAAAATGCAT TTCTAATTGA TAATGCTATT AAGAAGCTTG ATACCCTTGT
LC503592 E      GAAAATGCAT TTCTAATTGA TAATGCTATT AAGAAGCTTG ATACCCTTGT

                ....|....| ....|....| ....|....| ....|....| ....|....|
                     1105       1115       1125       1135       1145
LC503540_C      TCCAATTATG CCTTTGATTG GATTATTGGC TAAATCGAAA TTTTGTAACG
LC503548_C      TCCAATTATG CCTTTGATTG GATTATTGGC TAAATCGAAA TTTTGTAACG
LC503556_C      TCCAATTATG CCTTTGATTG GATTATTGGC TAAATCGAAA TTTTGTAACG
LC503564_C      TCCAATTATG CCTTTGATTG GATTATTGGC TAAATCGAAA TTTTGTAACG
LC503572_C      TCCAATTATG CCTTTGATTG GATTATTGGC TAAATCGAAA TTTTGTAACG
LC503580_C      TCCAATTATG CCTTTGATTG GATTATTGGC TAAATCGAAA TTTTGTAACG
LC503588_C      TCCAATTATG CCTTTGATTG GATTATTGGC TAAATCGAAA TTTTGTAACG
LC503544_E      TCCAATTATG CCTCCGATTG TATCATTGGC TAAATCGAAA TTTTGTAACG
LC503552_E      TCCAATTATG CCTCCGATTG TATCATTGGC TAAATCGAAA TTTTGTAACG
LC503560_E      TCCAATTATG CCTCCGATTG TATCATTGGC TAAATCGAAA TTTTGTAACG
LC503568_E      TCCAATTATG CCTCCGATTG TATCATTGGC TAAATCGAAA TTTTGTAACG
LC503576_E      TCCAATTATG CCTCCGATTG TATCATTGGC TAAATCGAAA TTTTGTAACG
LC503584_E      TCCAATTATG CCTCCGATTG TATCATTGGC TAAATCGAAA TTTTGTAACG
LC503592 E      TCCAATTATG CCTCCGATTG TATCATTGGC TAAATCGAAA TTTTGTAACG

                ....|....| ....|....| ....|....| ....|....| ....|....|
                     1155       1165       1175       1185       1195
LC503540_C      CATTGGGGCA TCCTATTGGT AAGGCGATTT GGACCGATTT ATCGGATTCT
LC503548_C      CATTGGGGCA TCCTATTGGT AAGGCGATTT GGACCGATTT ATCGGATTCT
LC503556_C      CATTGGGGCA TCCTATTGGT AAGGCGATTT GGACCGATTT ATCGGATTCT
```

```
LC503564_C   CATTGGGGCA TCCTATTGGT AAGGCGATTT GGACCGATTT ATCGGATTCT
LC503572_C   CATTGGGGCA TCCTATTGGT AAGGCGATTT GGACCGATTT ATCGGATTCT
LC503580_C   CATTGGGGCA TCCTATTGGT AAGGCGATTT GGACCGATTT ATCGGATTCT
LC503588_C   CATTGGGGCA TCCTATTGGT AAGGCGATTT GGACCGATTT ATCGGATTCT
LC503544_E   CAGTGGGGCG CCCTATTGGT AAGGCCATTT GGACAGATTT ATCAGATTCT
LC503552_E   CAGTGGGGCG CCCTATTGGT AAGGCCATTT GGACAGATTT ATCAGATTCT
LC503560_E   CAGTGGGGCG CCCTATTGGT AAGGCCATTT GGACAGATTT ATCAGATTCT
LC503568_E   CAGTGGGGCG CCCTATTGGT AAGGCCATTT GGACAGATTT ATCAGATTCT
LC503576_E   CAGTGGGGCG CCCTATTGGT AAGGCCATTT GGACAGATTT ATCAGATTCT
LC503584_E   CAGTGGGGCG CCCTATTGGT AAGGCCATTT GGACAGATTT ATCAGATTCT
LC503592 E   CAGTGGGGCG CCCTATTGGT AAGGCCATTT GGACAGATTT ATCAGATTCT

             ....|....| ....|....| ....|....| ....|....| ....|....|
                 1205       1215       1225       1235       1245
LC503540_C   GATATTATTG ACCGCTTTGG GCGTATATAC AGAAAGATTT CTCATTATCA
LC503548_C   GATATTATTG ACCGCTTTGG GCGTATATAC AGAAAGATTT CTCATTATCA
LC503556_C   GATATTATTG ACCGCTTTGG GCGTATATAC AGAAAGATTT CTCATTATCA
LC503564_C   GATATTATTG ACCGCTTTGG GCGTATATAC AGAAAGATTT CTCATTATCA
LC503572_C   GATATTATTG ACCGCTTTGG GCGTATATAC AGAAAGATTT CTCATTATCA
LC503580_C   GATATTATTG ACCGCTTTGG GCGTATATAC AGAAAGATTT CTCATTATCA
LC503588_C   GATATTATTG ACCGCTTTGG GCGTATATAC AGAAAGATTT CTCATTATCA
LC503544_E   GATATTATTG AGCGCTGTGG GCGTATATAC AGAAATCTTT CTCATTATCA
LC503552_E   GATATTATTG AGCGCTGTGG GCGTATATAC AGAAATCTTT CTCATTATCA
LC503560_E   GATATTATTG AGCGCTGTGG GCGTATATAC AGAAATCTTT CTCATTATCA
LC503568_E   GATATTATTG AGCGCTGTGG GCGTATATAC AGAAATCTTT CTCATTATCA
LC503576_E   GATATTATTG AGCGCTGTGG GCGTATATAC AGAAATCTTT CTCATTATCA
LC503584_E   GATATTATTG AGCGCTGTGG GCGTATATAC AGAAATCTTT CTCATTATCA
LC503592 E   GATATTATTG AGCGCTGTGG GCGTATATAC AGAAATCTTT CTCATTATCA

             ....|....| ....|....| ....|....| ....|....| ....|....|
                 1255       1265       1275       1285       1295
LC503540_C   TAGTGGATCT TCAAAAAAAA AGAGTTTGTA TCGAGTAAAG TATATACTTC
LC503548_C   TAGTGGATCT TCAAAAAAAA AGAGTTTGTA TCGAGTAAAG TATATACTTC
LC503556_C   TAGTGGATCT TCAAAAAAAA AGAGTTTGTA TCGAGTAAAG TATATACTTC
LC503564_C   TAGTGGATCT TCAAAAAAAA AGAGTTTGTA TCGAGTAAAG TATATACTTC
LC503572_C   TAGTGGATCT TCAAAAAAAA AGAGTTTGTA TCGAGTAAAG TATATACTTC
LC503580_C   TAGTGGATCT TCAAAAAAAA AGAGTTTGTA TCGAGTAAAG TATATACTTC
LC503588_C   TAGTGGATCT TCAAAAAAAA AGAGTTTGTA TCGAGTAAAG TATATACTTC
LC503544_E   TAGTGGATCT TCAAAAAAAA AGAGTTTGTA TCGCGTAAAG TATATACTTC
LC503552_E   TAGTGGATCT TCAAAAAAAA AGAGTTTGTA TCGCGTAAAG TATATACTTC
LC503560_E   TAGTGGATCT TCAAAAAAAA AGAGTTTGTA TCGCGTAAAG TATATACTTC
LC503568_E   TAGTGGATCT TCAAAAAAAA AGAGTTTGTA TCGCGTAAAG TATATACTTC
LC503576_E   TAGTGGATCT TCAAAAAAAA AGAGTTTGTA TCGCGTAAAG TATATACTTC
LC503584_E   TAGTGGATCT TCAAAAAAAA AGAGTTTGTA TCGCGTAAAG TATATACTTC
LC503592 E   TAGTGGATCT TCAAAAAAAA AGAGTTTGTA TCGCGTAAAG TATATACTTC

             ....|....| ....|....| ....|....| ....|....| ....|....|
                 1305       1315       1325       1335       1345
LC503540_C   GACTTTCTTG TGCTAGAACT TTAGCTCGTA AGCATAAAAG TACTGTACGT
LC503548_C   GACTTTCTTG TGCTAGAACT TTAGCTCGTA AGCATAAAAG TACTGTACGT
LC503556_C   GACTTTCTTG TGCTAGAACT TTAGCTCGTA AGCATAAAAG TACTGTACGT
LC503564_C   GACTTTCTTG TGCTAGAACT TTAGCTCGTA AGCATAAAAG TACTGTACGT
LC503572_C   GACTTTCTTG TGCTAGAACT TTAGCTCGTA AGCATAAAAG TACTGTACGT
LC503580_C   GACTTTCTTG TGCTAGAACT TTAGCTCGTA AGCATAAAAG TACTGTACGT
LC503588_C   GACTTTCTTG TGCTAGAACT TTAGCTCGTA AGCATAAAAG TACTGTACGT
LC503544_E   GACTTTCTTG TGCTAGAACT TTAGCTCGTA AGCATAAAAG TACTGTACGT
LC503552_E   GACTTTCTTG TGCTAGAACT TTAGCTCGTA AGCATAAAAG TACTGTACGT
```

```
LC503560_E   GACTTTCTTG TGCTAGAACT TTAGCTCGTA AGCATAAAAG TACTGTACGT
LC503568_E   GACTTTCTTG TGCTAGAACT TTAGCTCGTA AGCATAAAAG TACTGTACGT
LC503576_E   GACTTTCTTG TGCTAGAACT TTAGCTCGTA AGCATAAAAG TACTGTACGT
LC503584_E   GACTTTCTTG TGCTAGAACT TTAGCTCGTA AGCATAAAAG TACTGTACGT
LC503592_E   GACTTTCTTG TGCTAGAACT TTAGCTCGTA AGCATAAAAG TACTGTACGT

             ....|....| ....|....| ....|....| ....|....| ....|....|
                 1355       1365       1375       1385       1395
LC503540_C   GCCTTTTTGA AAAGATTTGG ATCGGAATTA TTGGAAGAAT TCTTTACGGA
LC503548_C   GCCTTTTTGA AAAGATTTGG ATCGGAATTA TTGGAAGAAT TCTTTACGGA
LC503556_C   GCCTTTTTGA AAAGATTTGG ATCGGAATTA TTGGAAGAAT TCTTTACGGA
LC503564_C   GCCTTTTTGA AAAGATTTGG ATCGGAATTA TTGGAAGAAT TCTTTACGGA
LC503572_C   GCCTTTTTGA AAAGATTTGG ATCGGAATTA TTGGAAGAAT TCTTTACGGA
LC503580_C   GCCTTTTTGA AAAGATTTGG ATCGGAATTA TTGGAAGAAT TCTTTACGGA
LC503588_C   GCCTTTTTGA AAAGATTTGG ATCGGAATTA TTGGAAGAAT TCTTTACGGA
LC503544_E   GCTTTTTTGA AAAGATTAGG TTCGGAATTA TTGGAAGAAT TCTTTACGGA
LC503552_E   GCTTTTTTGA AAAGATTAGG TTCGGAATTA TTGGAAGAAT TCTTTACGGA
LC503560_E   GCTTTTTTGA AAAGATTAGG TTCGGAATTA TTGGAAGAAT TCTTTACGGA
LC503568_E   GCTTTTTTGA AAAGATTAGG TTCGGAATTA TTGGAAGAAT TCTTTACGGA
LC503576_E   GCTTTTTTGA AAAGATTAGG TTCGGAATTA TTGGAAGAAT TCTTTACGGA
LC503584_E   GCTTTTTTGA AAAGATTAGG TTCGGAATTA TTGGAAGAAT TCTTTACGGA
LC503592_E   GCTTTTTTGA AAAGATTAGG TTCGGAATTA TTGGAAGAAT TCTTTACGGA

             ....|....| ....|....| ....|....| ....|....| ....|....|
                 1405       1415       1425       1435       1445
LC503540_C   AGAAGAACAA GTTTTTTCCT TGACCTTTCC AAGAGTTTCT TCTAT-----
LC503548_C   AGAAGAACAA GTTTTTTCCT TGACCTTTCC AAGAGTTTCT TCTAT-----
LC503556_C   AGAAGAACAA GTTTTTTCCT TGACCTTTCC AAGAGTTTCT TCTAT-----
LC503564_C   AGAAGAACAA GTTTTTTCCT TGACCTTTCC AAGAGTTTCT TCTAT-----
LC503572_C   AGAAGAACAA GTTTTTTCCT TGACCTTTCC AAGAGTTTCT TCTAT-----
LC503580_C   AGAAGAACAA GTTTTTTCCT TGACCTTTCC AAGAGTTTCT TCTAT-----
LC503588_C   AGAAGAACAA GTTTTTTCCT TGACCTTTCC AAGAGTTTCT TCTAT-----
LC503544_E   AGTAGAACAG GTTTTTTCCT TGACCTTTCC AAGAGTTTCT TCTATTTCTA
LC503552_E   AGTAGAACAG GTTTTTTCCT TGACCTTTCC AAGAGTTTCT TCTATTTCTA
LC503560_E   AGTAGAACAG GTTTTTTCCT TGACCTTTCC AAGAGTTTCT TCTATTTCTA
LC503568_E   AGTAGAACAG GTTTTTTCCT TGACCTTTCC AAGAGTTTCT TCTATTTCTA
LC503576_E   AGTAGAACAG GTTTTTTCCT TGACCTTTCC AAGAGTTTCT TCTATTTCTA
LC503584_E   AGTAGAACAG GTTTTTTCCT TGACCTTTCC AAGAGTTTCT TCTATTTCTA
LC503592_E   AGTAGAACAG GTTTTTTCCT TGACCTTTCC AAGAGTTTCT TCTATTTCTA

             ....|....| ....|....| ....|....| ....|....| ....|....|
                 1455       1465       1475       1485       1495
LC503540_C   -TTTGCCAAG GTTATCTAGA AGGTGTATTT GGTATTTGGA TATTATTTGT
LC503548_C   -TTTGCCAAG GTTATCTAGA AGGTGTATTT GGTATTTGGA TATTATTTGT
LC503556_C   -TTTGCCAAG GTTATCTAGA AGGTGTATTT GGTATTTGGA TATTATTTGT
LC503564_C   -TTTGCCAAG GTTATCTAGA AGGTGTATTT GGTATTTGGA TATTATTTGT
LC503572_C   -TTTGCCAAG GTTATCTAGA AGGTGTATTT GGTATTTGGA TATTATTTGT
LC503580_C   -TTTGCCAAG GTTATCTAGA AGGTGTATTT GGTATTTGGA TATTATTTGT
LC503588_C   -TTTGCCAAG GTTATCTAGA AGGTGTATTT GGTATTTGGA TATTATTTGT
LC503544_E   GTTCGAGAAG GTTATCTAGA GGTCGGATTT GGTATTTGGA TATTATTTGT
LC503552_E   GTTCGAGAAG GTTATCTAGA GGTCGGATTT GGTATTTGGA TATTATTTGT
LC503560_E   GTTCGAGAAG GTTATCTAGA GGTCGGATTT GGTATTTGGA TATTATTTGT
LC503568_E   GTTCGAGAAG GTTATCTAGA GGTCGGATTT GGTATTTGGA TATTATTTGT
LC503576_E   GTTCGAGAAG GTTATCTAGA GGTCGGATTT GGTATTTGGA TATTATTTGT
LC503584_E   GTTCGAGAAG GTTATCTAGA GGTCGGATTT GGTATTTGGA TATTATTTGT
LC503592_E   GTTCGAGAAG GTTATCTAGA GGTCGGATTT GGTATTTGGA TATTATTTGT
```

```
               ....|....| ....|....| ....|..
                  1505       1515       1525
LC503540_C    ATCAATTCTT TGGCCAATCA TGAATGA
LC503548_C    ATCAATTCTT TGGCCAATCA TGAATGA
LC503556_C    ATCAATTCTT TGGCCAATCA TGAATGA
LC503564_C    ATCAATTCTT TGGCCAATCA TGAATGA
LC503572_C    ATCAATTCTT TGGCCAATCA TGAATGA
LC503580_C    ATCAATTCTT TGGCCAATCA TGAATGA
LC503588_C    ATCAATTCTT TGGCCAATCA TGAATGA
LC503544_E    ATCAATGATT TGGCCAATCA TGAATGA
LC503552_E    ATCAATGATT TGGCCAATCA TGAATGA
LC503560_E    ATCAATGATT TGGCCAATCA TGAATGA
LC503568_E    ATCAATGATT TGGCCAATCA TGAATGA
LC503576_E    ATCAATGATT TGGCCAATCA TGAATGA
LC503584_E    ATCAATGATT TGGCCAATCA TGAATGA
LC503592_E    ATCAATGATT TGGCCAATCA TGAATGA
```

Supplementary Data S3. Alignment of ITS barcode region of *C. cinereum* and *E. sonchifolia*.

                   ....|....| ....|....| ....|....| ....|....| ....|....|
                           5   15   25   35   45
LC503555_C   GGAAGTAAAA GTCGTAACAA GGTTTCCGTA GGTGAACCTG CGGAAGGATC
LC503539_C   GGAAGTAAAA GTCGTAACAA GGTTTCCGTA GGTGAACCTG CGGAAGGATC
LC503547_C   GGAAGTAAAA GTCGTAACAA GGTTTCCGTA GGTGAACCTG CGGAAGGATC
LC503563_C   GGAAGTAAAA GTCGTAACAA GGTTTCCGTA GGTGAACCTG CGGAAGGATC
LC503571_C   GGAAGTAAAA GTCGTAACAA GGTTTCCGTA GGTGAACCTG CGGAAGGATC
LC503579_C   GGAAGTAAAA GTCGTAACAA GGTTTCCGTA GGTGAACCTG CGGAAGGATC
LC503587_C   GGAAGTAAAA GTCGTAACAA GGTTTCCGTA GGTGAACCTG CGGAAGGATC
LC503543_E   GGAAGTAAAA GTCGTAACAA GGTTTCCGTA GGTGAAC-TG CGGAAGGATC
LC503551_E   GGAAGTAAAA GTCGTAACAA GGTTTCCGTA GGTGAAC-TG CGGAAGGATC
LC503559_E   GGAAGTAAAA GTCGTAACAA GGTTTCCGTA GGTGAAC-TG CGGAAGGATC
LC503567_E   GGAAGTAAAA GTCGTAACAA GGTTTCCGTA GGTGAAC-TG CGGAAGGATC
LC503575_E   GGAAGTAAAA GTCGTAACAA GGTTTCCGTA GGTGAAC-TG CGGAAGGATC
LC503583_E   GGAAGTAAAA GTCGTAACAA GGTTTCCGTA GGTGAAC-TG CGGAAGGATC
LC503591 E   GGAAGTAAAA GTCGTAACAA GGTTTCCGTA GGTGAAC-TG CGGAAGGATC

                   ....|....| ....|....| ....|....| ....|....| ....|....|
                          55   65   75   85   95
LC503555_C   ATTGTCGAAC GTCGCATTTT AGAATGACCT GTGAATATTT ACTAACAATC
LC503539_C   ATTGTCGAAC GTCGCATTTT AGAATGACCT GTGAATATTT ACTAACAATC
LC503547_C   ATTGTCGAAC GTCGCATTTT AGAATGACCT GTGAATATTT ACTAACAATC
LC503563_C   ATTGTCGAAC GTCGCATTTT AGAATGACCT GTGAATATTT ACTAACAATC
LC503571_C   ATTGTCGAAC GTCGCATTTT AGAATGACCT GTGAATATTT ACTAACAATC
LC503579_C   ATTGTCGAAC GTCGCATTTT AGAATGACCT GTGAATATTT ACTAACAATC
LC503587_C   ATTGTCGAAC GTCGCATTTT AGAATGACCT GTGAATATTT ACTAACAATC
LC503543_E   ATTGTCGAAC CT-ATGTACT AGAATGACTT GTGAACGTGT AACAACATTT
LC503551_E   ATTGTCGAAC CT-ATGTACT AGAATGACTT GTGAACGTGT AACAACATTT
LC503559_E   ATTGTCGAAC CT-ATGTACT AGAATGACTT GTGAACGTGT AACAACATTT
LC503567_E   ATTGTCGAAC CT-ATGTACT AGAATGACTT GTGAACGTGT AACAACATTT
LC503575_E   ATTGTCGAAC CT-ATGTACT AGAATGACTT GTGAACGTGT AACAACATTT
LC503583_E   ATTGTCGAAC CT-ATGTACT AGAATGACTT GTGAACGTGT AACAACATTT
LC503591 E   ATTGTCGAAC CT-ATGTACT AGAATGACTT GTGAACGTGT AACAACATTT

                   ....|....| ....|....| ....|....| ....|....| ....|....|
                         105   115   125   135   145
LC503555_C   -GGCGTTAAG GGCTCAAG-- --GTCATGCT TGTGACTCTT TGC-----GC
LC503539_C   -GGCGTTAAG GGCTCAAG-- --GTCATGCT TGTGACTCTT TGC-----GC
LC503547_C   -GGCGTTAAG GGCTCAAG-- --GTCATGCT TGTGACTCTT TGC-----GC
LC503563_C   -GGCGTTAAG GGCTCAAG-- --GTCATGCT TGTGACTCTT TGC-----GC
LC503571_C   -GGCGTTAAG GGCTCAAG-- --GTCATGCT TGTGACTCTT TGC-----GC
LC503579_C   -GGCGTTAAG GGCTCAAG-- --GTCATGCT TGTGACTCTT TGC-----GC
LC503587_C   -GGCGTTAAG GGCTCAAG-- --GTCATGCT TGTGACTCTT TGC-----GC
LC503543_E   **T**GGTGTCCTT GGTATCAG**TC** **AT**GTCATTTG TTTGATTCTT TGG**ATGCA**AT
LC503551_E   **T**GGTGTCCTT GGTATCAG**TC** **AT**GTCATTTG TTTGATTCTT TGG**ATGCA**AT
LC503559_E   **T**GGTGTCCTT GGTATCAG**TC** **AT**GTCATTTG TTTGATTCTT TGG**ATGCA**AT
LC503567_E   **T**GGTGTCCTT GGTATCAG**TC** **AT**GTCATTTG TTTGATTCTT TGG**ATGCA**AT
LC503575_E   **T**GGTGTCCTT GGTATCAG**TC** **AT**GTCATTTG TTTGATTCTT TGG**ATGCA**AT
LC503583_E   **T**GGTGTCCTT GGTATCAG**TC** **AT**GTCATTTG TTTGATTCTT TGG**ATGCA**AT
LC503591 E   **T**GGTGTCCTT GGTATCAG**TC** **AT**GTCATTTG TTTGATTCTT TGG**ATGCA**AT

                   ....|....| ....|....| ....|....| ....|....| ....|....|
                         155   165   175   185   195
LC503555_C   CTTGCTATCC ATTGTTGTAG CGTTTATAAA AGAGCGTTAT GAATATGGTG
LC503539_C   CTTGCTATCC ATTGTTGTAG CGTTTATAAA AGAGCGTTAT GAATATGGTG
LC503547_C   CTTGCTATCC ATTGTTGTAG CGTTTATAAA AGAGCGTTAT GAATATGGTG
LC503563_C   CTTGCTATCC ATTGTTGTAG CGTTTATAAA AGAGCGTTAT GAATATGGTG
LC503571_C   CTTGCTATCC ATTGTTGTAG CGTTTATAAA AGAGCGTTAT GAATATGGTG

LC503579_C   CTTGCTATCC ATTGTTGTAG CGTTTATAAA AGAGCGTTAT GAATATGGTG
LC503587_C   CTTGCTATCC ATTGTTGTAG CGTTTATAAA AGAGCGTTAT GAATATGGTG
LC503543_E   GTTGATGTGT ATCTTTGGTA AACCCGTTGG GCGCCAATGA TTTTACATTG
LC503551_E   GTTGATGTGT ATCTTTGGTA AACCCGTTGG GCGCCAATGA TTTTACATTG
LC503559_E   GTTGATGTGT ATCTTTGGTA AACCCGTTGG GCGCCAATGA TTTTACATTG
LC503567_E   GTTGATGTGT ATCTTTGGTA AACCCGTTGG GCGCCAATGA TTTTACATTG
LC503575_E   GTTGATGTGT ATCTTTGGTA AACCCGTTGG GCGCCAATGA TTTTACATTG
LC503583_E   GTTGATGTGT ATCTTTGGTA AACCCGTTGG GCGCCAATGA TTTTACATTG
LC503591 E   GTTGATGTGT ATCTTTGGTA AACCCGTTGG GCGCCAATGA TTTTACATTG

....|....| ....|....| ....|....| ....|....| ....|....|
205 215 225 235 245
LC503555_C   GCACAATAAC AAACCCCGGC ACGGAATGTG CCAAGGAAAT GAAAACAGAA
LC503539_C   GCACAATAAC AAACCCCGGC ACGGAATGTG CCAAGGAAAT GAAAACAGAA
LC503547_C   GCACAATAAC AAACCCCGGC ACGGAATGTG CCAAGGAAAT GAAAACAGAA
LC503563_C   GCACAATAAC AAACCCCGGC ACGGAATGTG CCAAGGAAAT GAAAACAGAA
LC503571_C   GCACAATAAC AAACCCCGGC ACGGAATGTG CCAAGGAAAT GAAAACAGAA
LC503579_C   GCACAATAAC AAACCCCGGC ACGGAATGTG CCAAGGAAAT GAAAACAGAA
LC503587_C   GCACAATAAC AAACCCCGGC ACGGAATGTG CCAAGGAAAT GAAAACAGAA
LC503543_E   ACAAAACAAC AACCATCGAC ACGGCACGTG TCAAGGAAAA ATGAACATAA
LC503551_E   ACAAAACAAC AACCATCGAC ACGGCACGTG TCAAGGAAAA ATGAACATAA
LC503559_E   ACAAAACAAC AACCATCGAC ACGGCACGTG TCAAGGAAAA ATGAACATAA
LC503567_E   ACAAAACAAC AACCATCGAC ACGGCACGTG TCAAGGAAAA ATGAACATAA
LC503575_E   ACAAAACAAC AACCATCGAC ACGGCACGTG TCAAGGAAAA ATGAACATAA
LC503583_E   ACAAAACAAC AACCATCGAC ACGGCACGTG TCAAGGAAAA ATGAACATAA
LC503591 E   ACAAAACAAC AACCATCGAC ACGGCACGTG TCAAGGAAAA ATGAACATAA

....|....| ....|....| ....|....| ....|....| ....|....|
255 265 275 285 295
LC503555_C   AAAAGGTTGG GCT-TAGGAA CTCCGTTTAT GGTGTGTCTT TGGTTTTTAA
LC503539_C   AAAAGGTTGG GCT-TAGGAA CTCCGTTTAT GGTGTGTCTT TGGTTTTTAA
LC503547_C   AAAAGGTTGG GCT-TAGGAA CTCCGTTTAT GGTGTGTCTT TGGTTTTTAA
LC503563_C   AAAAGGTTGG GCT-TAGGAA CTCCGTTTAT GGTGTGTCTT TGGTTTTTAA
LC503571_C   AAAAGGTTGG GCT-TAGGAA CTCCGTTTAT GGTGTGTCTT TGGTTTTTAA
LC503579_C   AAAAGGTTGG GCT-TAGGAA CTCCGTTTAT GGTGTGTCTT TGGTTTTTAA
LC503587_C   AAAAGGTTGG GCT-TAGGAA CTCCGTTTAT GGTGTGTCTT TGGTTTTTAA
LC503543_E   GAGCGCTTGT ACC**A**TGCTTT CATCGTTCGC GGTTATTGCA GGGTATCTTG
LC503551_E   GAGCGCTTGT ACC**A**TGCTTT CATCGTTCGC GGTTATTGCA GGGTATCTTG
LC503559_E   GAGCGCTTGT ACC**A**TGCTTT CATCGTTCGC GGTTATTGCA GGGTATCTTG
LC503567_E   GAGCGCTTGT ACC**A**TGCTTT CATCGTTCGC GGTTATTGCA GGGTATCTTG
LC503575_E   GAGCGCTTGT ACC**A**TGCTTT CATCGTTCGC GGTTATTGCA GGGTATCTTG
LC503583_E   GAGCGCTTGT ACC**A**TGCTTT CATCGTTCGC GGTTATTGCA GGGTATCTTG
LC503591 E   GAGCGCTTGT ACC**A**TGCTTT CATCGTTCGC GGTTATTGCA GGGTATCTTG

....|....| ....|....| ....|....| ....|....| ....|....|
305 315 325 335 345
LC503555_C   CCTTTTGTTA TCACAAATGA CTCTCGGCAA CGGATATCTC GGCTCACGCA
LC503539_C   CCTTTTGTTA TCACAAATGA CTCTCGGCAA CGGATATCTC GGCTCACGCA
LC503547_C   CCTTTTGTTA TCACAAATGA CTCTCGGCAA CGGATATCTC GGCTCACGCA
LC503563_C   CCTTTTGTTA TCACAAATGA CTCTCGGCAA CGGATATCTC GGCTCACGCA
LC503571_C   CCTTTTGTTA TCACAAATGA CTCTCGGCAA CGGATATCTC GGCTCACGCA
LC503579_C   CCTTTTGTTA TCACAAATGA CTCTCGGCAA CGGATATCTC GGCTCACGCA
LC503587_C   CCTTTTGTTA TCACAAATGA CTCTCGGCAA CGGATATCTC GGCTCACGCA
LC503543_E   CATCTTTATA AAATAAACGA CTCTCGACAA CGGATATCTT GGCCCACGCA
LC503551_E   CATCTTTATA AAATAAACGA CTCTCGACAA CGGATATCTT GGCCCACGCA
LC503559_E   CATCTTTATA AAATAAACGA CTCTCGACAA CGGATATCTT GGCCCACGCA
LC503567_E   CATCTTTATA AAATAAACGA CTCTCGACAA CGGATATCTT GGCCCACGCA
LC503575_E   CATCTTTATA AAATAAACGA CTCTCGACAA CGGATATCTT GGCCCACGCA
LC503583_E   CATCTTTATA AAATAAACGA CTCTCGACAA CGGATATCTT GGCCCACGCA
LC503591 E   CATCTTTATA AAATAAACGA CTCTCGACAA CGGATATCTT GGCCCACGCA

```
               ....|....| ....|....| ....|....| ....|....| ....|....|
                     355        365        375        385        395
LC503555_C     TCGATGAAGA ACGTAGCAAA ATGCGATACT TGGTGTGAAT TGCAGAATCC
LC503539_C     TCGATGAAGA ACGTAGCAAA ATGCGATACT TGGTGTGAAT TGCAGAATCC
LC503547_C     TCGATGAAGA ACGTAGCAAA ATGCGATACT TGGTGTGAAT TGCAGAATCC
LC503563_C     TCGATGAAGA ACGTAGCAAA ATGCGATACT TGGTGTGAAT TGCAGAATCC
LC503571_C     TCGATGAAGA ACGTAGCAAA ATGCGATACT TGGTGTGAAT TGCAGAATCC
LC503579_C     TCGATGAAGA ACGTAGCAAA ATGCGATACT TGGTGTGAAT TGCAGAATCC
LC503587_C     TCGATGAAGA ACGTAGCAAA ATGCGATACT TGGTGTGAAT TGCAGAATCC
LC503543_E     TCGATGAAGA ACGTAGCAAA ATGCGATACT TGGTGTGAAT TGCAGAATCC
LC503551_E     TCGATGAAGA ACGTAGCAAA ATGCGATACT TGGTGTGAAT TGCAGAATCC
LC503559_E     TCGATGAAGA ACGTAGCAAA ATGCGATACT TGGTGTGAAT TGCAGAATCC
LC503567_E     TCGATGAAGA ACGTAGCAAA ATGCGATACT TGGTGTGAAT TGCAGAATCC
LC503575_E     TCGATGAAGA ACGTAGCAAA ATGCGATACT TGGTGTGAAT TGCAGAATCC
LC503583_E     TCGATGAAGA ACGTAGCAAA ATGCGATACT TGGTGTGAAT TGCAGAATCC
LC503591 E     TCGATGAAGA ACGTAGCAAA ATGCGATACT TGGTGTGAAT TGCAGAATCC

               ....|....| ....|....| ....|....| ....|....| ....|....|
                     405        415        425        435        445
LC503555_C     CGTGAACCAT CGAGTTTTTG AACGCAAGTT GCGCCCGAAG CCATTTGGTT
LC503539_C     CGTGAACCAT CGAGTTTTTG AACGCAAGTT GCGCCCGAAG CCATTTGGTT
LC503547_C     CGTGAACCAT CGAGTTTTTG AACGCAAGTT GCGCCCGAAG CCATTTGGTT
LC503563_C     CGTGAACCAT CGAGTTTTTG AACGCAAGTT GCGCCCGAAG CCATTTGGTT
LC503571_C     CGTGAACCAT CGAGTTTTTG AACGCAAGTT GCGCCCGAAG CCATTTGGTT
LC503579_C     CGTGAACCAT CGAGTTTTTG AACGCAAGTT GCGCCCGAAG CCATTTGGTT
LC503587_C     CGTGAACCAT CGAGTTTTTG AACGCAAGTT GCGCCCGAAG CCATTTGGTT
LC503543_E     CGTGAACCAT CGAGTTTTTG AACGCAAGTT GCGTCCGAAA CCTTTTGGTC
LC503551_E     CGTGAACCAT CGAGTTTTTG AACGCAAGTT GCGTCCGAAA CCTTTTGGTC
LC503559_E     CGTGAACCAT CGAGTTTTTG AACGCAAGTT GCGTCCGAAA CCTTTTGGTC
LC503567_E     CGTGAACCAT CGAGTTTTTG AACGCAAGTT GCGTCCGAAA CCTTTTGGTC
LC503575_E     CGTGAACCAT CGAGTTTTTG AACGCAAGTT GCGTCCGAAA CCTTTTGGTC
LC503583_E     CGTGAACCAT CGAGTTTTTG AACGCAAGTT GCGTCCGAAA CCTTTTGGTC
LC503591 E     CGTGAACCAT CGAGTTTTTG AACGCAAGTT GCGTCCGAAA CCTTTTGGTC

               ....|....| ....|....| ....|....| ....|....| ....|....|
                     455        465        475        485        495
LC503555_C     TAGGGCACGT CTGCTTGGGC GTCACACATC GTCTTTCTTT CCAATGT-TA
LC503539_C     TAGGGCACGT CTGCTTGGGC GTCACACATC GTCTTTCTTT CCAATGT-TA
LC503547_C     TAGGGCACGT CTGCTTGGGC GTCACACATC GTCTTTCTTT CCAATGT-TA
LC503563_C     TAGGGCACGT CTGCTTGGGC GTCACACATC GTCTTTCTTT CCAATGT-TA
LC503571_C     TAGGGCACGT CTGCTTGGGC GTCACACATC GTCTTTCTTT CCAATGT-TA
LC503579_C     TAGGGCACGT CTGCTTGGGC GTCACACATC GTCTTTCTTT CCAATGT-TA
LC503587_C     TAGGGCACGT CTGCTTGGGC GTCACACATC GTCTTTCTTT CCAATGT-TA
LC503543_E     GAGGACACGT CTGCCTGGGC GTCACATGTC ATGTCACCTC CTAACACACC
LC503551_E     GAGGACACGT CTGCCTGGGC GTCACATGTC ATGTCACCTC CTAACACACC
LC503559_E     GAGGACACGT CTGCCTGGGC GTCACATGTC ATGTCACCTC CTAACACACC
LC503567_E     GAGGACACGT CTGCCTGGGC GTCACATGTC ATGTCACCTC CTAACACACC
LC503575_E     GAGGACACGT CTGCCTGGGC GTCACATGTC ATGTCACCTC CTAACACACC
LC503583_E     GAGGACACGT CTGCCTGGGC GTCACATGTC ATGTCACCTC CTAACACACC
LC503591 E     GAGGACACGT CTGCCTGGGC GTCACATGTC ATGTCACCTC CTAACACACC

               ....|....| ....|....| ....|....| ....|....| ....|....|
                     505        515        525        535        545
LC503555_C     TGTTGAACAA CATAAATTTG TTGGAGGAGG AGATTGACTT CCCATGCCCA
LC503539_C     TGTTGAACAA CATAAATTTG TTGGAGGAGG AGATTGACTT CCCATGCCCA
LC503547_C     TGTTGAACAA CATAAATTTG TTGGAGGAGG AGATTGACTT CCCATGCCCA
LC503563_C     TGTTGAACAA CATAAATTTG TTGGAGGAGG AGATTGACTT CCCATGCCCA
LC503571_C     TGTTGAACAA CATAAATTTG TTGGAGGAGG AGATTGACTT CCCATGCCCA
LC503579_C     TGTTGAACAA CATAAATTTG TTGGAGGAGG AGATTGACTT CCCATGCCCA
LC503587_C     TGTTGAACAA CATAAATTTG TTGGAGGAGG AGATTGACTT CCCATGCCCA
LC503543_E     TCCTGATGGA GATGTCATTG TTGT-GGTGG AGATTGGCTT TCCGTTCCAG
```

```
LC503551_E   TCCTGATGGA GATGTCATTG TTGT-GGTGG AGATTGGCTT TCCGTTCCAG
LC503559_E   TCCTGATGGA GATGTCATTG TTGT-GGTGG AGATTGGCTT TCCGTTCCAG
LC503567_E   TCCTGATGGA GATGTCATTG TTGT-GGTGG AGATTGGCTT TCCGTTCCAG
LC503575_E   TCCTGATGGA GATGTCATTG TTGT-GGTGG AGATTGGCTT TCCGTTCCAG
LC503583_E   TCCTGATGGA GATGTCATTG TTGT-GGTGG AGATTGGCTT TCCGTTCCAG
LC503591_E   TCCTGATGGA GATGTCATTG TTGT-GGTGG AGATTGGCTT TCCGTTCCAG

             ....|....| ....|....| ....|....| ....|....| ....|....|
                   555        565        575        585        595
LC503555_C   TGGTGTGGTT TGTCTAAATA TTAGTCCTTT G--ATTGATG CCTGACTATA
LC503539_C   TGGTGTGGTT TGTCTAAATA TTAGTCCTTT G--ATTGATG CCTGACTATA
LC503547_C   TGGTGTGGTT TGTCTAAATA TTAGTCCTTT G--ATTGATG CCTGACTATA
LC503563_C   TGGTGTGGTT TGTCTAAATA TTAGTCCTTT G--ATTGATG CCTGACTATA
LC503571_C   TGGTGTGGTT TGTCTAAATA TTAGTCCTTT G--ATTGATG CCTGACTATA
LC503579_C   TGGTGTGGTT TGTCTAAATA TTAGTCCTTT G--ATTGATG CCTGACTATA
LC503587_C   TGGTGTGGTT TGTCTAAATA TTAGTCCTTT G--ATTGATG CCTGACTATA
LC503543_E   AGGCGCGGTT AGCTAAAATA GGAGTCCTTT TTTATTGACA CACGATTAGT
LC503551_E   AGGCGCGGTT AGCTAAAATA GGAGTCCTTT TTTATTGACA CACGATTAGT
LC503559_E   AGGCGCGGTT AGCTAAAATA GGAGTCCTTT TTTATTGACA CACGATTAGT
LC503567_E   AGGCGCGGTT AGCTAAAATA GGAGTCCTTT TTTATTGACA CACGATTAGT
LC503575_E   AGGCGCGGTT AGCTAAAATA GGAGTCCTTT TTTATTGACA CACGATTAGT
LC503583_E   AGGCGCGGTT AGCTAAAATA GGAGTCCTTT TTTATTGACA CACGATTAGT
LC503591_E   AGGCGCGGTT AGCTAAAATA GGAGTCCTTT TTTATTGACA CACGATTAGT

             ....|....| ....|....| ....|....| ....|....| ....|....|
                   605        615        625        635        645
LC503555_C   GGTGGTTGAT AAGACCTTCG TTGGAGTTGG GTGTCATAAG TCGTAAGGAT
LC503539_C   GGTGGTTGAT AAGACCTTCG TTGGAGTTGG GTGTCATAAG TCGTAAGGAT
LC503547_C   GGTGGTTGAT AAGACCTTCG TTGGAGTTGG GTGTCATAAG TCGTAAGGAT
LC503563_C   GGTGGTTGAT AAGACCTTCG TTGGAGTTGG GTGTCATAAG TCGTAAGGAT
LC503571_C   GGTGGTTGAT AAGACCTTCG TTGGAGTTGG GTGTCATAAG TCGTAAGGAT
LC503579_C   GGTGGTTGAT AAGACCTTCG TTGGAGTTGG GTGTCATAAG TCGTAAGGAT
LC503587_C   GGTGGTTGAT AAGACCTTCG TTGGAGTTGG GTGTCATAAG TCGTAAGGAT
LC503543_E   GGTGGTCGAA AAGCCCTCTT CTCGAGTTGT GTGTTCAAAT TATTTAAGAG
LC503551_E   GGTGGTCGAA AAGCCCTCTT CTCGAGTTGT GTGTTCAAAT TATTTAAGAG
LC503559_E   GGTGGTCGAA AAGCCCTCTT CTCGAGTTGT GTGTTCAAAT TATTTAAGAG
LC503567_E   GGTGGTCGAA AAGCCCTCTT CTCGAGTTGT GTGTTCAAAT TATTTAAGAG
LC503575_E   GGTGGTCGAA AAGCCCTCTT CTCGAGTTGT GTGTTCAAAT TATTTAAGAG
LC503583_E   GGTGGTCGAA AAGCCCTCTT CTCGAGTTGT GTGTTCAAAT TATTTAAGAG
LC503591_E   GGTGGTCGAA AAGCCCTCTT CTCGAGTTGT GTGTTCAAAT TATTTAAGAG

             ....|....| ....|....| ....|....| ....|....| ....|....|
                   655        665        675        685        695
LC503555_C   GAACGTGAAT AAAGACCTTC ATGCATTATC TTTGTTTGAT GCTTCGAATG
LC503539_C   GAACGTGAAT AAAGACCTTC ATGCATTATC TTTGTTTGAT GCTTCGAATG
LC503547_C   GAACGTGAAT AAAGACCTTC ATGCATTATC TTTGTTTGAT GCTTCGAATG
LC503563_C   GAACGTGAAT AAAGACCTTC ATGCATTATC TTTGTTTGAT GCTTCGAATG
LC503571_C   GAACGTGAAT AAAGACCTTC ATGCATTATC TTTGTTTGAT GCTTCGAATG
LC503579_C   GAACGTGAAT AAAGACCTTC ATGCATTATC TTTGTTTGAT GCTTCGAATG
LC503587_C   GAACGTGAAT AAAGACCTTC ATGCATTATC TTTGTTTGAT GCTTCGAATG
LC503543_E   GAAC-TCATT GATGACCCTA ATGTCTCGTC TT-GTACGAA GCGTTGATTG
LC503551_E   GAAC-TCATT GATGACCCTA ATGTCTCGTC TT-GTACGAA GCGTTGATTG
LC503559_E   GAAC-TCATT GATGACCCTA ATGTCTCGTC TT-GTACGAA GCGTTGATTG
LC503567_E   GAAC-TCATT GATGACCCTA ATGTCTCGTC TT-GTACGAA GCGTTGATTG
LC503575_E   GAAC-TCATT GATGACCCTA ATGTCTCGTC TT-GTACGAA GCGTTGATTG
LC503583_E   GAAC-TCATT GATGACCCTA ATGTCTCGTC TT-GTACGAA GCGTTGATTG
LC503591_E   GAAC-TCATT GATGACCCTA ATGTCTCGTC TT-GTACGAA GCGTTGATTG

             ....|....| ....|....| ....|....| ....|....| ....|....|
                   705        715        725        735        745
LC503555_C   CGACCCCAGG TCAGACGGGA CTACCCGCTG AGTTTAAGCA TATCAATAAG
```

```
LC503539_C   CGACCCCAGG TCAGACGGGA CTACCCGCTG AGTTTAAGCA TATCAATAAG
LC503547_C   CGACCCCAGG TCAGACGGGA CTACCCGCTG AGTTTAAGCA TATCAATAAG
LC503563_C   CGACCCCAGG TCAGACGGGA CTACCCGCTG AGTTTAAGCA TATCAATAAG
LC503571_C   CGACCCCAGG TCAGACGGGA CTACCCGCTG AGTTTAAGCA TATCAATAAG
LC503579_C   CGACCCCAGG TCAGACGGGA CTACCCGCTG AGTTTAAGCA TATCAATAAG
LC503587_C   CGACCCCAGG TCAGACGGGA CTACCCGCTG AGTTTAAGCA TATCAATAAG
LC503543_E   CGACCCCAGG TCAGGCGGGA CTACCCGCTG AGTTTAAGCA TATCAATAAG
LC503551_E   CGACCCCAGG TCAGGCGGGA CTACCCGCTG AGTTTAAGCA TATCAATAAG
LC503559_E   CGACCCCAGG TCAGGCGGGA CTACCCGCTG AGTTTAAGCA TATCAATAAG
LC503567_E   CGACCCCAGG TCAGGCGGGA CTACCCGCTG AGTTTAAGCA TATCAATAAG
LC503575_E   CGACCCCAGG TCAGGCGGGA CTACCCGCTG AGTTTAAGCA TATCAATAAG
LC503583_E   CGACCCCAGG TCAGGCGGGA CTACCCGCTG AGTTTAAGCA TATCAATAAG
LC503591_E   CGACCCCAGG TCAGGCGGGA CTACCCGCTG AGTTTAAGCA TATCAATAAG

             ....|..
                755
LC503555_C   CGGAGGA
LC503539_C   CGGAGGA
LC503547_C   CGGAGGA
LC503563_C   CGGAGGA
LC503571_C   CGGAGGA
LC503579_C   CGGAGGA
LC503587_C   CGGAGGA
LC503543_E   CGGAGGA
LC503551_E   CGGAGGA
LC503559_E   CGGAGGA
LC503567_E   CGGAGGA
LC503575_E   CGGAGGA
LC503583_E   CGGAGGA
LC503591_E   CGGAGGA
```

Supplementary Data S4. Alignment of *psb*A-*trn*H barcode region of *C. cinereum* and *E. sonchifolia*

```
               ....|....| ....|....| ....|....| ....|....| ....|....|
                   5         15         25         35         45
LC503542_C     GTTATGCATG AACGTAATGC TCATAATTTC CCTCTAGACT TAGCTGCTAT
LC503546_C     GTTATGCATG AACGTAATGC TCATAATTTC CCTCTAGACT TAGCTGCTAT
LC503558_C     GTTATGCATG AACGTAATGC TCATAATTTC CCTCTAGACT TAGCTGCTAT
LC503566_C     GTTATGCATG AACGTAATGC TCATAATTTC CCTCTAGACT TAGCTGCTAT
LC503574_C     GTTATGCATG AACGTAATGC TCATAATTTC CCTCTAGACT TAGCTGCTAT
LC503578_C     GTTATGCATG AACGTAATGC TCATAATTTC CCTCTAGACT TAGCTGCTAT
LC503590_C     GTTATGCATG AACGTAATGC TCATAATTTC CCTCTAGACT TAGCTGCTAT
LC503546_E     GTTATGCATG AACGTAATGC TCATAATTTC CCTCTAGACT TAGCTGCTAT
LC503554_E     GTTATGCATG AACGTAATGC TCATAATTTC CCTCTAGACT TAGCTGCTAT
LC503562_E     GTTATGCATG AACGTAATGC TCATAATTTC CCTCTAGACT TAGCTGCTAT
LC503570_E     GTTATGCATG AACGTAATGC TCATAATTTC CCTCTAGACT TAGCTGCTAT
LC503578_E     GTTATGCATG AACGTAATGC TCATAATTTC CCTCTAGACT TAGCTGCTAT
LC503586_E     GTTATGCATG AACGTAATGC TCATAATTTC CCTCTAGACT TAGCTGCTAT
LC503594_E     GTTATGCATG AACGTAATGC TCATAATTTC CCTCTAGACT TAGCTGCTAT

               ....|....| ....|....| ....|....| ....|....| ....|....|
                   55         65         75         85         95
LC503542_C     CGAAGCTCCA TCTACAAATG GATAAGACTT TGGTCTGATT GTATAGGAGT
LC503546_C     CGAAGCTCCA TCTACAAATG GATAAGACTT TGGTCTGATT GTATAGGAGT
LC503558_C     CGAAGCTCCA TCTACAAATG GATAAGACTT TGGTCTGATT GTATAGGAGT
LC503566_C     CGAAGCTCCA TCTACAAATG GATAAGACTT TGGTCTGATT GTATAGGAGT
LC503574_C     CGAAGCTCCA TCTACAAATG GATAAGACTT TGGTCTGATT GTATAGGAGT
LC503578_C     CGAAGCTCCA TCTACAAATG GATAAGACTT TGGTCTGATT GTATAGGAGT
LC503590_C     CGAAGCTCCA TCTACAAATG GATAAGACTT TGGTCTGATT GTATAGGAGT
LC503546_E     TGAAGCTCCA TCTACAAATG GATAAGACTT TGGTCTGATT GTATAGGAGT
LC503554_E     TGAAGCTCCA TCTACAAATG GATAAGACTT TGGTCTGATT GTATAGGAGT
LC503562_E     TGAAGCTCCA TCTACAAATG GATAAGACTT TGGTCTGATT GTATAGGAGT
LC503570_E     TGAAGCTCCA TCTACAAATG GATAAGACTT TGGTCTGATT GTATAGGAGT
LC503578_E     TGAAGCTCCA TCTACAAATG GATAAGACTT TGGTCTGATT GTATAGGAGT
LC503586_E     TGAAGCTCCA TCTACAAATG GATAAGACTT TGGTCTGATT GTATAGGAGT
LC503594_E     TGAAGCTCCA TCTACAAATG GATAAGACTT TGGTCTGATT GTATAGGAGT

               ....|....| ....|....| ....|....| ....|....| ....|....|
                  105        115        125        135        145
LC503542_C     TTTTGAACTA AAAAGGGAGC AATAGCTTTC CTCTTGTTTT ATCAAGAGGG
LC503546_C     TTTTGAACTA AAAAGGGAGC AATAGCTTTC CTCTTGTTTT ATCAAGAGGG
LC503558_C     TTTTGAACTA AAAAGGGAGC AATAGCTTTC CTCTTGTTTT ATCAAGAGGG
LC503566_C     TTTTGAACTA AAAAGGGAGC AATAGCTTTC CTCTTGTTTT ATCAAGAGGG
LC503574_C     TTTTGAACTA AAAAGGGAGC AATAGCTTTC CTCTTGTTTT ATCAAGAGGG
LC503578_C     TTTTGAACTA AAAAGGGAGC AATAGCTTTC CTCTTGTTTT ATCAAGAGGG
LC503590_C     TTTTGAACTA AAAAGGGAGC AATAGCTTTC CTCTTGTTTT ATCAAGAGGG
LC503546_E     TTTTGAACTA AAAAAGGAGC AATAAC-GCC CTCTTGTTTT ATCAAGAGGG
LC503554_E     TTTTGAACTA AAAAAGGAGC AATAAC-GCC CTCTTGTTTT ATCAAGAGGG
LC503562_E     TTTTGAACTA AAAAAGGAGC AATAAC-GCC CTCTTGTTTT ATCAAGAGGG
LC503570_E     TTTTGAACTA AAAAAGGAGC AATAAC-GCC CTCTTGTTTT ATCAAGAGGG
LC503578_E     TTTTGAACTA AAAAAGGAGC AATAAC-GCC CTCTTGTTTT ATCAAGAGGG
LC503586_E     TTTTGAACTA AAAAAGGAGC AATAAC-GCC CTCTTGTTTT ATCAAGAGGG
LC503594_E     TTTTGAACTA AAAAAGGAGC AATAAC-GCC CTCTTGTTTT ATCAAGAGGG

               ....|....| ....|....| ....|....| ....|....| ....|....|
                  155        165        175        185        195
LC503542_C     -CGTTATTGC TCCTTTTTTT TATTTAATAC TATTTGCCTT ACCCTTACAT
LC503546_C     -CGTTATTGC TCCTTTTTTT TATTTAATAC TATTTGCCTT ACCCTTACAT
LC503558_C     -CGTTATTGC TCCTTTTTTT TATTTAATAC TATTTGCCTT ACCCTTACAT
LC503566_C     -CGTTATTGC TCCTTTTTTT TATTTAATAC TATTTGCCTT ACCCTTACAT
```

LC503574_C -CGTTATTGC TCC**T**TTTTTT TATTTAATAC TATTTG**CCTT** **ACC**CTTACAT
LC503578_C -CGTTATTGC TCC**T**TTTTTT TATTTAATAC TATTTG**CCTT** **ACC**CTTACAT
LC503590_C -CGTTATTGC TCC**T**TTTTTT TATTTAATAC TATTTG**CCTT** **ACC**CTTACAT
LC503546_E **A**AGATATTGC TCC-TTTTTT TATTTAGTAC TATTTG---- ---TTTACAT
LC503554_E **A**AGATATTGC TCC-TTTTTT TATTTAGTAC TATTTG---- ---TTTACAT
LC503562_E **A**AGATATTGC TCC-TTTTTT TATTTAGTAC TATTTG---- ---TTTACAT
LC503570_E **A**AGATATTGC TCC-TTTTTT TATTTAGTAC TATTTG---- ---TTTACAT
LC503578_E **A**AGATATTGC TCC-TTTTTT TATTTAGTAC TATTTG---- ---TTTACAT
LC503586_E **A**AGATATTGC TCC-TTTTTT TATTTAGTAC TATTTG---- ---TTTACAT
LC503594_E **A**AGATATTGC TCC-TTTTTT **TATTTAGTAC** TATTTG---- ---TTTACAT

....|....| ....|....| ....|....| ....|....| ....|....|
205 215 225 235 245
LC503542_C AGTTTCT**TGA** **AAA**TTAAAAA TAAGAAGGTC T----TTTTA TAGTTT-GGT
LC503546_C AGTTTCT**TGA** **AAA**TTAAAAA TAAGAAGGTC T----TTTTA TAGTTT-GGT
LC503558_C AGTTTCT**TGA** **AAA**TTAAAAA TAAGAAGGTC T----TTTTA TAGTTT-GGT
LC503566_C AGTTTCT**TGA** **AAA**TTAAAAA TAAGAAGGTC T----TTTTA TAGTTT-GGT
LC503574_C AGTTTCT**TGA** **AAA**TTAAAAA TAAGAAGGTC T----TTTTA TAGTTT-GGT
LC503578_C AGTTTCT**TGA** **AAA**TTAAAAA TAAGAAGGTC T----TTTTA TAGTTT-GGT
LC503590_C AGTTTCT**TGA** **AAA**TTAAAAA TAAGAAGGTC T----TTTTA TAGTTT-GGT
LC503546_E AGTTTCT--- ---TTAAAAA TAACAAGGAC T**TTTA**TTTTA GAATTT**G**GGT
LC503554_E AGTTTCT--- ---TTAAAAA TAACAAGGAC T**TTTA**TTTTA GAATTT**G**GGT
LC503562_E AGTTTCT--- ---TTAAAAA TAACAAGGAC T**TTTA**TTTTA GAATTT**G**GGT
LC503570_E AGTTTCT--- ---TTAAAAA TAACAAGGAC T**TTTA**TTTTA GAATTT**G**GGT
LC503578_E AGTTTCT--- ---TTAAAAA TAACAAGGAC T**TTTA**TTTTA GAATTT**G**GGT
LC503586_E AGTTTCT--- ---TTAAAAA TAACAAGGAC T**TTTA**TTTTA GAATTT**G**GGT
LC503594_E AGTTTCT--- ---TTAAAAA TAACAAGGAC T**TTTA**TTTTA GAATTT**G**GGT

....|....| ....|....| ....|....| ....|....| ....|....|
255 265 275 285 295
LC503542_C TCGATTAGCA TGTTTTCTCT TTGTATTAAT TTATATTTCT ATTTTAAT**AT**
LC503546_C TCGATTAGCA TGTTTTCTCT TTGTATTAAT TTATATTTCT ATTTTAAT**AT**
LC503558_C TCGATTAGCA TGTTTTCTCT TTGTATTAAT TTATATTTCT ATTTTAAT**AT**
LC503566_C TCGATTAGCA TGTTTTCTCT TTGTATTAAT TTATATTTCT ATTTTAAT**AT**
LC503574_C TCGATTAGCA TGTTTTCTCT TTGTATTAAT TTATATTTCT ATTTTAAT**AT**
LC503578_C TCGATTAGCA TGTTTTCTCT TTGTATTAAT TTATATTTCT ATTTTAAT**AT**
LC503590_C TCGATTAGCA TGTTTTCTCT TTGTATTAAT TTATATTTCT ATTTTAAT**AT**
LC503546_E TTGATTCGCG TCTTTTCTAT TTGTATTCAT TTATATTAAA ATGAAAAT--
LC503554_E TTGATTCGCG TCTTTTCTAT TTGTATTCAT TTATATTAAA ATGAAAAT--
LC503562_E TTGATTCGCG TCTTTTCTAT TTGTATTCAT TTATATTAAA ATGAAAAT--
LC503570_E TTGATTCGCG TCTTTTCTAT TTGTATTCAT TTATATTAAA ATGAAAAT--
LC503578_E TTGATTCGCG TCTTTTCTAT TTGTATTCAT TTATATTAAA ATGAAAAT--
LC503586_E TTGATTCGCG TCTTTTCTAT TTGTATTCAT TTATATTAAA ATGAAAAT--
LC503594_E TTGATTCGCG TCTTTTCTAT TTGTATTCAT TTATATTAAA ATGAAAAT--

....|....| ....|....| ....|....| ....|....| ....|....|
305 315 325 335 345
LC503542_C **TATAGGTTTA** TATATCCTTT TCCCAATCTT TTATTAAGTT TGATTTCCAA
LC503546_C **TATAGGTTTA** TATATCCTTT TCCCAATCTT TTATTAAGTT TGATTTCCAA
LC503558_C **TATAGGTTTA** TATATCCTTT TCCCAATCTT TTATTAAGTT TGATTTCCAA
LC503566_C **TATAGGTTTA** TATATCCTTT TCCCAATCTT TTATTAAGTT TGATTTCCAA
LC503574_C **TATAGGTTTA** TATATCCTTT TCCCAATCTT TTATTAAGTT TGATTTCCAA
LC503578_C **TATAGGTTTA** TATATCCTTT TCCCAATCTT TTATTAAGTT TGATTTCCAA
LC503590_C **TATAGGTTTA** TATATCCTTT TCCCAATCTT TTATTAAGTT TGATTTCCAA
LC503546_E ---------- CAAATCATTT TCCCAATCTT TTATGAAGTT TTATTTCCAA
LC503554_E ---------- CAAATCATTT TCCCAATCTT TTATGAAGTT TTATTTCCAA
LC503562_E ---------- CAAATCATTT TCCCAATCTT TTATGAAGTT TTATTTCCAA
LC503570_E ---------- CAAATCATTT TCCCAATCTT TTATGAAGTT TTATTTCCAA
LC503578_E ---------- CAAATCATTT TCCCAATCTT TTATGAAGTT TTATTTCCAA
LC503586_E ---------- CAAATCATTT TCCCAATCTT TTATGAAGTT TTATTTCCAA
LC503594_E ---------- CAAATCATTT TCCCAATCTT TTATGAAGTT TTATTTCCAA

```
                ....|....| ....|....| ....|....| ....|....| ....|....|
                     355        365        375        385        395
LC503542_C      TTCAATTTTT ATCTAAAATA GATAAAAATT GGAATTTTGC TTATTTATTA
LC503546_C      TTCAATTTTT ATCTAAAATA GATAAAAATT GGAATTTTGC TTATTTATTA
LC503558_C      TTCAATTTTT ATCTAAAATA GATAAAAATT GGAATTTTGC TTATTTATTA
LC503566_C      TTCAATTTTT ATCTAAAATA GATAAAAATT GGAATTTTGC TTATTTATTA
LC503574_C      TTCAATTTTT ATCTAAAATA GATAAAAATT GGAATTTTGC TTATTTATTA
LC503578_C      TTCAATTTTT ATCTAAAATA GATAAAAATT GGAATTTTGC TTATTTATTA
LC503590_C      TTCAATTTTT ATCTAAAATA GATAAAAATT GGAATTTTGC TTATTTATTA
LC503546_E      TTCAATTTCA ATCAAAAATA GATAAAAATT CAAATTTTGC TTATTTATTA
LC503554_E      TTCAATTTCA ATCAAAAATA GATAAAAATT CAAATTTTGC TTATTTATTA
LC503562_E      TTCAATTTCA ATCAAAAATA GATAAAAATT CAAATTTTGC TTATTTATTA
LC503570_E      TTCAATTTCA ATCAAAAATA GATAAAAATT CAAATTTTGC TTATTTATTA
LC503578_E      TTCAATTTCA ATCAAAAATA GATAAAAATT CAAATTTTGC TTATTTATTA
LC503586_E      TTCAATTTCA ATCAAAAATA GATAAAAATT CAAATTTTGC TTATTTATTA
LC503594_E      TTCAATTTCA ATCAAAAATA GATAAAAATT CAAATTTTGC TTATTTATTA

                ....|....| ....|....| ....|....| ....|....| ....|....|
                     405        415        425        435        445
LC503542_C      CTTTGATTTC AGAAATAAGA AATAAATAAT ATGCTCTTTT TTTTCAT---
LC503546_C      CTTTGATTTC AGAAATAAGA AATAAATAAT ATGCTCTTTT TTTTCAT---
LC503558_C      CTTTGATTTC AGAAATAAGA AATAAATAAT ATGCTCTTTT TTTTCAT---
LC503566_C      CTTTGATTTC AGAAATAAGA AATAAATAAT ATGCTCTTTT TTTTCAT---
LC503574_C      CTTTGATTTC AGAAATAAGA AATAAATAAT ATGCTCTTTT TTTTCAT---
LC503578_C      CTTTGATTTC AGAAATAAGA AATAAATAAT ATGCTCTTTT TTTTCAT---
LC503590_C      CTTTGATTTC AGAAATAAGA AATAAATAAT ATGCTCTTTT TTTTCAT---
LC503546_E      CTTTGGTTTC ATAAAAAAGA AAGAAATTAT ATGCTCTTTT TTATGTTAGT
LC503554_E      CTTTGGTTTC ATAAAAAAGA AAGAAATTAT ATGCTCTTTT TTATGTTAGT
LC503562_E      CTTTGGTTTC ATAAAAAAGA AAGAAATTAT ATGCTCTTTT TTATGTTAGT
LC503570_E      CTTTGGTTTC ATAAAAAAGA AAGAAATTAT ATGCTCTTTT TTATGTTAGT
LC503578_E      CTTTGGTTTC ATAAAAAAGA AAGAAATTAT ATGCTCTTTT TTATGTTAGT
LC503586_E      CTTTGGTTTC ATAAAAAAGA AAGAAATTAT ATGCTCTTTT TTATGTTAGT
LC503594_E      CTTTGGTTTC ATAAAAAAGA AAGAAATTAT ATGCTCTTTT TTATGTTAGT

                ....|....| ....|....| ....|....| ....|....| ....|....|
                     455        465        475        485        495
LC503542_C      -GTTAATGGA AAAATATATT A--------- ATACTAGATA ----------
LC503546_C      -GTTAATGGA AAAATATATT A--------- ATACTAGATA ----------
LC503558_C      -GTTAATGGA AAAATATATT A--------- ATACTAGATA ----------
LC503566_C      -GTTAATGGA AAAATATATT A--------- ATACTAGATA ----------
LC503574_C      -GTTAATGGA AAAATATATT A--------- ATACTAGATA ----------
LC503578_C      -GTTAATGGA AAAATATATT A--------- ATACTAGATA ----------
LC503590_C      -GTTAATGGA AAAATATATT A--------- ATACTAGATA ----------
LC503546_E      GGTTAATGAA AAAATCTAGT AATTATAATT ATACTAGATA ATACTTACTA
LC503554_E      GGTTAATGAA AAAATCTAGT AATTATAATT ATACTAGATA ATACTTACTA
LC503562_E      GGTTAATGAA AAAATCTAGT AATTATAATT ATACTAGATA ATACTTACTA
LC503570_E      GGTTAATGAA AAAATCTAGT AATTATAATT ATACTAGATA ATACTTACTA
LC503578_E      GGTTAATGAA AAAATCTAGT AATTATAATT ATACTAGATA ATACTTACTA
LC503586_E      GGTTAATGAA AAAATCTAGT AATTATAATT ATACTAGATA ATACTTACTA
LC503594_E      GGTTAATGAA AAAATCTAGT AATTATAATT ATACTAGATA ATACTTACTA

                ....|....| ....|....| ....|....| ....|....| ....|....|
                     505        515        525        535        545
LC503542_C      ---------- ATACTAGATA AT-----AGT AGAGGGGCGG ATGTAGCCAA
LC503546_C      ---------- ATACTAGATA AT-----AGT AGAGGGGCGG ATGTAGCCAA
LC503558_C      ---------- ATACTAGATA AT-----AGT AGAGGGGCGG ATGTAGCCAA
LC503566_C      ---------- ATACTAGATA AT-----AGT AGAGGGGCGG ATGTAGCCAA
LC503574_C      ---------- ATACTAGATA AT-----AGT AGAGGGGCGG ATGTAGCCAA
LC503578_C      ---------- ATACTAGATA AT-----AGT AGAGGGGCGG ATGTAGCCAA
LC503590_C      ---------- ATACTAGATA AT-----AGT AGAGGGGCGG ATGTAGCCAA
```

```
LC503546_E   TACTGGTACT ATACTAGATA ATAGTAGAGT AGAGGGGCGG ATGTAGCCAA
LC503554_E   TACTGGTACT ATACTAGATA ATAGTAGAGT AGAGGGGCGG ATGTAGCCAA
LC503562_E   TACTGGTACT ATACTAGATA ATAGTAGAGT AGAGGGGCGG ATGTAGCCAA
LC503570_E   TACTGGTACT ATACTAGATA ATAGTAGAGT AGAGGGGCGG ATGTAGCCAA
LC503578_E   TACTGGTACT ATACTAGATA ATAGTAGAGT AGAGGGGCGG ATGTAGCCAA
LC503586_E   TACTGGTACT ATACTAGATA ATAGTAGAGT AGAGGGGCGG ATGTAGCCAA
LC503594_E   TACTGGTACT ATACTAGATA ATAGTAGAGT AGAGGGGCGG ATGTAGCCAA

             ....|....| ....|....| ....|....| ....|...
                  555        565        575        585
LC503542_C   GTGGATCAAG GCAGTGGATT GTGAATCCAC CATGCGCG
LC503546_C   GTGGATCAAG GCAGTGGATT GTGAATCCAC CATGCGCG
LC503558_C   GTGGATCAAG GCAGTGGATT GTGAATCCAC CATGCGCG
LC503566_C   GTGGATCAAG GCAGTGGATT GTGAATCCAC CATGCGCG
LC503574_C   GTGGATCAAG GCAGTGGATT GTGAATCCAC CATGCGCG
LC503578_C   GTGGATCAAG GCAGTGGATT GTGAATCCAC CATGCGCG
LC503590_C   GTGGATCAAG GCAGTGGATT GTGAATCCAC CATGCGCG
LC503546_E   GTGGATCAAG GCAGTGGATT GTGAATCCAC CATGCGCG
LC503554_E   GTGGATCAAG GCAGTGGATT GTGAATCCAC CATGCGCG
LC503562_E   GTGGATCAAG GCAGTGGATT GTGAATCCAC CATGCGCG
LC503570_E   GTGGATCAAG GCAGTGGATT GTGAATCCAC CATGCGCG
LC503578_E   GTGGATCAAG GCAGTGGATT GTGAATCCAC CATGCGCG
LC503586_E   GTGGATCAAG GCAGTGGATT GTGAATCCAC CATGCGCG
LC503594_E   GTGGATCAAG GCAGTGGATT GTGAATCCAC CATGCGCG
```

Supplementary Figure S1. Structures of four triterpenoid compounds from a distinct band of *C. cinereum* ethanolic extract. (A) *β*-amyrin, (B) taraxasterol, (C) lupeol, and (D) botulin.

A

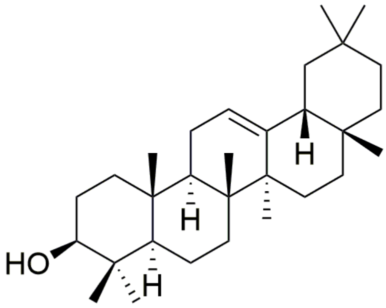

B

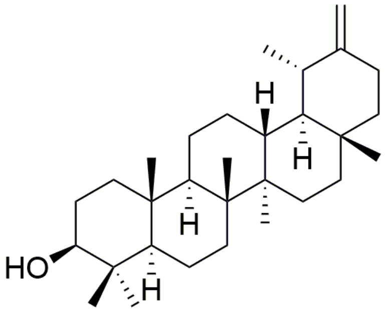

C

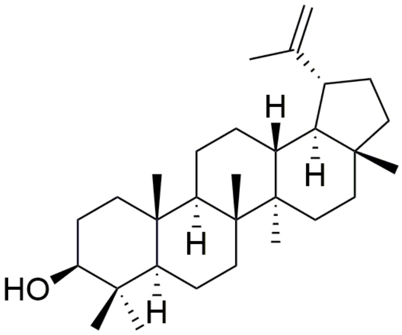

D

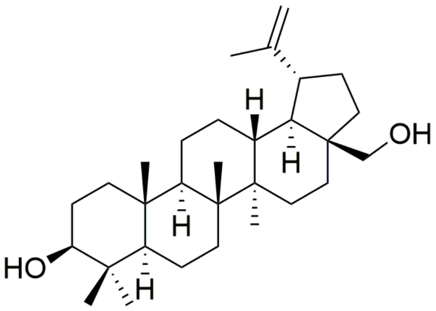

Supplementary Table S1. Primers used for DNA barcode generation and sequencing.

DNA locus	Primer name	Primer sequence (5′-3′)	References
*rbc*L	*rbc*L_aF	ATGTCACCACAAACAGAGACTAAAGC	Levin et al., 2003
	*rbc*L_R23	TTTTAGTAAAAGATTGGGCCG	Ohi-Toma et al., 2006
*mat*K	*trn*K3914F	TGGGTTGCTAACTCAATGG	Johnson & Soltis, 1994
	*trn*K 2R	AACTAGTCGGATGGAGTAG	Johnson & Soltis, 1994
	*mat*K aF	CTATATCCACTTATCTTTCAGGAG	Kato et al.,1999
	*mat*K-8R	AAAGTTCTAGCACAAGAAAGTGCA	Kato et al.,1999
*trn*H-*psb*A	*psb*A_*trn*HF	GTTATGCATGAACGTAATGCTC	Sang et al., 1997
	*psb*A-*trn*HR	CGCGCATGGTGGATTCACAATC	Sang et al., 1997
ITS	ITS1	TCCGTAGGTGAACCTGCGG	White et al., 1990
	ITS4	TCCTCCGCTTATTGATATGC	White et al., 1990

References

1. Levin, R.A. *et al.* Family-level relationships of Onagraceae based on chloroplast *rbc*L and *ndh*F data. *Am J Bot* **90**, 107-115 (2003).
2. Ohi-Toma, T. *et al.* Molecular phylogeny of *Aristolochia sensu lato* (Aristolochiaceae) based on sequences of *rbc*L, *mat*K, and *phy*A genes, with special reference to differentiation of chromosome numbers. *Systematic Botany* **31**, 481-492 (2006).
3. Johnson, L.A. & Soltis, D.E. *Mat*k DNA-sequences and phylogenetic reconstruction in Saxifragaceae S-Str. *Systematic Botany* **19**, 143-156 (1994).
4. Kato, H., Oginuma, K., Gu, Z., Hammel, B., Tobe, H. Phylogenetic relationships of Betulaceae based on *mat*K sequences with particular reference to the position of Ostryopsis. *Acta Phytotax. Geobot* **49**, 89-97 (1999).
5. Sang, T., Crawford, D. & Stuessy, T. Chloroplast DNA phylogeny, reticulate evolution, and biogeography of *Paeonia* (Paeoniaceae). *Am J Bot* **84**, 1120 (1997).
6. White, T.J., Bruns, T., Lee, S., Taylor, J. Amplification and direct sequencing of fungal ribosomal RNA genes for phylogenetics, In PCR protocols: a guide to methods and applications. (ed. N. Innis, D.G., J. Sninsky, and T. White) 315-322 (Academic Press, Inc., New York., 1990).
